# Exploring the mediating role of blood metabolites in the relationship between gut microbiota and gastric cancer risk: a Mendelian randomization study

**DOI:** 10.3389/fcimb.2024.1453286

**Published:** 2025-01-07

**Authors:** Xiaocheng Li, Huapeng Lin, Jing Peng, Jianping Gong

**Affiliations:** ^1^ Department of Hepatobiliary Surgery, The Second Affiliated Hospital of Chongqing Medical University, Chongqing, China; ^2^ Department of General Surgery, The First Affiliated Hospital of Hunan University of Medicine, Huaihua, Hunan, China; ^3^ Department of Gastroenterology and Hepatology, Shanghai Ninth People’s Hospital, Shanghai Jiao Tong University School of Medicine, Shanghai, China; ^4^ Center for Digestive Diseases Research and Clinical Translation, Shanghai Jiao Tong University, Shanghai, China; ^5^ Shanghai Key Laboratory of Gut Microecology and Associated Major Diseases Research, Shanghai Jiao Tong University, Shanghai, China

**Keywords:** gut microbiota, metabolites, gastric cancer, Mendelian randomization, causal association

## Abstract

**Background:**

Prior studies have established correlations between gut microbiota (GM) dysbiosis, circulating metabolite alterations, and gastric cancer (GC) risk. However, the causal nature of these associations remains uncertain.

**Methods:**

We utilized summary data from genome-wide association studies (GWAS) on GM (European, n=8,956), blood metabolites (European, n=120,241; East Asian, n=4,435), and GC (European, n=476,116; East Asian, n=167,122) to perform a bidirectional Mendelian randomization (MR) analysis, investigating the causal effects of GM and metabolites on GC risk. Additionally, we conducted mediation analysis (two-step MR) to identify potential metabolite mediators in the GM-GC relationship.

**Results:**

We identified twelve negative and seven positive associations between specific GM taxa and GC risk. For blood metabolites, seven traits were found to be significantly associated with reduced GC risk in the European population, with these findings subsequently validated in the East Asian cohort. Three GM taxa showed potential causal associations with five metabolic traits: the *Bacteroidia* class and *Bacteroidales* order were positively correlated with five metabolites (all *P* < 0.013), while *Bacteroides* OTU97_27 exhibited a negative correlation with one metabolite (*P* = 0.007). Two-step MR analysis indicated that total lipids in intermediate-density lipoprotein (IDL), IDL particle concentration, phospholipids in medium low-density lipoprotein (LDL), phospholipids in small LDL, and free cholesterol in small LDL indirectly influenced the association between *Bacteroidia* class/*Bacteroidales* order and GC, with mediation proportions of 1.71% (*P* = 0.048), 1.69% (*P* = 0.048), 2.05% (*P* = 0.045), 1.85% (*P* = 0.048), and 1.99% (*P* = 0.045), respectively.

**Conclusion:**

The present study provides suggestive evidence of a causal relationship between specific GM, blood metabolites, and GC risk, potentially offering new insights into GC etiology.

## 1 Introduction

Gastric cancer (GC) ranked as the fifth most common cancer and cause of cancer-related deaths globally, with 968,000 new cases and 660,000 deaths reported worldwide in 2022 (Bray et al., 2024). East Asia and Eastern Europe are the regions with the highest incidence rates globally. Extensive research suggests that gastric carcinogenesis is associated with genetic factors, *Helicobacter pylori* infection, dietary habits, and regional environmental factors, all interacting within a complex network (López et al., 2023; Noto et al., 2022; Smyth et al., 2020). The dynamic balance of human gut microbiota (GM) is closely linked to both physiological and pathological conditions and can directly or indirectly influence the carcinogenesis, treatment, and prognosis of GC (Wang et al., 2023b; Wang et al., 2023d). Increasing evidence indicates that GC patients experience changes in GM diversity and abundance, with alterations in specific microbial communities associated with GC risk (Yu et al., 2024). Additionally, some GM metabolites, such as short-chain fatty acids (SCFAs) and bile acid metabolites, have been associated with the formation and progression of GC (Noto et al., 2022; Yu et al., 2024). These metabolic products can enter systemic circulation via the gut-liver and gut-blood axes, exerting significant effects on systemic metabolism (Ahlawat et al., 2021; Hsu and Schnabl, 2023).

Blood metabolites, as critical indicators of the body’s metabolic status, serve as biomarkers for the diagnosis and prognosis of various diseases (Kurilshikov et al., 2019; Xu Z. et al., 2023). Previous studies have shown that changes in blood metabolites are closely linked to GM dysbiosis and its metabolic byproducts (Vojinovic et al., 2019). Consequently, the GM and its metabolites, along with blood metabolites, may play important roles in the carcinogenesis and development of GC (Cao et al., 2023; Lario et al., 2017). Further investigation into these relationships could not only clarify the pathogenesis of GC but also provide novel insights and methodologies for its early diagnosis and prevention. However, as current research is predominantly observational, it is prone to confounding factors such as dietary patterns, environmental influences, and reverse causality, often with small sample sizes. Additionally, ethical and practical limitations pose challenges to conducting randomized controlled studies that involve all bacterial strains.

Mendelian randomization (MR) is a powerful data analysis method for inferring causal relationships and has been widely applied in epidemiological research (Sekula et al., 2016). With advancements in sequencing technologies and the public availability of large-scale genome-wide association study (GWAS) data, numerous reliable genetic variations, such as single nucleotide polymorphisms (SNPs), are now accessible for MR studies. In this study, we used the latest GWAS datasets to conduct bidirectional MR and mediation analyses, aiming to explore the causal associations among GM, circulating metabolites, and GC risk. Additionally, we integrated metabolomics and microbiomics data to better elucidate the potential mediating role of circulating metabolites in the relationship between GM and GC.

## 2 Methods

### 2.1 Study design

**Figure 1:**
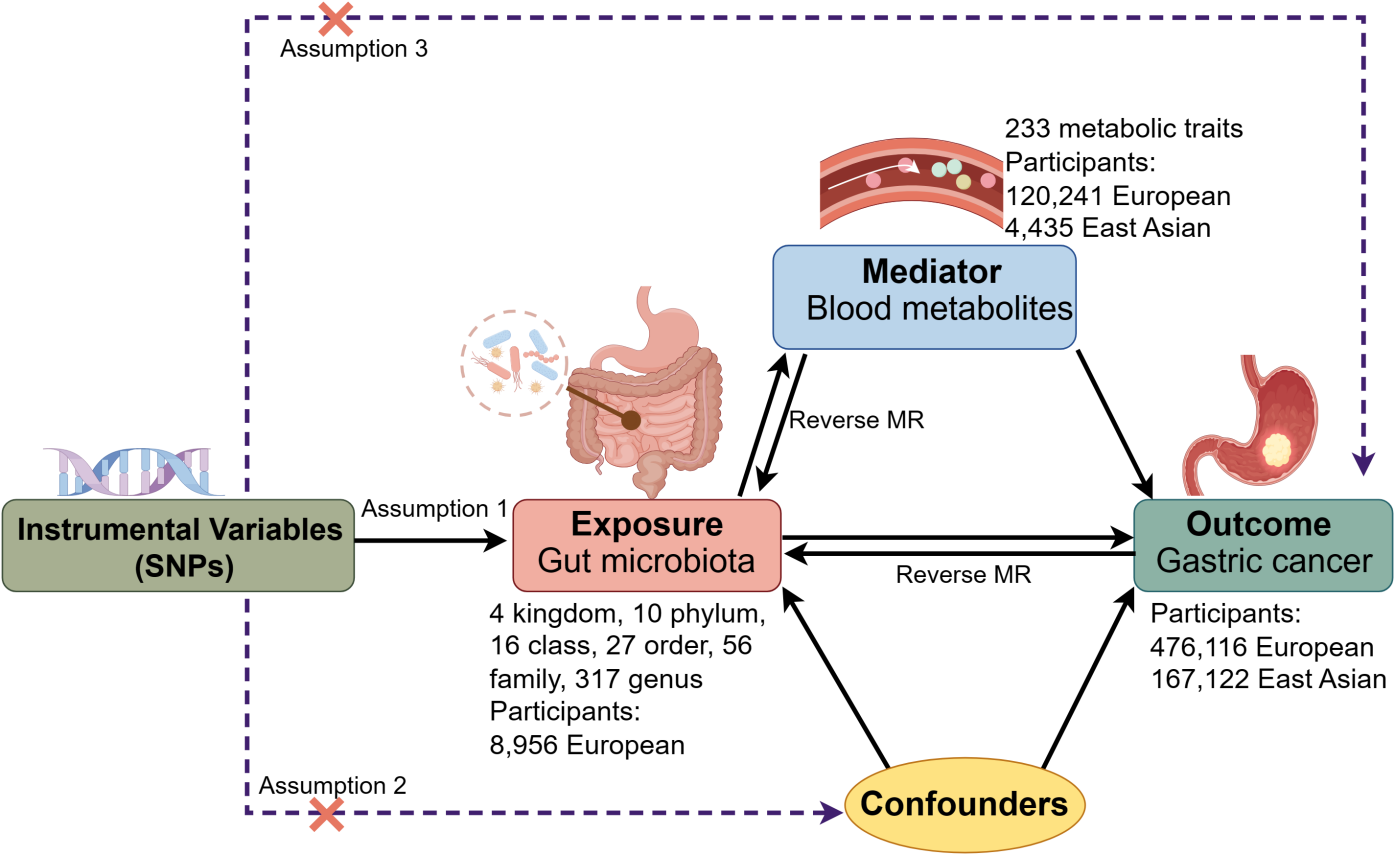
Framework and key assumptions of the Mendelian randomization (MR) analysis. SNPs, single nucleotide polymorphisms.

**Figure 1** presents the framework and the three core assumptions underlying this MR analysis. Firstly, we conducted bidirectional MR to examine the impact of GM on GC risk. Secondly, we evaluated the association between blood metabolites and GC risk, with comparative analyses conducted in European and East Asian populations. Thirdly, we assessed the reciprocal relationship between GM and blood metabolites through bidirectional MR. Finally, a two-step MR approach (Burgess et al., 2015a) was used to investigate whether GM influences GC through the mediation of blood metabolites. A detailed analysis flow is provided in **Supplementary Figure 1**. This study was conducted in accordance with the STROBE-MR checklist for the reporting of observational studies using Mendelian randomization (**Supplementary Table 1**) (Skrivankova et al., 2021).

### 2.2 Data sources and selection of genetic instruments

Detailed characteristics of all GWAS data sources are provided in **Supplementary Table 2**. Summary statistics for GM were obtained from a large-scale, single-country GWAS (Rühlemann et al., 2021). This project included 8,956 participants of European ancestry from five independent cohorts (FoCus, PopGen, KORA, SHIP, and SHIP-TREND) in Germany, with fecal samples collected from all participants. Microbial data were generated through bacterial 16S rRNA gene amplicon sequencing conducted in a wet lab in Kiel, Germany, following a standardized protocol. GWAS data from all cohorts were analyzed using a generalized linear model with logistic regression, adjusting for the top 10 genetic principal components, body mass index, sex, and age as covariates. The analysis identified 430 taxonomic units, encompassing 4 kingdoms, 10 phyla, 16 classes, 27 orders, 56 families, and 317 genera.

GWAS summary statistics for blood metabolites were collected from a large-scale genome-wide meta-analysis (Karjalainen et al., 2024), including 136,016 participants across 33 cohorts (120,241 European, 4,435 East Asian, and 11,340 South Asian individuals). This study used a consistent nuclear magnetic resonance (NMR) metabolomics platform to quantify up to 233 plasma or serum metabolic traits, including lipoprotein and lipid parameters, fatty acids and their compositions, as well as non-lipid traits (such as ketone bodies, amino acids, glycolysis/gluconeogenesis, and inflammation-related metabolites). For our analysis, 81 blood metabolite ratio traits were excluded, resulting in 152 blood metabolite concentration traits included in the final MR analysis.

Summary statistics for GC were obtained from the IEU Open GWAS Project (Elsworth et al., 2020). The GWAS data were derived from a cross-population meta-analysis incorporating phenotype data from BioBank Japan, UK Biobank, and FinnGen, with a total of 476,116 participants of European ancestry (1,029 cases and 475,087 controls) and 167,122 participants of East Asian ancestry (7,921 cases and 159,201 controls) (Sakaue et al., 2021). GC was defined based on the International Classification of Diseases (ICD) codes, specifically limited to confirmed cases of gastric carcinoma, using ICD-10: C16, ICD-9: 151, and ICD-8: 151.

When selecting instrumental variables (IVs) significantly associated with GM, we applied a relaxed significance threshold of *P* < 1×10^-5^, following previous MR studies (Li et al., 2022; Wang et al., 2023a), to ensure suitable IVs for analysis. For screening IVs significantly associated with blood metabolites and GC, we applied the conventional GWAS threshold (*P* < 5×10^-8^) to select SNPs. Subsequently, we ensured independent IV through linkage disequilibrium (LD) clumping with a stringent threshold (*r* ^2^ < 0.001, window size =10,000kb) (Ma et al., 2023). We manually filtered out SNPs associated with outcome risk factors from PhenoScanner V2 to further ensure the independence of IVs (Kamat et al., 2019). We computed the *F*-statistic (a measure of IVs’ strength) and removed SNPs with *F* < 10 to mitigate bias from weak IVs (Bowden et al., 2019; Burgess et al., 2011). After harmonizing the data, SNPs with palindromic sequences were also removed.

### 2.3 MR analysis and sensitivity analysis

Inverse variance-weighted (IVW) was used as the primary method for estimating causal effects in the two-sample MR analysis (Burgess et al., 2013; Burgess et al., 2015b), supplemented by MR-Egger (Bowden et al., 2015), weighted median (Bowden et al., 2016), and weighted mode (Hartwig et al., 2017) as additional analyses. When only one SNP was available, the Wald ratio was used as the definitive measure of causal effects, while the IVW method consolidated the Wald ratios across all genetic variants (Karjalainen et al., 2024). IVW provides the most accurate estimate of causal effects under the assumption of no horizontal pleiotropy.

A series of sensitivity analyses were conducted to detect pleiotropy and heterogeneity, ensuring the robustness of MR results. Potential horizontal pleiotropy was assessed using the Egger regression test (Bowden et al., 2015), where a *P*-value > 0.05 and an intercept close to zero indicate no evidence of pleiotropy. Heterogeneity was examined using Cochrane’s *Q* test (Burgess et al., 2013), with a *P*-value > 0.05 indicating no significant heterogeneity. When heterogeneity was detected, the random-effects IVW method was used for effect estimation, whereas the fixed-effects model was applied in its absence (Bowden et al., 2017). In cases of horizontal pleiotropy, MR Pleiotropy Residual Sum and Outlier (MR-PRESSO) tests were conducted to identify outliers contributing to pleiotropy (Verbanck et al., 2018). Outliers were removed if identified, and the MR analysis was then re-conducted. Additionally, a leave-one-out analysis was performed to detect individual SNP outliers that might disproportionately influence causal inference.

The potential mediating effect of blood metabolites was assessed using a two-step MR approach. The direct effect of each exposure on the outcome was estimated, including the effect of GM on GC risk (α), the effect of GM on blood metabolites (β1), and the effect of blood metabolites on GC risk (β2). The indirect effect was estimated using the product of coefficients method (β1 × β2), with the mediation proportion calculated as (β1 × β2)/α to quantify the mediating effect’s magnitude.

All *P*-values of IVW results were corrected using the False Discovery Rate (FDR) method. After FDR correction, *P*-values < 0.05 were considered strong causal associations, while exposures losing significance post-correction were regarded as having potential causal associations with the outcome (Shi et al., 2022). Data analyses were performed utilizing the “Mendelian Randomization” (version 0.9.0), “TwoSampleMR” (version 0.6.0), “forestplot” (version 1.1.1), and “MRPRESSO” (version 1.0) package in R Software 4.3.3.

## 3 Results

### 3.1 Genetic correlation between GM and GC

After excluding confounding-related SNPs (**Supplementary Table 3**), the number of SNPs utilized as instruments in the MR analysis ranged from 3 to 22 for the 430 GM taxa and from 3 to 6 for GC. The *F*-statistics ranged from 19.51 to 38.85 and from 990.65 to 11,306.03, respectively, indicating no evidence of weak instrument bias. Detailed information on genetic instruments is provided in **Supplementary Tables 4**, **5**.

**Figure 2:**
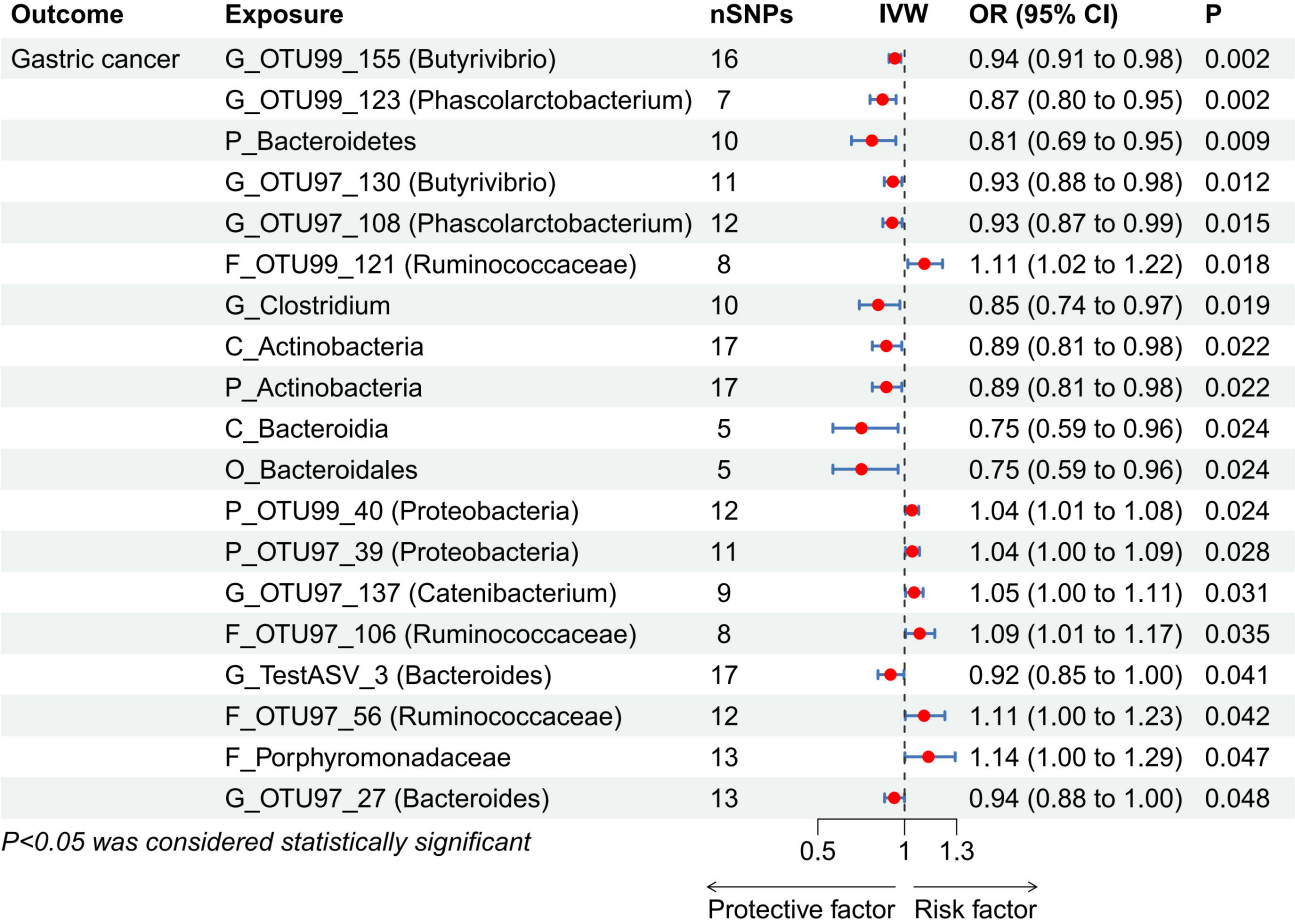
Forest plot showing associations between gut microbiota taxa and gastric cancer risk. CI, confidence interval; IVW, inverse-variance weighted; OR, odds ratios; SNPs, single nucleotide polymorphisms. “P_/C_/O_/F_/G_” represents phylum/class/order/family/genus, respectively.

In assessing the causal impact of GM on GC, MR analysis showed that two phyla, two classes, one order, and seven genera were negatively correlated with GC, while two phyla, four families, and one genus were positively correlated with GC (**Figure 2**, **Supplementary Table 6**). Among these taxa, *Butyrivibrio* OTU99_155 (odds ratio [OR], 95% confidence interval [CI]: 0.94, 0.91-0.98; *P* = 0.002) and *Phascolarctobacterium* OTU99_123 (OR, 95% CI: 0.87, 0.80-0.95; *P* = 0.002) had the strongest protective effects against GC, while *Ruminococcaceae* OTU99_121 (OR, 95% CI: 1.11, 1.02-1.22; *P* = 0.018) exhibited the most significant association with increased GC risk.

In the reverse MR analysis, evaluating the causal impact of GC on GM revealed no significant associations between GC and any GM taxa (**Supplementary Table 7**).

### 3.2 Genetically predicted blood metabolites and GC risk

For blood metabolites, the number of SNPs utilized as instruments in the MR analysis ranged from 6 to 81 in the European population and from 6 to 90 in the East Asian population. The *F*-statistics ranged from 30.34 to 6,183.51 in both populations, indicating no evidence of weak instrument bias. Detailed information on genetic instruments is provided in **Supplementary Table 8**.

In assessing the causal impacts of blood metabolites on GC risk in the European population, MR analysis identified 37 blood metabolic traits negatively correlated with GC risk, with 31 traits remaining significant after FDR correction (**Supplementary Table 9**). A repeat analysis conducted in the East Asian population revealed that 11 blood metabolic traits were significantly associated with GC risk (**Supplementary Table 10**). When combining results from both populations, seven metabolic traits (**Table 1**) were consistently associated with reduced GC risk in both European and East Asian populations. These included the concentration of intermediate-density lipoprotein (IDL) particles (OR, 95% CI: 0.85, 0.77-0.92; *P* < 0.001), total lipids in IDL (OR, 95% CI: 0.83, 0.76-0.92; *P* < 0.001), phospholipids in medium low-density lipoprotein (LDL) (OR, 95% CI: 0.82, 0.74-0.91; *P* < 0.001), phospholipids in small LDL (OR, 95% CI: 0.83, 0.75-0.91; *P* < 0.001), free cholesterol in small LDL (OR, 95% CI: 0.83, 0.75-0.91; *P* < 0.001), free cholesterol in medium LDL (OR, 95% CI: 0.84, 0.76-0.93; *P* = 0.001), and cholesterol esters in IDL (OR, 95% CI: 0.87, 0.79-0.96; *P* = 0.005).

**Table 1:** Assessing the causal relationship between blood metabolites and gastric cancer risk.

Exposure	Method	nSNP	OR (95%CI)	*P*	Heterogeneity test		Egger pleiotropy	
					Cochran’s *Q*	*P*	Intercept	*P*
Phospholipids in small LDL	IVW	71	0.83(0.75-0.91)	1.05E-4	67.751	0.554		
	MR Egger	71	0.85(0.73-0.98)	0.028	67.580	0.526	-0.002	0.681
Phospholipids in medium LDL	IVW	68	0.82(0.74-0.91)	1.57E-4	72.457	0.303		
	MR Egger	68	0.81(0.70-0.95)	0.012	72.427	0.274	7.29E-4	0.870
Total lipids in IDL	IVW	71	0.83(0.76-0.92)	1.67E-4	75.783	0.297		
	MR Egger	71	0.83(0.72-0.95)	0.011	75.765	0.269	0.001	0.899
Concentration of IDL particles	IVW	68	0.85(0.77-0.92)	2.43E-4	68.276	0.434		
	MR Egger	68	0.83(0.73-0.96)	0.011	68.189	0.403	0.001	0.773
Free cholesterol in small LDL	IVW	67	0.83(0.75-0.91)	1.61E-4	65.519	0.494		
	MR Egger	67	0.84(0.73-0.97)	0.022	65.428	0.462	-0.001	0.765
Free cholesterol in medium LDL	IVW	70	0.84(0.76-0.93)	0.001	74.854	0.294		
	MR Egger	70	0.84(0.76-0.93)	0.041	74.566	0.273	-0.002	0.610
Cholesterol esters in IDL	IVW	70	0.87(0.79-0.96)	0.005	74.683	0.299		
	MR Egger	70	0.83(0.72-0.96)	0.016	74.005	0.289	0.004	0.433

CI, confidence interval; IVW, inverse variance-weighted; IDL, intermediate-density lipoprotein; LDL, low-density lipoprotein; MR, Mendelian randomization; OR, odds ratio; SNPs, single nucleotide polymorphisms.

### 3.3 Genetic correlation between GM and blood metabolites

Bidirectional MR analysis was conducted to assess correlations between 19 GM taxa with causal associations to GC and 7 blood metabolic traits. After FDR correction, IVW results identified significant associations between 3 GM taxa and 5 metabolic traits. Specifically, the *Bacteroidia* class/*Bacteroidales* order was positively correlated with 5 metabolic traits, while *Bacteroides* OTU97_27 showed a negative correlation with free cholesterol in small LDL (**Table 2**, **Supplementary Table 11**). In the reverse MR analysis, no significant effect of metabolites on GM was observed (**Supplementary Table 12**).

**Table 2:** Assessing the causal relationships between gut microbiota and blood metabolites.

Exposure	Mediator	Method	nSNP	OR (95%CI)	*P*	Cochran’s *Q*	Q_*P*
C_*Bacteroidia*/O_*Bacteroidales*	Total lipids in IDL	IVW	5	1.04(1.01-1.07)	0.007	3.484	0.480
		MR Egger	5	1.06(1.01-1.11)	0.096	2.624	0.453
	Concentration of IDL particles	IVW	5	1.04(1.01-1.07)	0.006	3.995	0.407
		MR Egger	5	1.06(1.01-1.11)	0.092	3.041	0.385
	Phospholipids in medium LDL	IVW	5	1.04(1.01-1.08)	0.006	4.650	0.325
		MR Egger	5	1.05(0.99-1.11)	0.182	4.524	0.210
	Phospholipids in small LDL	IVW	5	1.04(1.01-1.07)	0.004	3.907	0.419
		MR Egger	5	1.04(0.99-1.10)	0.236	3.898	0.273
	Free cholesterol in small LDL	IVW	5	1.03(1.01-1.07)	0.012	2.398	0.663
		MR Egger	5	1.04(0.99-1.08)	0.230	2.394	0.495
G_OTU97_27 (*Bacteroides*)	Free cholesterol in small LDL	IVW	13	0.98(0.97-1.00)	0.007	15.720	0.204
		MR Egger	13	0.98(0.92-1.04)	0.500	15.702	0.153

CI, confidence interval; IVW, inverse variance-weighted; IDL, intermediate-density lipoprotein; LDL, low-density lipoprotein; MR, Mendelian randomization; OR, odds ratio; SNPs, single nucleotide polymorphisms.

### 3.4 Mediation analysis linking GM with GC via blood metabolites

Mediation MR was performed to examine the mediating effect of blood metabolites on the association between GM and GC. Results indicated that total lipids in IDL, concentration of IDL particles, phospholipids in medium LDL, phospholipids in small LDL, and free cholesterol in small LDL exhibited indirect effects on the relationship between *Bacteroidia* class/*Bacteroidales* order and GC, with mediation proportions of 1.71%, 1.69%, 2.05%, 1.85%, and 1.99%, respectively. Free cholesterol in small LDL did not show a mediating effect between *Bacteroides* OTU97_27 and GC (*P* = 0.100) (**Table 3**).

**Table 3:** Mediation analysis of the effect of gut microbiota on gastric cancer via blood metabolites.

Exposure	Mediator	Total effect Beta (95% CI)	Direct effect Beta (95% CI)	Intermediary effect	
				Beta (95% CI)	*P*
C_*Bacteroidia*/O_*Bacteroidales*	Total lipids in IDL	-0.286(-0.533, -0.038)	-0.281(-0.528, -0.033)	-0.005(-0.010, 0.000)	0.048
	Concentration of IDL particles	-0.286(-0.533, -0.038)	-0.281(-0.529, -0.033)	-0.005(0.010, 0.000)	0.048
	Phospholipids in medium LDL	-0.286(-0.533, -0.038)	-0.280(-0.528, -0.032)	-0.006(-0.012, 0.000)	0.045
	Phospholipids in small LDL	-0.286(-0.533, -0.038)	-0.280(-0.528, -0.033)	-0.005(-0.011, 0.000)	0.048
	Free cholesterol in small LDL	-0.286(-0.533, -0.038)	-0.280(-0.528, -0.032)	-0.006(-0.011, 0.000)	0.045
G_OTU97_27 (*Bacteroides*)	Free cholesterol in small LDL	-0.059(-0.115, -0.002)	-0.061(-0.118, -0.004)	0.002(-0.001, 0.005)	0.100

CI, confidence interval; IDL, intermediate-density lipoprotein; LDL, low-density lipoprotein.

### 3.5 Sensitivity analyses

Sensitivity analyses, including Cochrane *Q* test and MR-Egger test, revealed no evidence of horizontal pleiotropy or significant heterogeneity in the MR analyses (**Supplementary Table 13**). Additionally, the MR-PRESSO test identified no outliers or pleiotropy (**Supplementary Table 13**). Scatter plots illustrating the linear regression for each SNP provide a visual overview of the association effects between SNPs and phenotypes (**Supplementary Figures 2**-**4**). Funnel plots show that most MR analyses are free from significant bias (**Supplementary Figures 5**-**7**). However, when GM was used as the exposure variable, certain microbiota taxa displayed uneven SNP distribution in the funnel plot, likely due to the limited number of SNPs available for these taxa, suggesting the possibility of small-sample bias. The forest plot illustrates the causal effect of individual SNPs on the outcome (**Supplementary Figures 8**-**10**). Leave-one-out analysis did not identify any anomalous SNPs (**Supplementary Figures 11**-**13**). Collectively, these sensitivity analyses support the statistical reliability of the analytical results and enhance the credibility of the conclusions.

## 4 Discussion

In this large-scale MR study, we identified potential causal relationships between 19 GM taxa and 7 blood metabolic traits with GC and simultaneously explored their potential interactions. Using a two-step MR method for mediation analysis, we found that five blood metabolic traits may mediate the causal relationship between GM and GC. These findings suggest that the protective effects of the *Bacteroidia* class and *Bacteroidales* order in reducing GC risk may be partially attributed to their influence on the concentrations of five blood metabolites.

In our study, *Bacteroides* exhibited a negative correlation with GC across taxonomic levels from phylum to genus, suggesting a potential protective role for *Bacteroides* in GC patients. Its reduced abundance may be associated with gastric carcinogenesis. An observational study examining fecal samples from 22 healthy participants and 20 GC patients reported findings consistent with ours, revealing a significant decrease in *Bacteroides* abundance from phylum to genus in GC patients compared to healthy controls (Liang et al., 2019). Additionally, an MR study conducted in a different population further supports our findings (Xie et al., 2023). *Bacteroides* is a key commensal bacterium in the human gut and a major producer of SCFAs, which play a crucial role in maintaining immune homeostasis within the gastrointestinal tract (Raoul et al., 2024; Wexler, 2007). In gastrointestinal neoplasms, both SCFA levels and the abundance of SCFA-producing bacteria are significantly reduced (Dong et al., 2023; Yu et al., 2024). Previous studies have shown that direct supplementation of dietary fiber and probiotics or the transplantation of fecal microbiota to alter GM composition can enhance SCFA levels and suppress neoplasm development (Dong et al., 2023; Yu et al., 2024).

Growing evidence suggests that SCFAs, as natural histone deacetylase (HDAC) inhibitors produced by GM, play a critical role across various immune cells. SCFAs can promote macrophage differentiation and enhance antimicrobial activity by inhibiting HDAC3 (Schulthess et al., 2019). They also induce IL-10 expression in T cells and enhance B cell activation, thus promoting immune tolerance and maintaining gut microbial balance (Kim, 2023). Recent experimental studies have found that butyrate regulates CD8+ T cell cytotoxicity through the G protein-coupled receptor 109A (GPR109A) and homologous domain protein homologous box (HOPX) pathway, resulting in enhanced anti-cancer activity against tumor cells in the GC microenvironment (Yu et al., 2024). Notably, butyrate and other SCFAs have direct effects on the immune system and also indirectly suppress the development of gastrointestinal tumors, including GC, by preserving gut barrier function and preventing microbial dysbiosis (Gou et al., 2024; Kaźmierczak-Siedlecka et al., 2022). These findings suggest that increasing SCFA levels through dietary or probiotic interventions may have significant potential in reducing GC risk (Gou et al., 2024; Yu et al., 2024).

In our study, *Butyrivibrio* and *Phascolarctobacterium* genera exhibited the most significant negative correlations with GC risk. Both genera belong to the *Firmicutes* phylum and are producers of SCFAs. *Butyrivibrio*, a beneficial gut bacterium, generates butyrate, a metabolite linked to numerous health-promoting effects, including the maintenance of the intestinal barrier, energy homeostasis, and anti-inflammatory and antioxidant activities (Hodgkinson et al., 2023; Zhang et al., 2021). A cross-sectional study reported a significant increase in *Butyrivibrio* abundance in patients’ intestines following subtotal gastrectomy for early-stage GC (Lin et al., 2018). However, research on the association between *Butyrivibrio* abundance and GC risk remains limited, warranting further clinical studies to elucidate their relationship. *Phascolarctobacterium*, primarily utilizing succinate produced by *Bacteroides* as a nutritional substrate, may interact with *Bacteroides* in the gut (Ikeyama et al., 2020; Watanabe et al., 2012). We therefore hypothesize that *Phascolarctobacterium* abundance may decrease along with the reduction of *Bacteroides* in the gut of GC patients. A recent study indeed found a significant reduction in *Phascolarctobacterium* abundance among GC patients (Yu et al., 2024), which indirectly supports its protective effect against GC.

The *Ruminococcus* genus includes both beneficial and harmful species, and its impact on GC risk may vary across populations and classification levels. Our findings indicate a positive correlation between the *Ruminococcaceae* family and GC risk. A fecal microbiota analysis of a European cohort similarly revealed a marked increase in *Ruminococcus* abundance among GC patients (Youssef et al., 2018). Conversely, studies analyzing fecal samples from East Asian populations reported conflicting results (Lee et al., 2024; Yu et al., 2024). Consequently, additional research with larger, ethnically diverse samples is necessary to clarify the relationship between *Ruminococcus* and GC risk.

Our MR study identified several blood metabolic traits with causal effects on GC risk, providing insights from a genetic perspective. For instance, elevated levels of phospholipids and free cholesterol in small and medium LDL were associated with a decreased GC risk. Similarly, higher levels of total lipids, cholesterol esters in IDL, and increased IDL particle concentrations correlated with reduced GC risk. Prior research has predominantly examined traditional blood markers linked to lipoproteins and lipids in relation to GC (Qiu et al., 2023). A meta-analysis summary has demonstrated that serum high-density lipoprotein cholesterol (HDL-C) and total cholesterol (TC) levels are negatively associated with GC risk, while low-density lipoprotein cholesterol (LDL-C) and triglycerides (TG) levels show no association with GC risk (Xu S. et al., 2023). Our study is the first to examine the causal relationship between circulating lipoprotein subclasses and GC risk, providing new insights into the intricate biochemical mechanisms underlying lipid metabolism dysregulation in GC patients.

To date, no studies have directly linked the *Bacteroidia* class or the *Bacteroidales* order to circulating lipoprotein subfractions. Previous observational studies have confirmed associations between specific GM taxa and subfractions of very-low-density lipoprotein (VLDL) and HDL, offering some understanding of the GM’s influence on host systemic metabolism (Vojinovic et al., 2019). The mechanisms by which GM may influence circulating metabolites remain incompletely understood, but they likely involve GM’s role in bile acid and SCFA metabolism (Ghazalpour et al., 2016). Prior studies suggest that bacteria-derived bile acids entering the bloodstream can participate in systemic lipid metabolism (Allayee and Hazen, 2015), while SCFAs such as butyrate, acetate, and propionate may also influence lipid biosynthesis (Allayee and Hazen, 2015). Through mediation analysis, we further explored the potential mediating effects of circulating metabolites on the association between GM and GC. Overall, the mediation proportions of these blood metabolite categories in the GM-GC relationship were relatively low. These modest mediation percentages likely reflect the complex and multifactorial nature of GC etiology, suggesting that blood metabolites play a limited mediating role in the relationship between GM and GC. Furthermore, the similarity in mediation proportions observed among certain metabolite categories may be attributed to shared lipid metabolism pathways and the overlapping biological functions of these lipid components, leading to parallel mediating effects on GC risk. From the funnel plots in our study, we observed that when GM was used as the exposure, the SNP distribution for certain taxa was uneven, indicating potential heterogeneity in the results. As with most MR studies on GM (Chen et al., 2023; Li et al., 2023; Zhou et al., 2023), this heterogeneity may be due to the limited number of available IVs. Consequently, the observed associations between these taxa and GC cannot entirely rule out the influence of chance or unmeasured confounding factors. Further research is therefore needed to confirm the definitive causal relationships between these taxa and GC.

This study has several strengths. We utilized the most recent and largest GWAS summary data available for blood metabolites and GC, with over 136,000 participants for metabolite data, 476,116 European participants, and 167,122 East Asian participants for GC data. Furthermore, we validated associations between blood metabolites and GC in both European and East Asian populations, ensuring robust statistical power. This study is also the first to investigate causal relationships among human GM, blood metabolites, and GC from a genetic standpoint, minimizing the confounding effects commonly encountered in observational studies.

Conversely, several potential limitations of this study should be acknowledged. First, our analysis relied exclusively on GM data from European populations. Given the substantial variability in GM composition across populations and ethnic groups, the generalizability of our findings may be restricted. Second, the GM data were generated using 16S rRNA sequencing, which inherently limits taxonomic resolution and hampers differentiation between closely related bacterial species (Rühlemann et al., 2021). Additionally, our study was restricted to bacterial taxa, while other non-bacterial microbiome constituents, such as fungi, viruses, archaea, and protozoa, are increasingly recognized for their roles in gastrointestinal health and disease (Jaswal et al., 2023). Expanding future analyses to encompass these groups could provide a more comprehensive understanding of the microbiome’s role in GC. Third, although we used a bidirectional MR approach to evaluate causal relationships, the power of the reverse MR analysis may be limited by the relatively low number of GC cases, potentially impacting the robustness of these reverse causal findings. Thus, we recommend cautious interpretation of these results. Additionally, although our MR analysis detected no pleiotropy, it cannot be entirely ruled out, especially as GM influences luminal metabolites that may not persist in the bloodstream or be detectable at all sampling points. Lastly, we identified GM taxa with nominally significant associations with GC, though these associations lost significance after applying multiple correction tests. Given the complex interdependencies among GM taxa (Faust and Raes, 2012), multiple testing corrections may be overly conservative in high-dimensional datasets (Wang et al., 2023c). Therefore, while not meeting the threshold for strict significance, the nominal associations identified should not be dismissed entirely, particularly as prior studies have corroborated that some of these taxa are associated with GC risk. Future studies with larger sample sizes and refined correction techniques may help to confirm these associations more robustly.

## 5 Conclusions

In summary, our MR analysis using genetic instruments identified several blood metabolites with potential causal associations to GC risk. Additionally, it provided suggestive evidence for a causal relationship between GM and GC, as well as the potential mediating role of blood metabolites in the GM-GC association. While this exploratory study offers valuable insights into the complex interactions between GM, blood metabolites, and GC, further research is essential to validate these causal relationships and elucidate the underlying mechanisms. Such efforts could ultimately inform novel GC treatment strategies centered on GM modulation and targeted metabolite interventions.

## Data Availability

The original contributions presented in the study are included in the article/**Supplementary Material**. Further inquiries can be directed to the corresponding author.
