# Supplementary material for: Exploring the mediating role of blood metabolites in the relationship between gut microbiota and gastric cancer risk: a Mendelian randomization study
Source: Front Cell Infect Microbiol. 2025 Jan 7;14:1453286. doi: 10.3389/fcimb.2024.1453286 (PMC11747456; doi:10.3389/fcimb.2024.1453286)
Supplement: Supplementary file 2 [file DataSheet2.docx]

**Supplementary Figure**

**Supplementary Figure 1: Flow chart of the design of this Mendelian randomization study.**

**Supplementary Figure 2: Scatter plots of SNP effects on gut microbiota versus gastric cancer.**

**Supplementary Figure 3: Scatter plots of SNP effects on blood metabolites versus gastric cancer.**

**Supplementary Figure 4: Scatter plots of SNP effects on gut microbiota versus blood metabolites.**

**Supplementary Figure 5: Funnel plot for gut microbiota and gastric cancer.**

**Supplementary Figure 6: Funnel plot for blood metabolites and gastric cancer.**

**Supplementary Figure 7: Funnel plot for gut microbiota and blood metabolites.**

**Supplementary Figure 8: Forest plot of the causal effects of gut microbiota associated SNPs on gastric cancer.**

**Supplementary Figure 9: Forest plot of the causal effects of blood metabolites associated SNPs on gastric cancer.**

**Supplementary Figure 10: Forest plot of the causal effects of gut microbiota associated SNPs on blood metabolites.**

**Supplementary Figure 11: Leave-one-out sensitivity analysis of Mendelian randomization for gut microbiota and gastric cancer.**

**Supplementary Figure 12: Leave-one-out sensitivity analysis of Mendelian randomization for blood metabolites and gastric cancer.**

**Supplementary Figure 13: Leave-one-out sensitivity analysis of Mendelian randomization for gut microbiota and blood metabolites.**


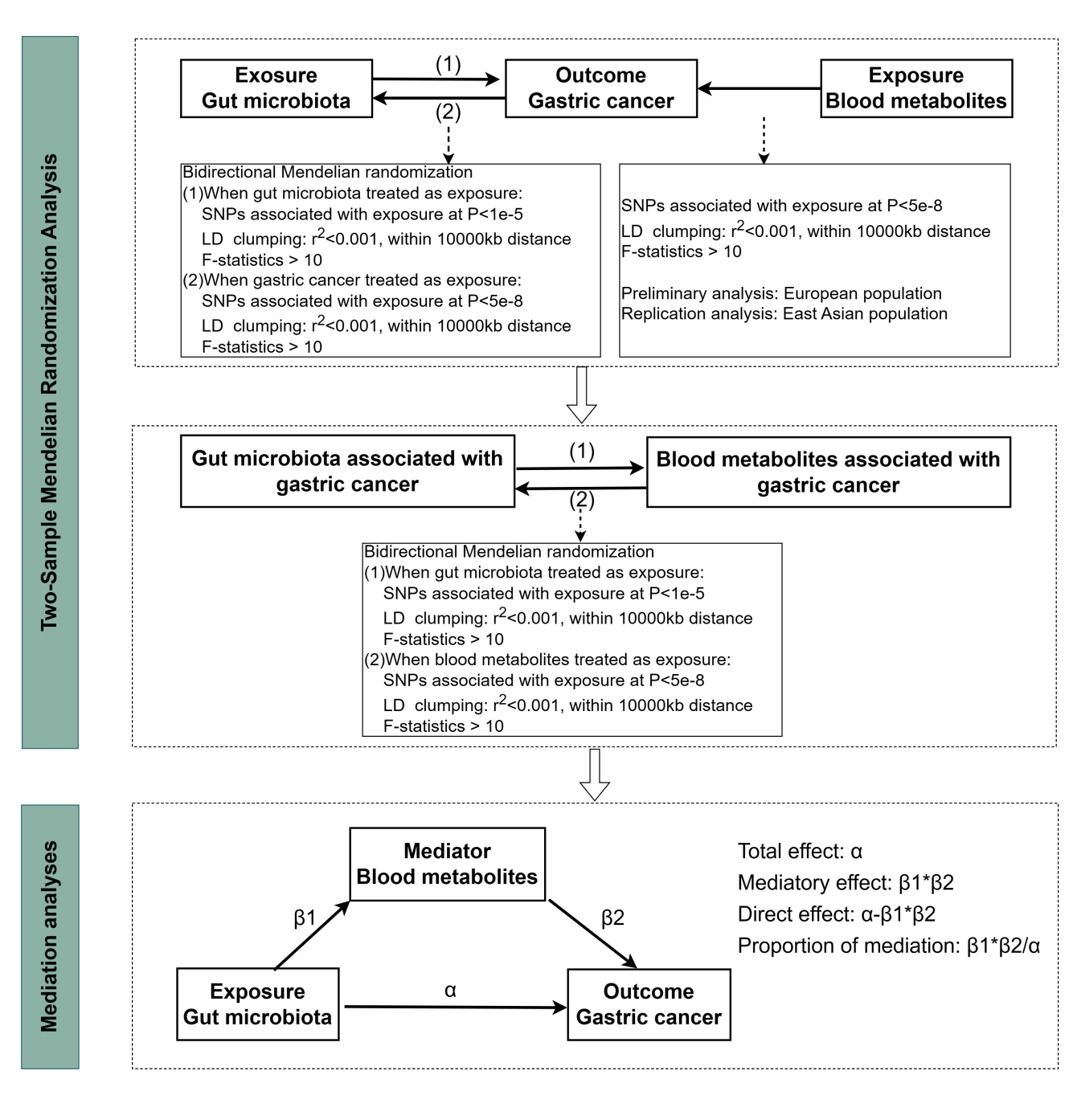


**Supplementary Figure 1: Flow chart of the design of this Mendelian randomization study**. All Mendelian randomization study comply with three fundamental assumptions: Assumption 1, genetic instruments are strongly associated with the exposures of interest; Assumption 2, genetic instruments are independent of confounding factors; Assumption 3, genetic instruments are not associated with outcome and affect outcome only via exposures. SNPs, single nucleotide polymorphisms; LD, linkage disequilibrium.

(A) C_*Actinobacteria* versus GC (B) C_*Bacteroidia* versus GC


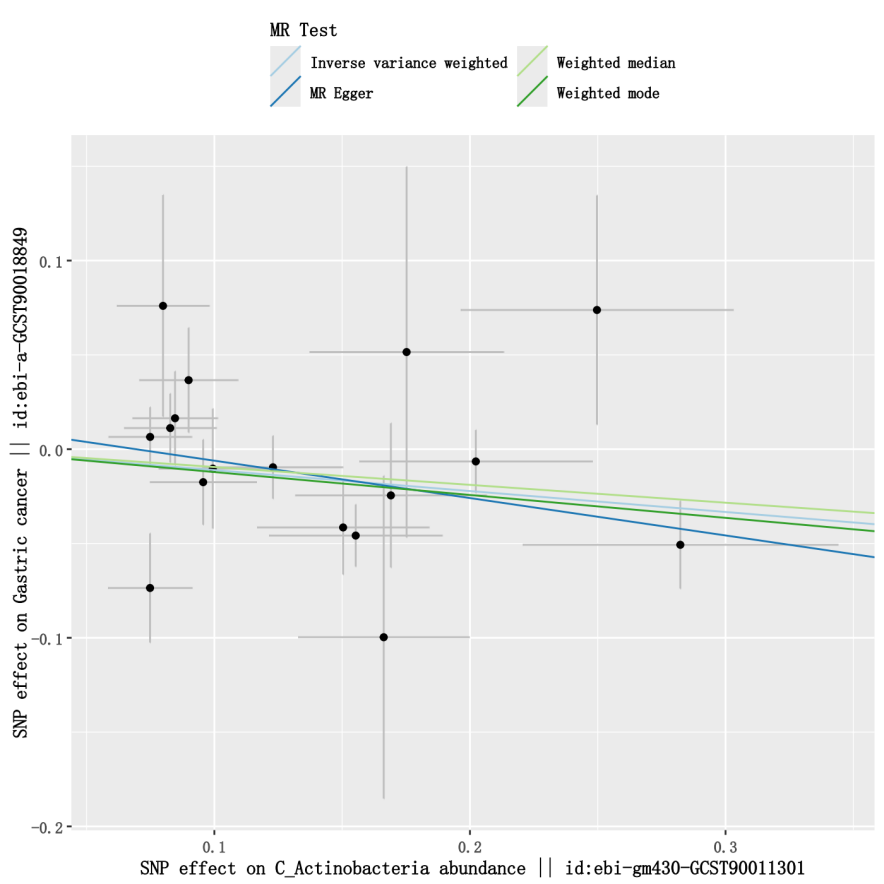

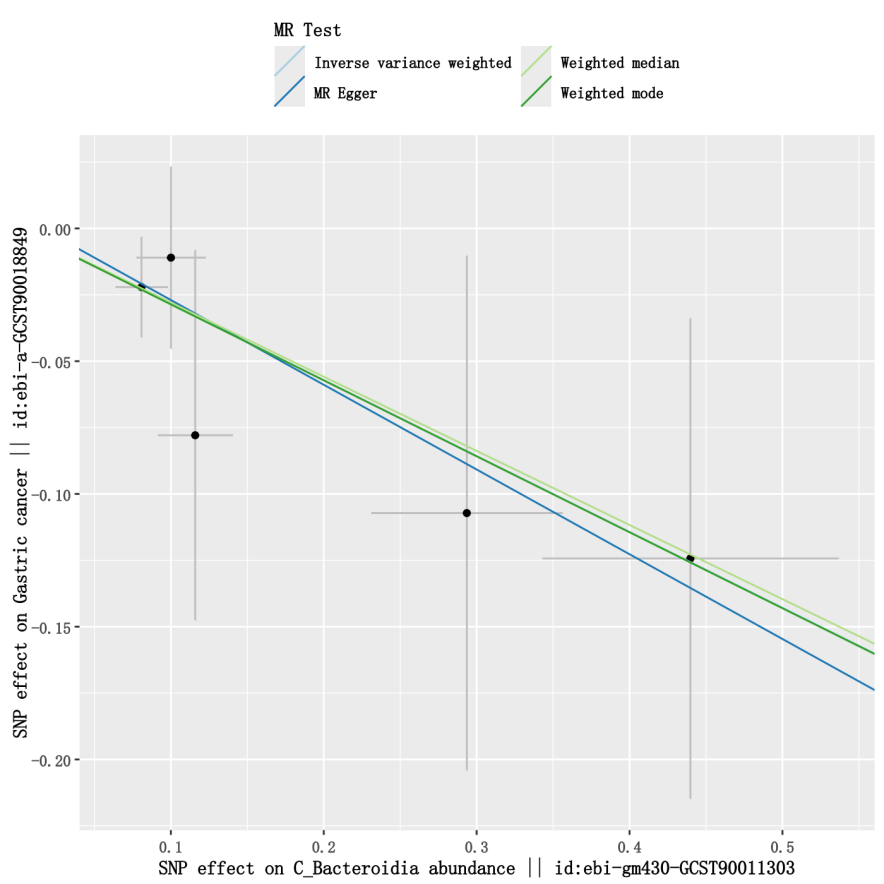

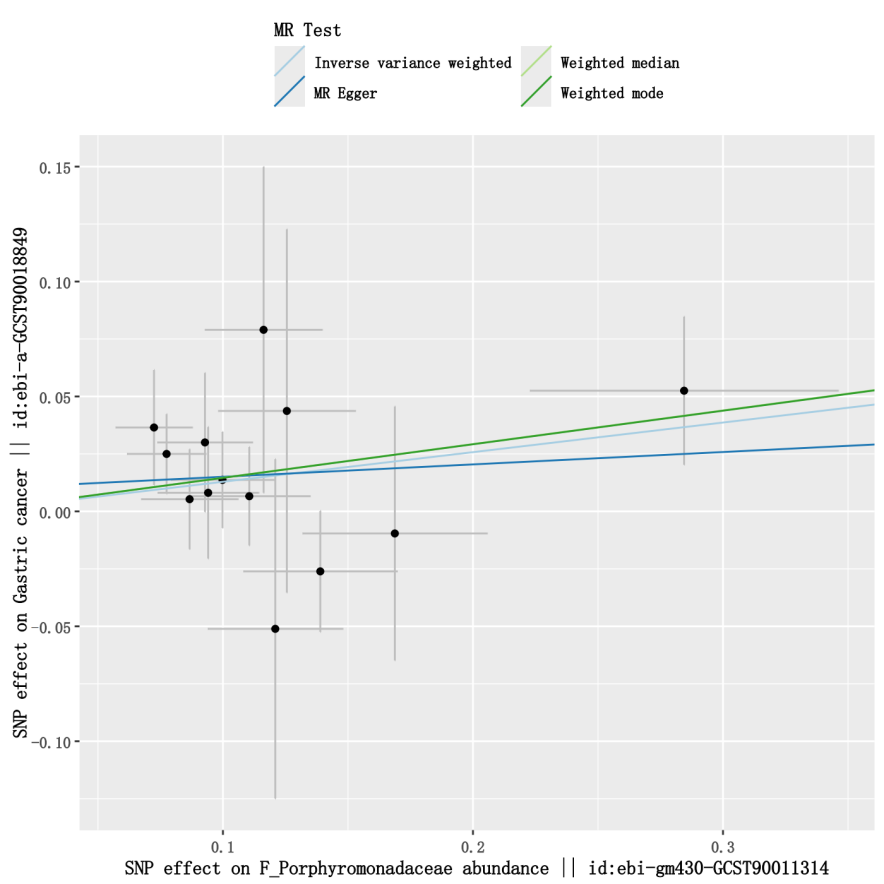

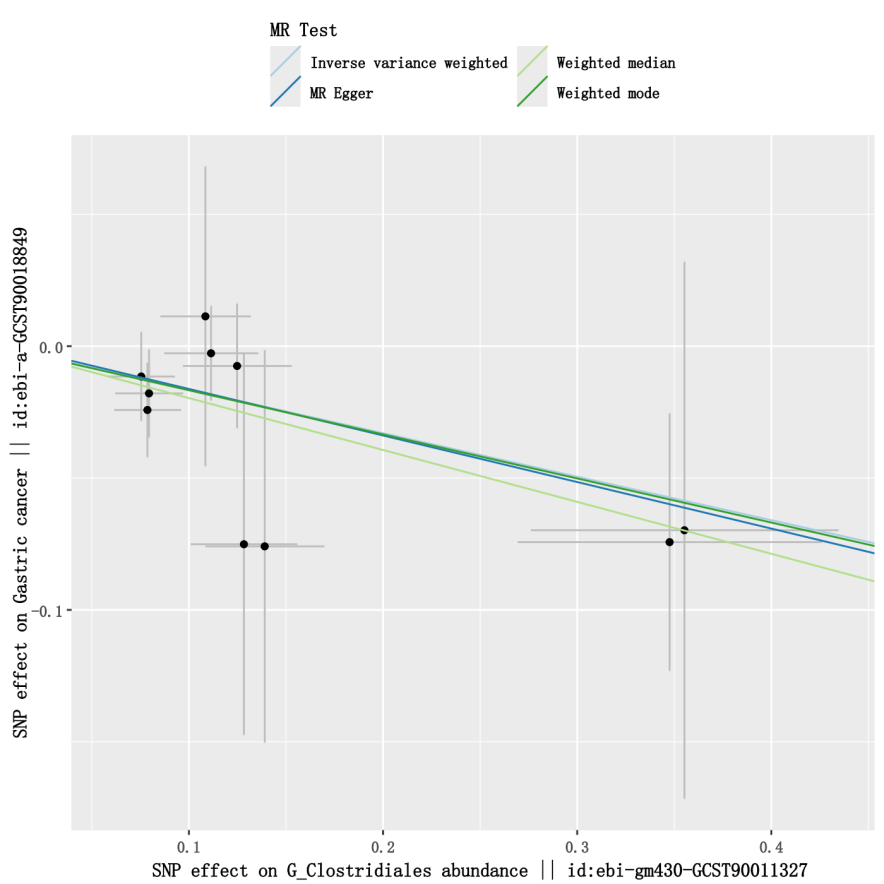


(C) F_*Porphyromonadaceae* versus GC (D) G_*Clostridiales* versus GC

(E) O_*Bacteroidales* versus GC (F) OTU97_106 (*Ruminococcaceae*) versus GC


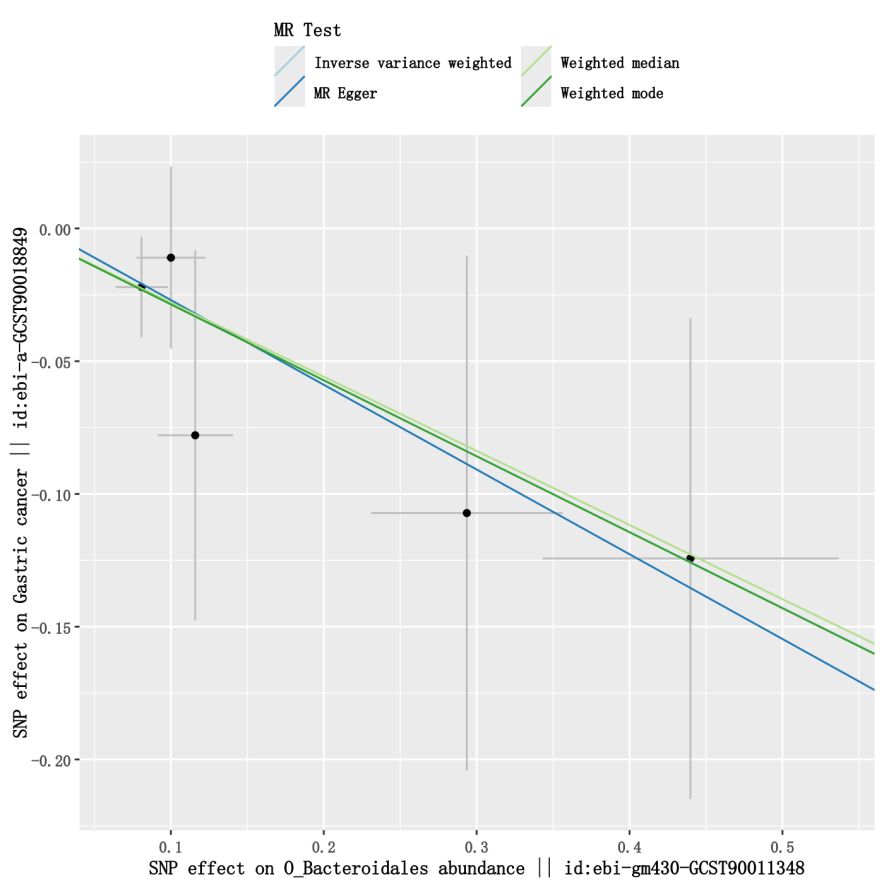

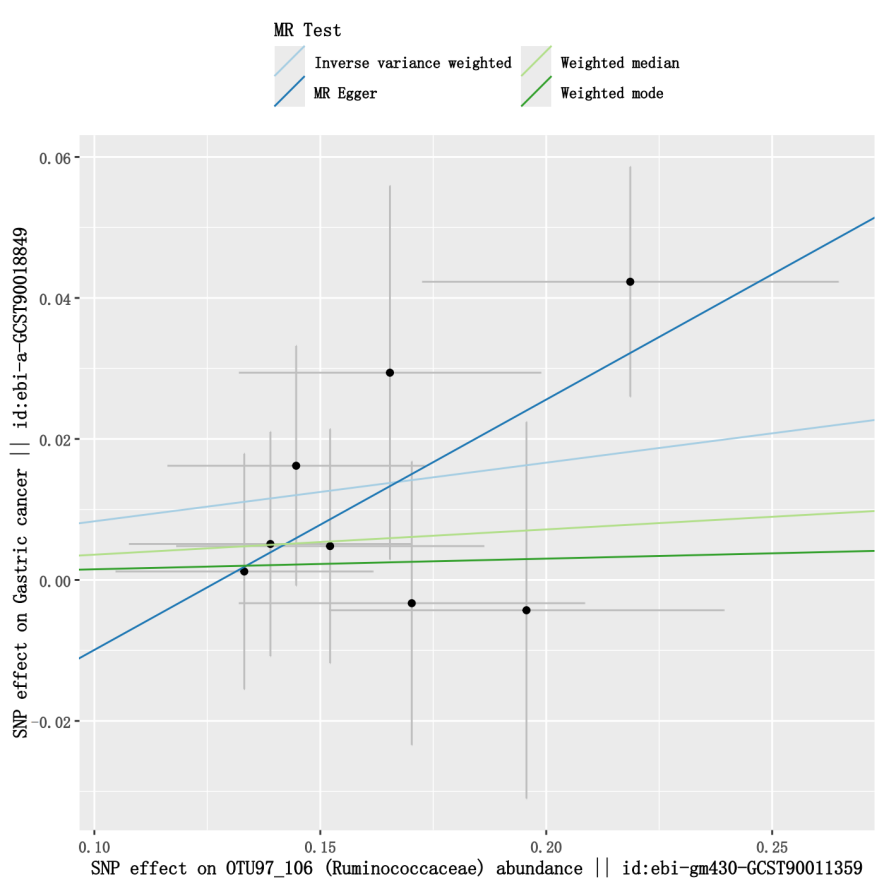

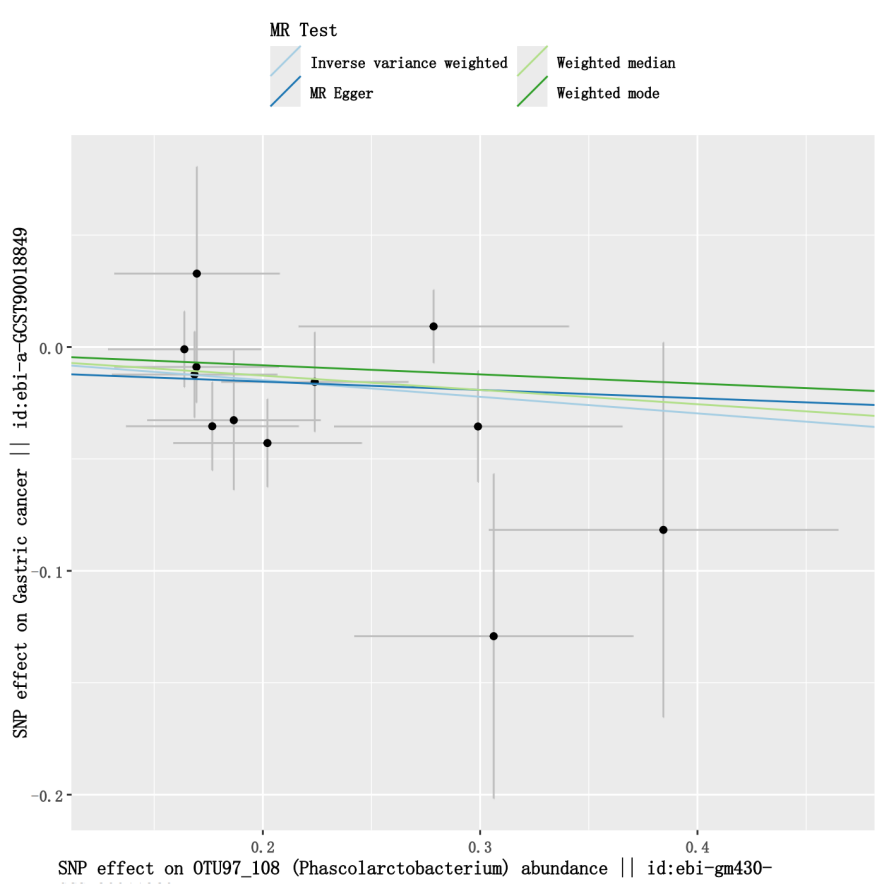

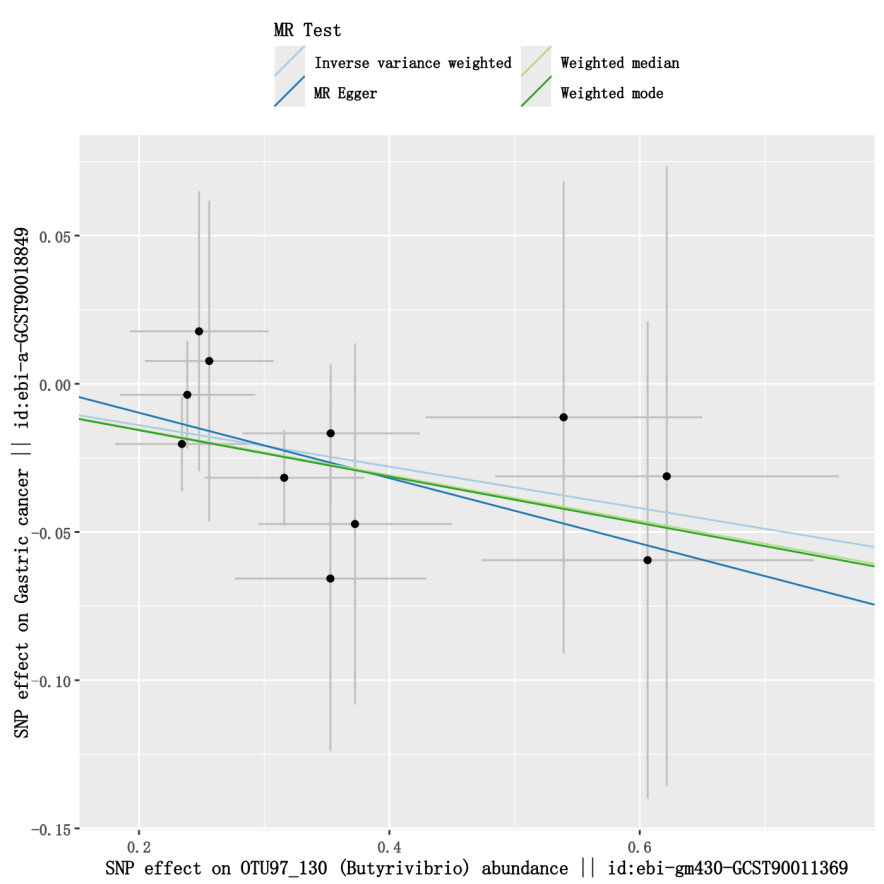


(G) OTU97_108 (*Phascolarctobacterium*) versus GC (H) OTU97_130 (*Butyrivibrio*) versus GC

(I) OTU97_39 (*Proteobacteria*) versus GC (J) OTU97_56 (*Ruminococcaceae*) versus GC


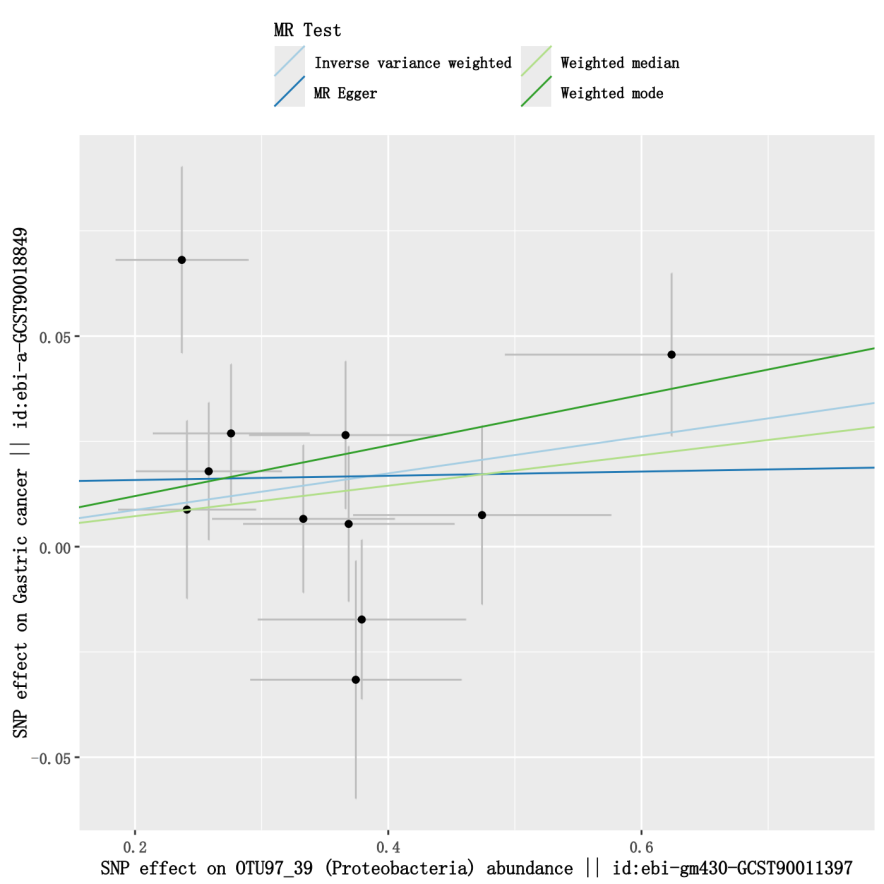

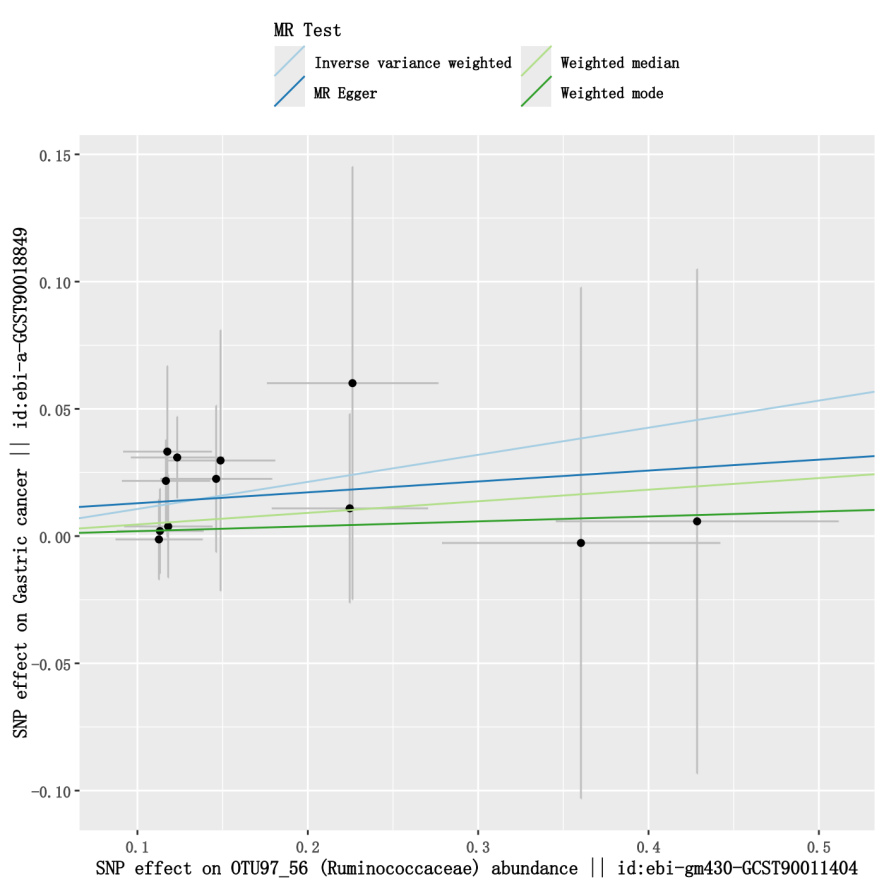

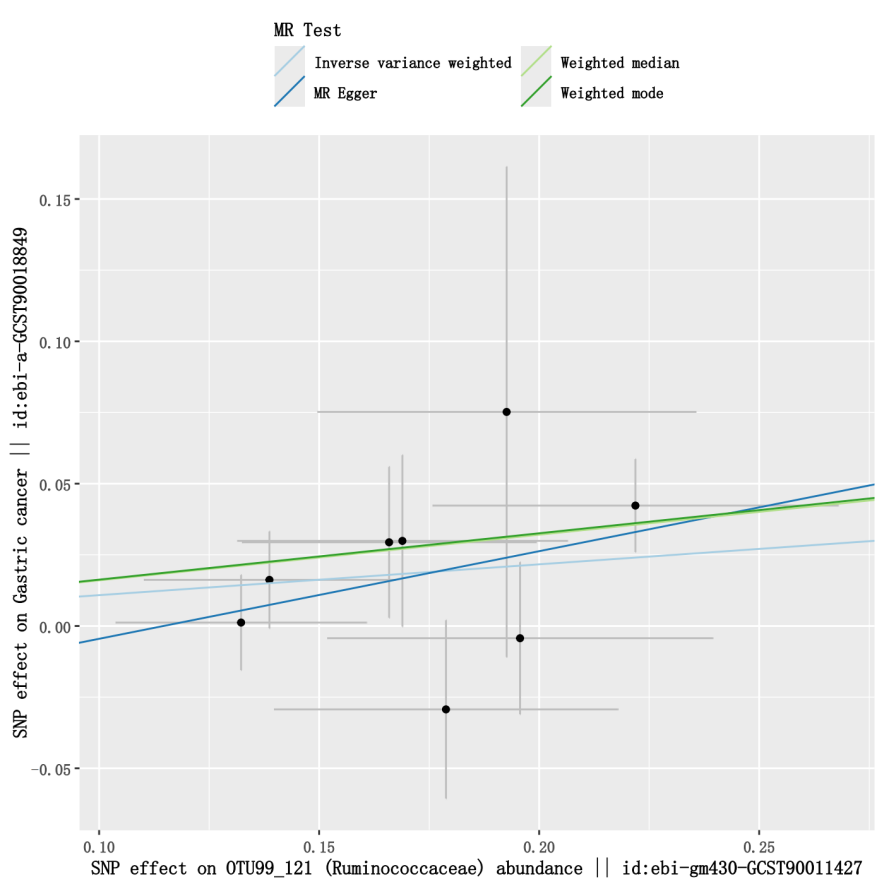

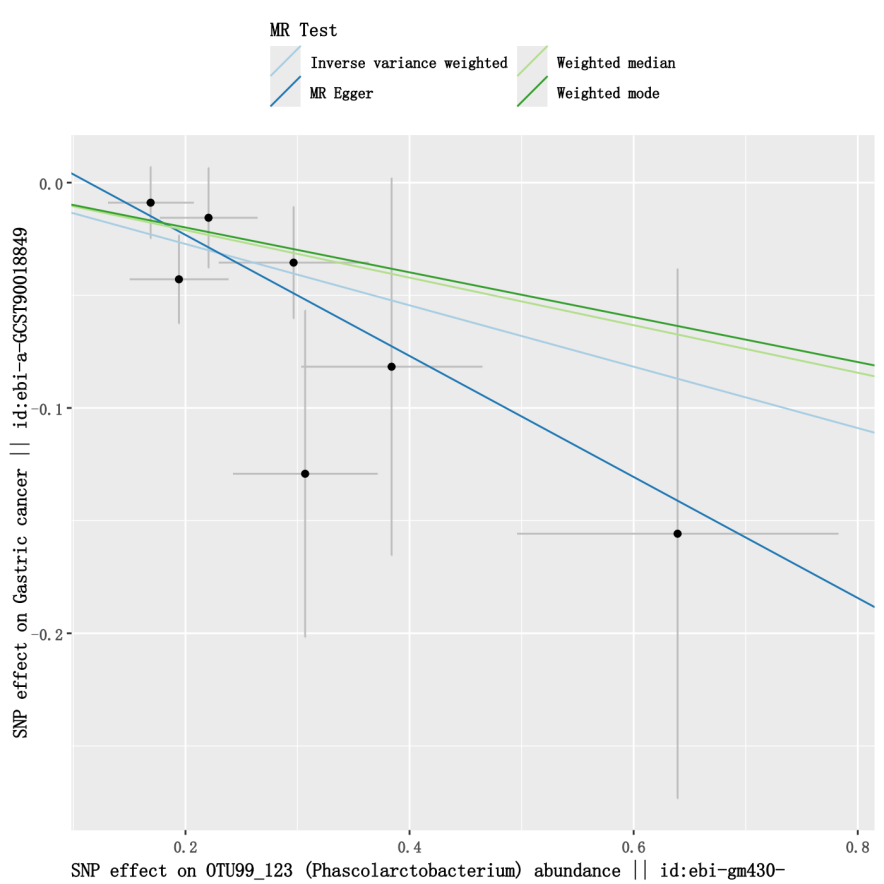


(K) OTU99_121 (*Ruminococcaceae*) versus GC (L) OTU99_123 (*Phascolarctobacterium*) versus GC

(M) OTU99_155 (*Butyrivibrio*) versus GC (N) OTU99_40 (*Proteobacteria*) versus GC


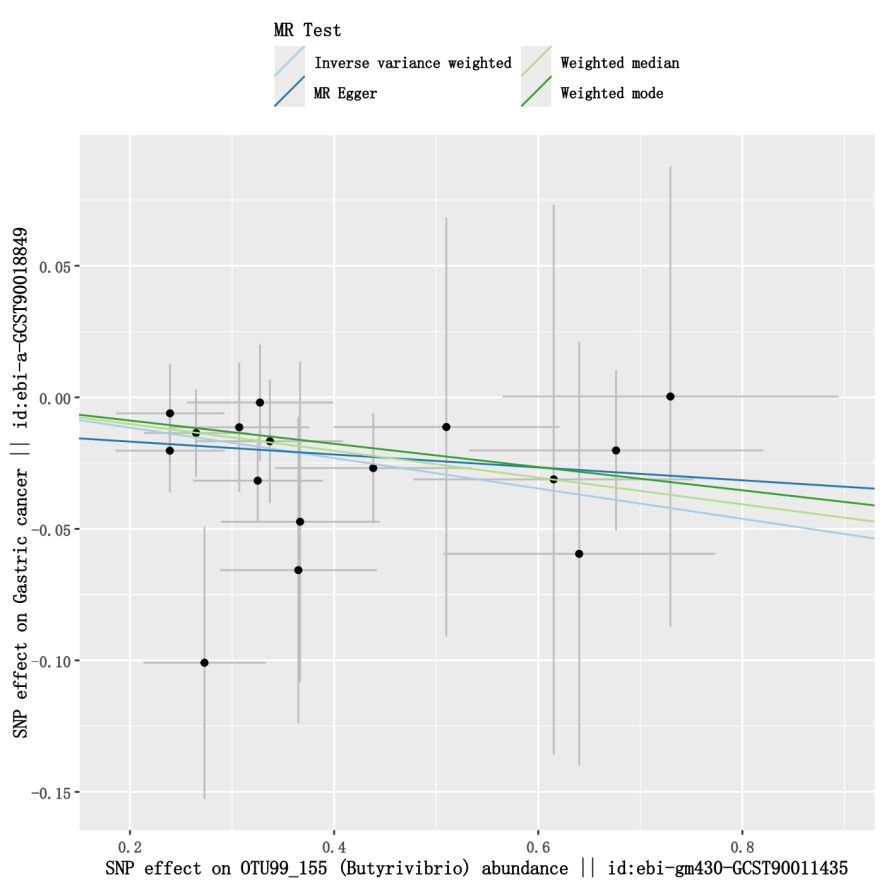

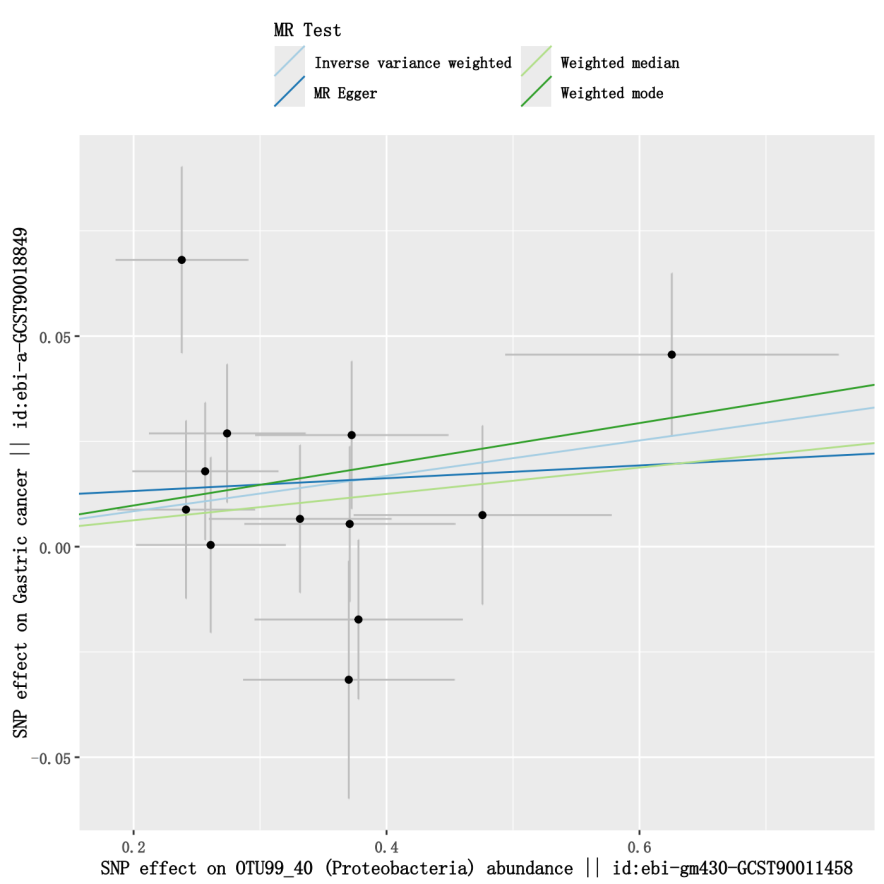

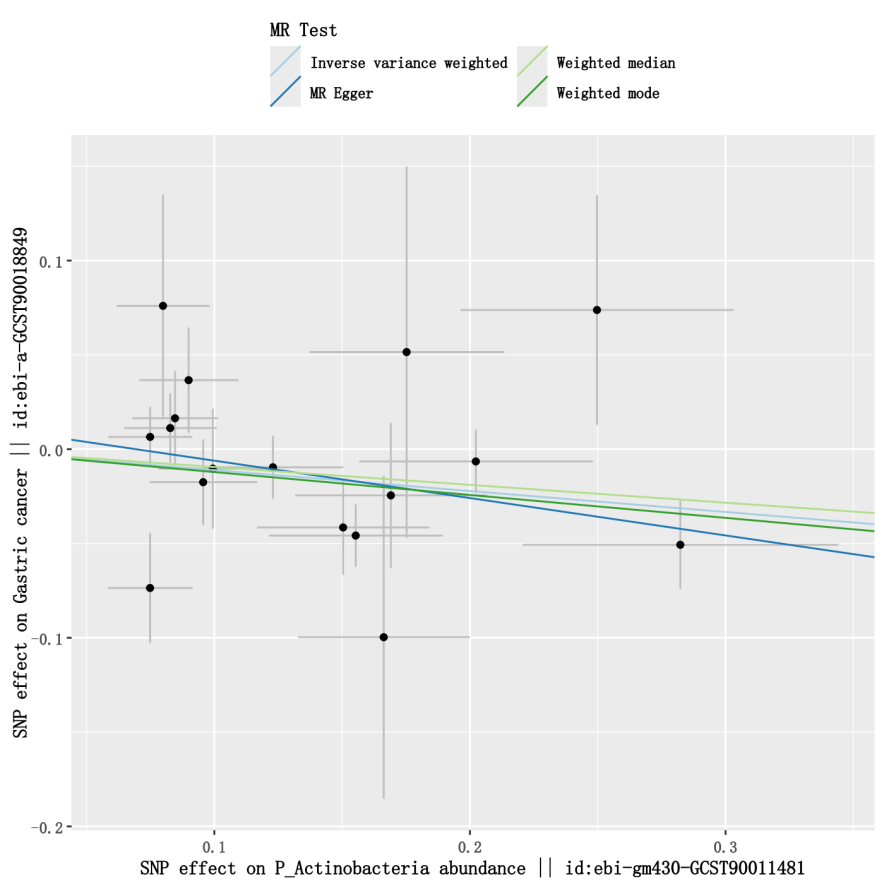

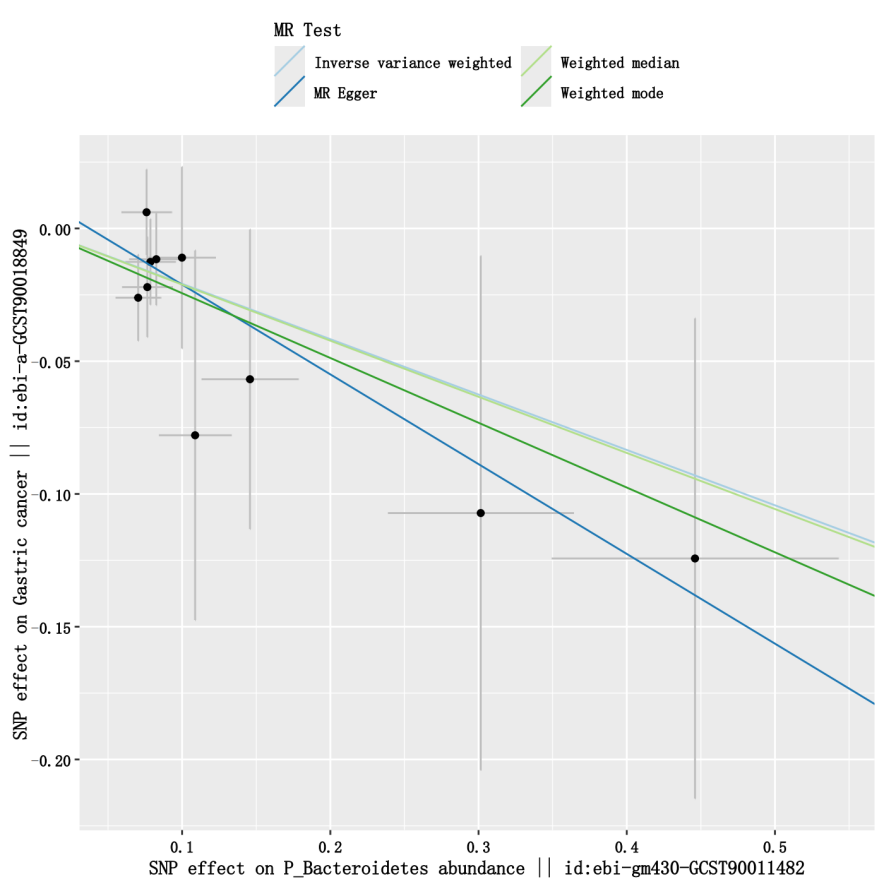


(O) P_*Actinobacteria* versus GC (P) P_*Bacteroidetes* versus GC

(Q) TestASV_3 (*Bacteroides*) versus GC (R) OTU97_137 (*Catenibacterium*) versus GC


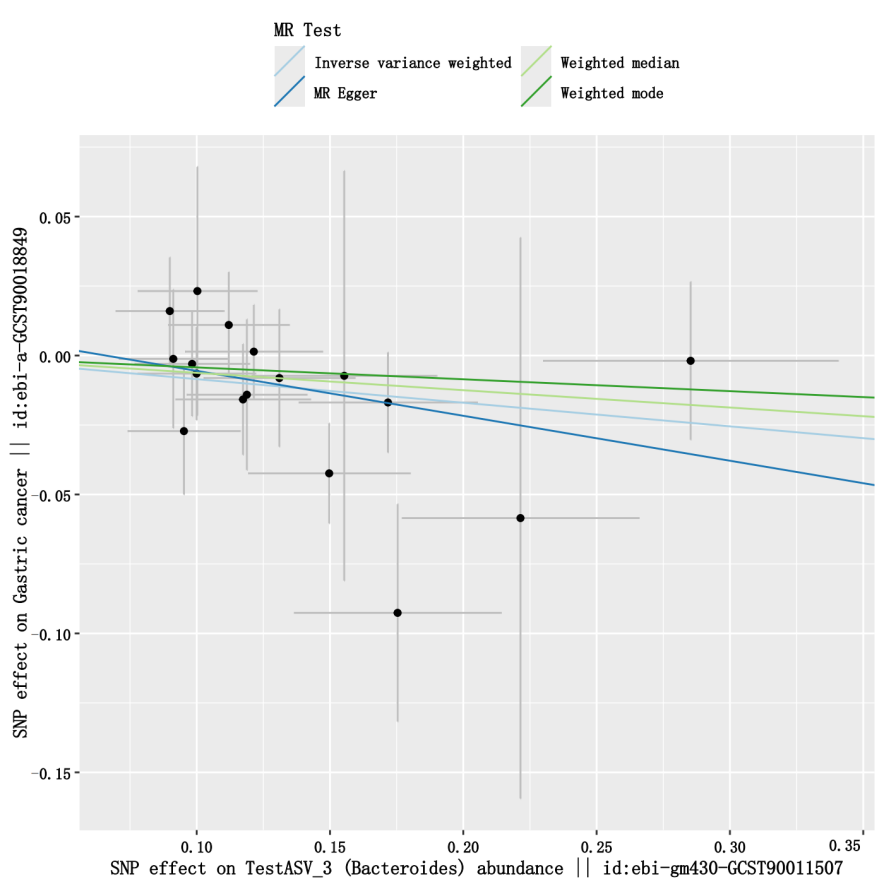

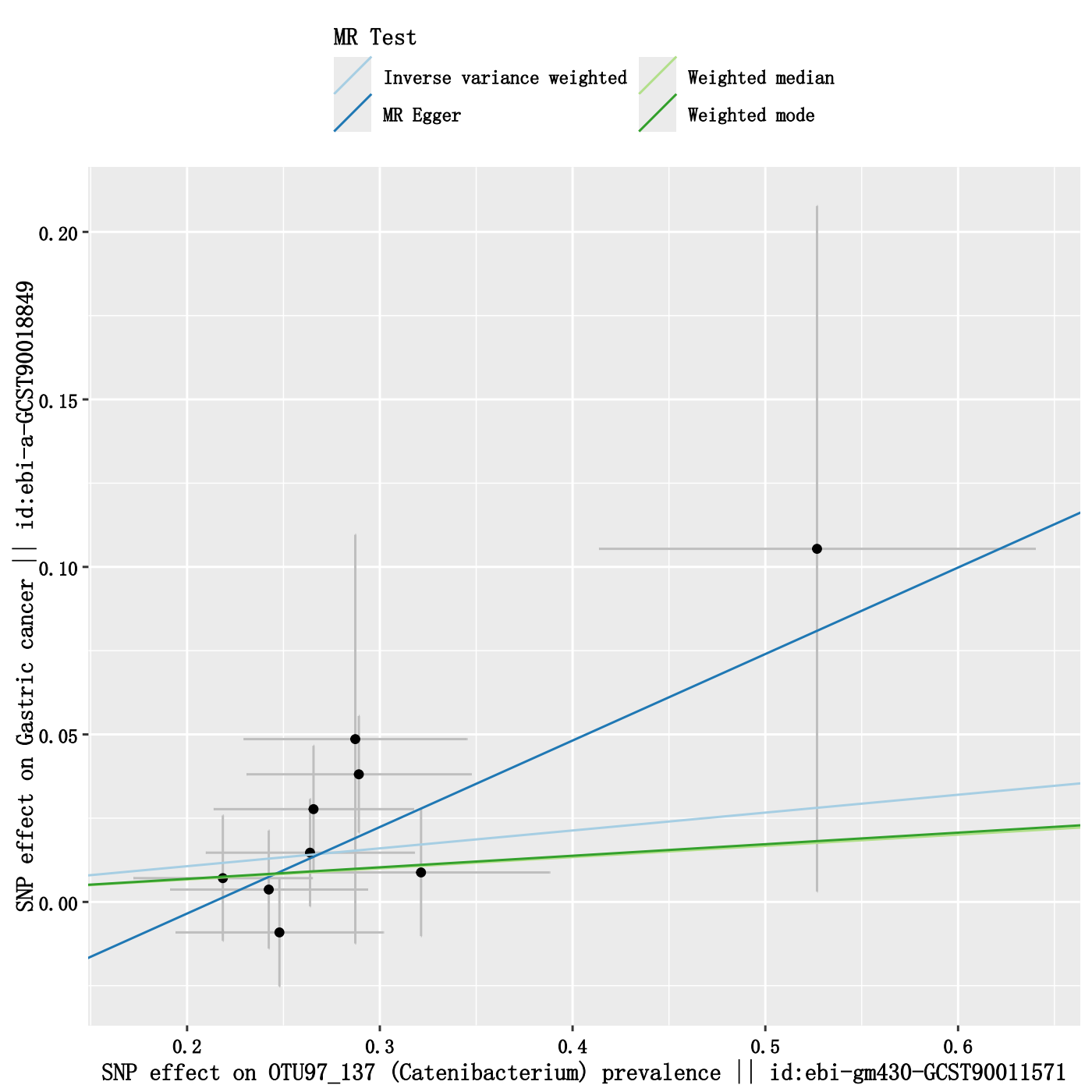

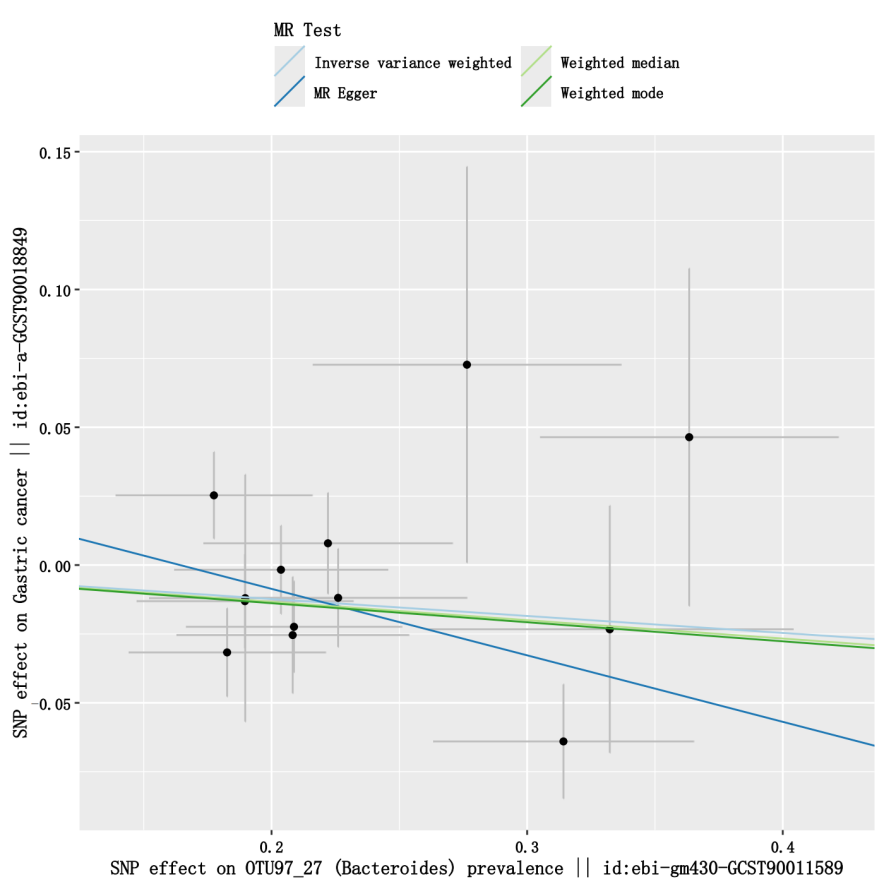


(S) OTU97_27 (*Bacteroides*) versus GC

**Supplementary Figure 2: Scatter plots of SNP effects on gut microbiota versus gastric cancer.** (A) C_*Actinobacteria*; (B) C_*Bacteroidia*; (C) F_*Porphyromonadaceae*; (D) G_*Clostridiales*; (E) O_*Bacteroidales*; (F) OTU97_106 (*Ruminococcaceae*); (G) OTU97_108 (*Phascolarctobacterium*); (H) OTU97_130 (*Butyrivibrio*); (I) OTU97_39 (*Proteobacteria*); (J) OTU97_56 (*Ruminococcaceae*); (K) OTU99_121 (*Ruminococcaceae*); (L) OTU99_123 (*Phascolarctobacterium*); (M) OTU99_155 (*Butyrivibrio*); (N) OTU99_40 (*Proteobacteria*); (O) P_*Actinobacteria*; (P) P_*Bacteroidetes*; (Q) TestASV_3 (*Bacteroides*); (R) OTU97_137 (*Catenibacterium*); (S) OTU97_27 (*Bacteroides*).

1. Phospholipids in small LDL versus GC (B) Phospholipids in medium LDL versus GC


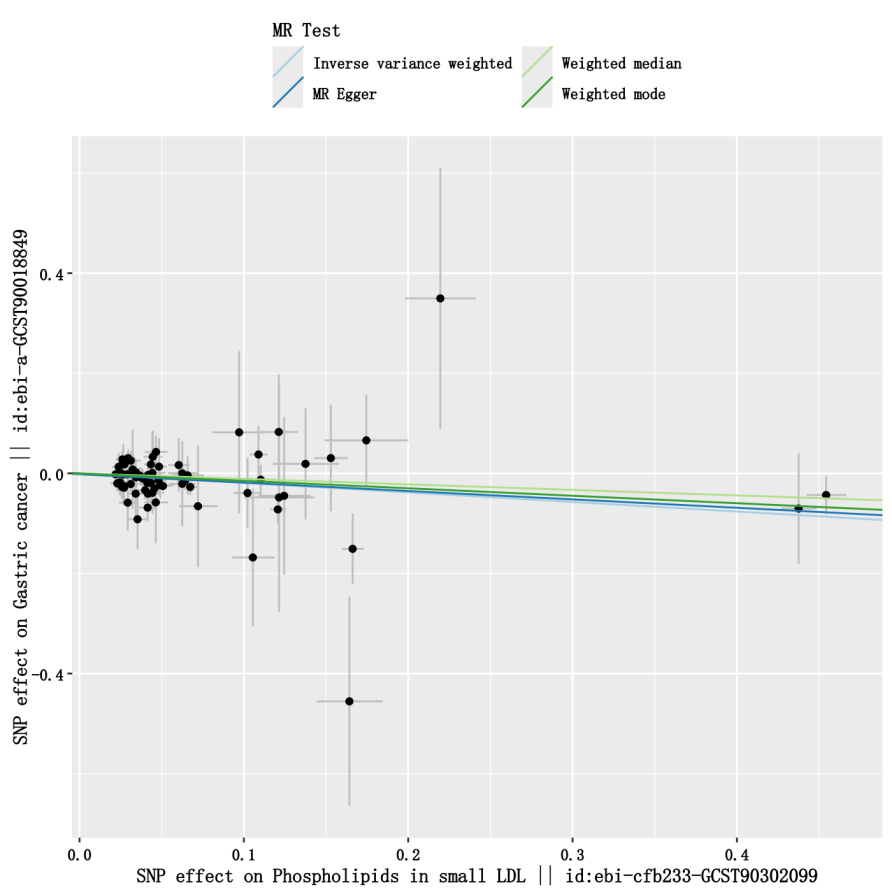

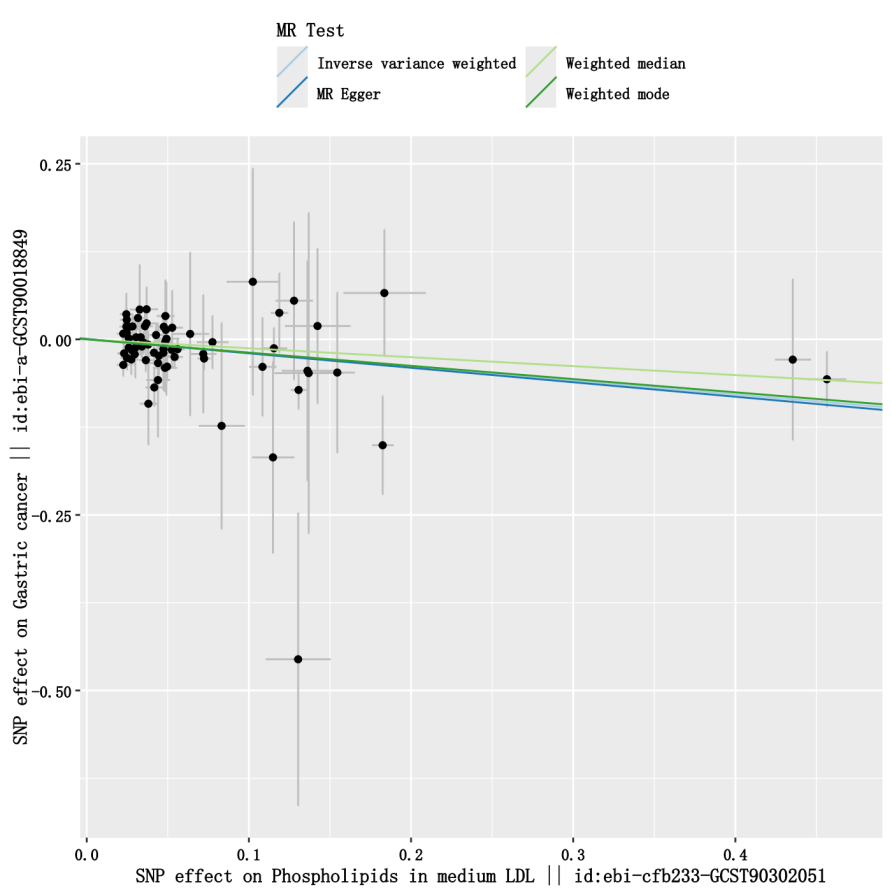

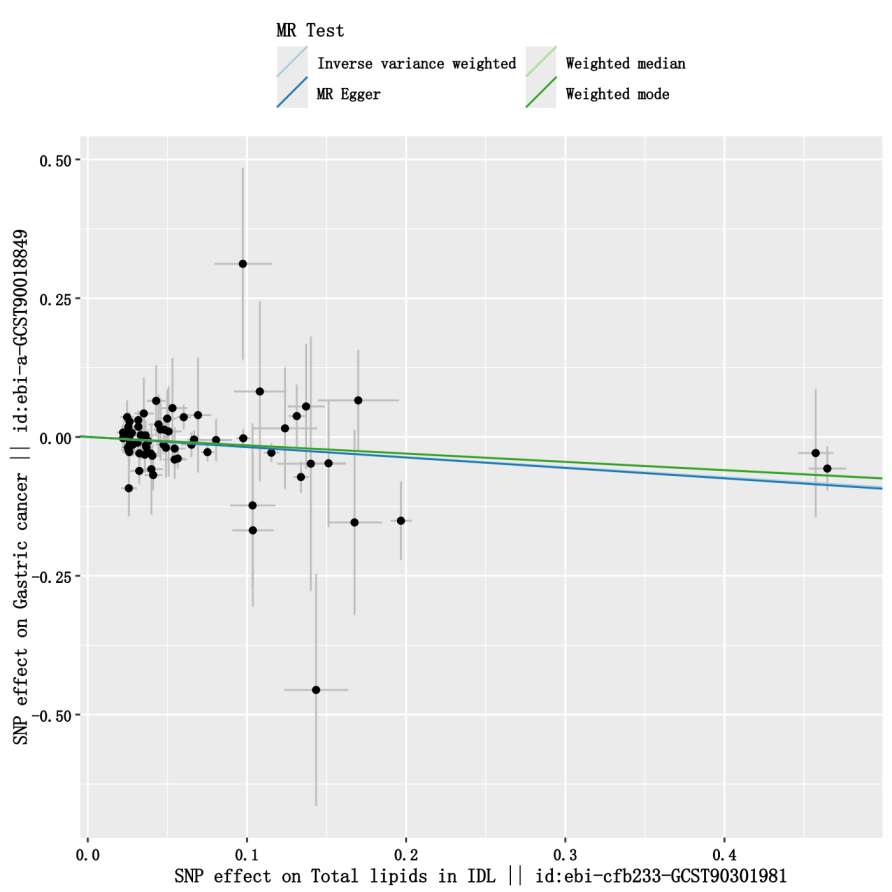

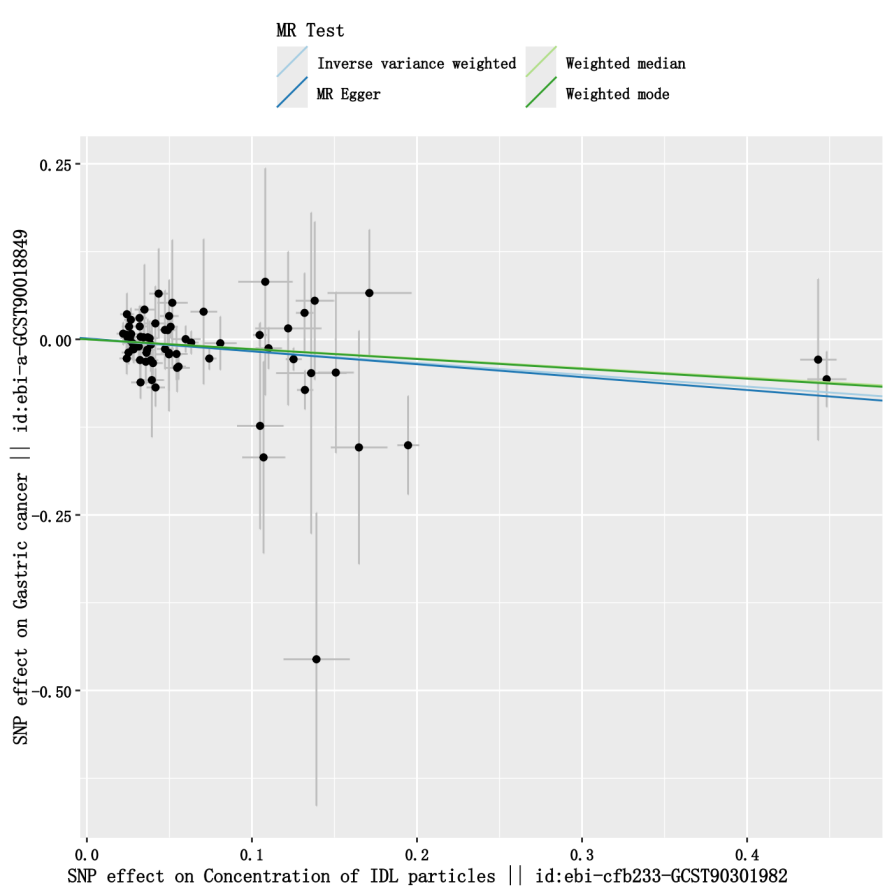


(C) Total lipids in IDL versus GC (D) Concentration of IDL particles versus GC

(E) Free cholesterol in small LDL versus GC (F) Free cholesterol in medium LDL versus GC


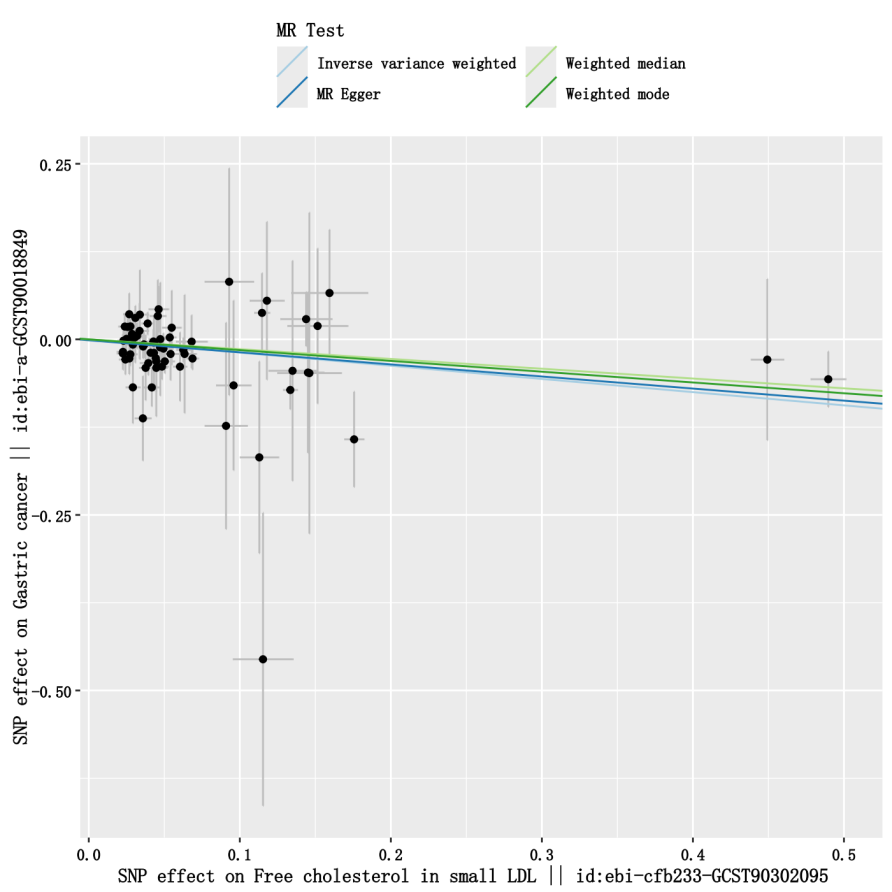

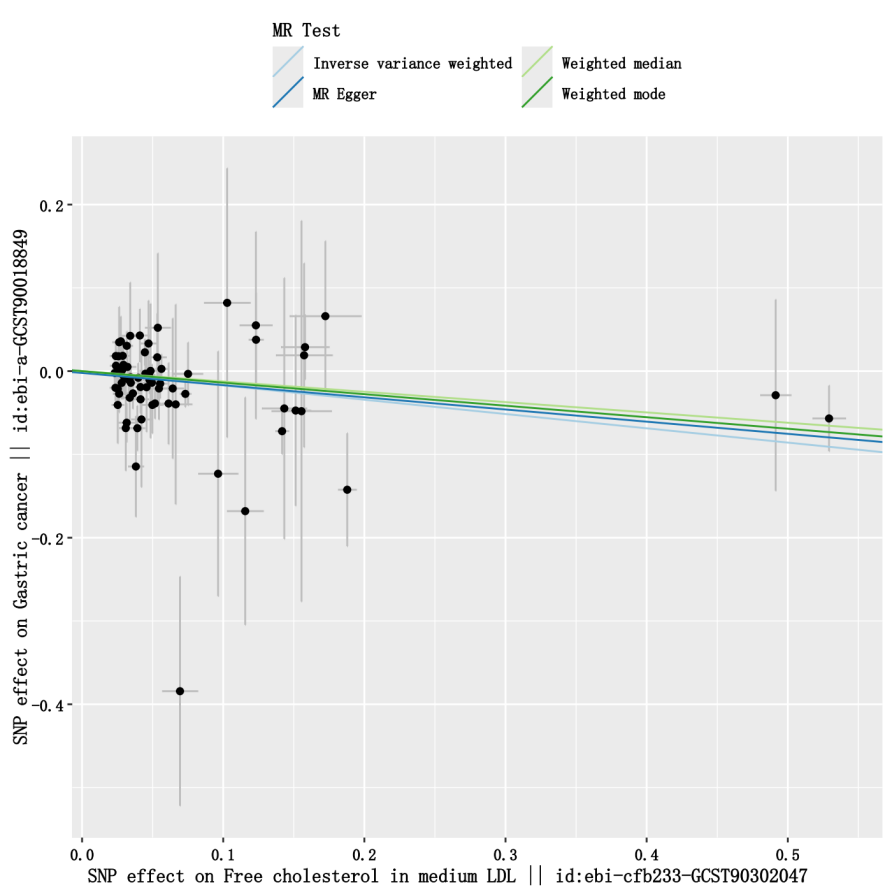

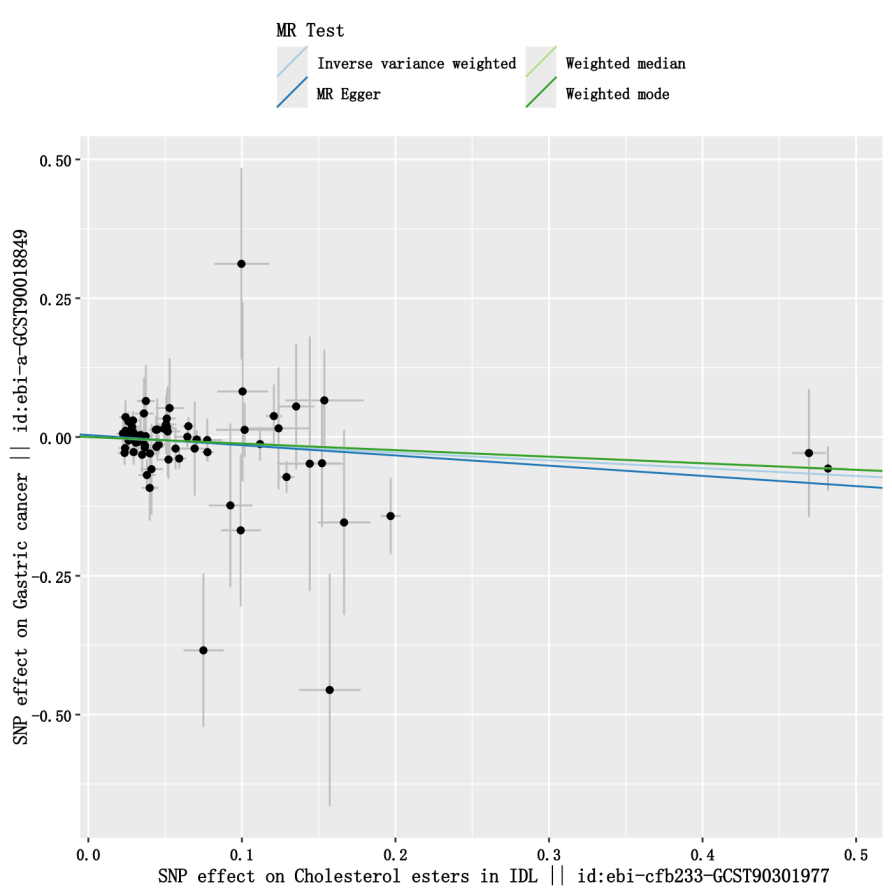


1. Cholesterol esters in IDL versus GC

**Supplementary Figure 3: Scatter plots of SNP effects on blood metabolites versus gastric cancer.** (A) Phospholipids in small LDL (low-density lipoprotein); (B) Phospholipids in medium LDL; (C) Total lipids in IDL (intermediate-density lipoprotein); (D) Concentration of IDL particles; (E) Free cholesterol in small LDL; (F) Free cholesterol in medium LDL; (G) Cholesterol esters in IDL.

(A) (B)


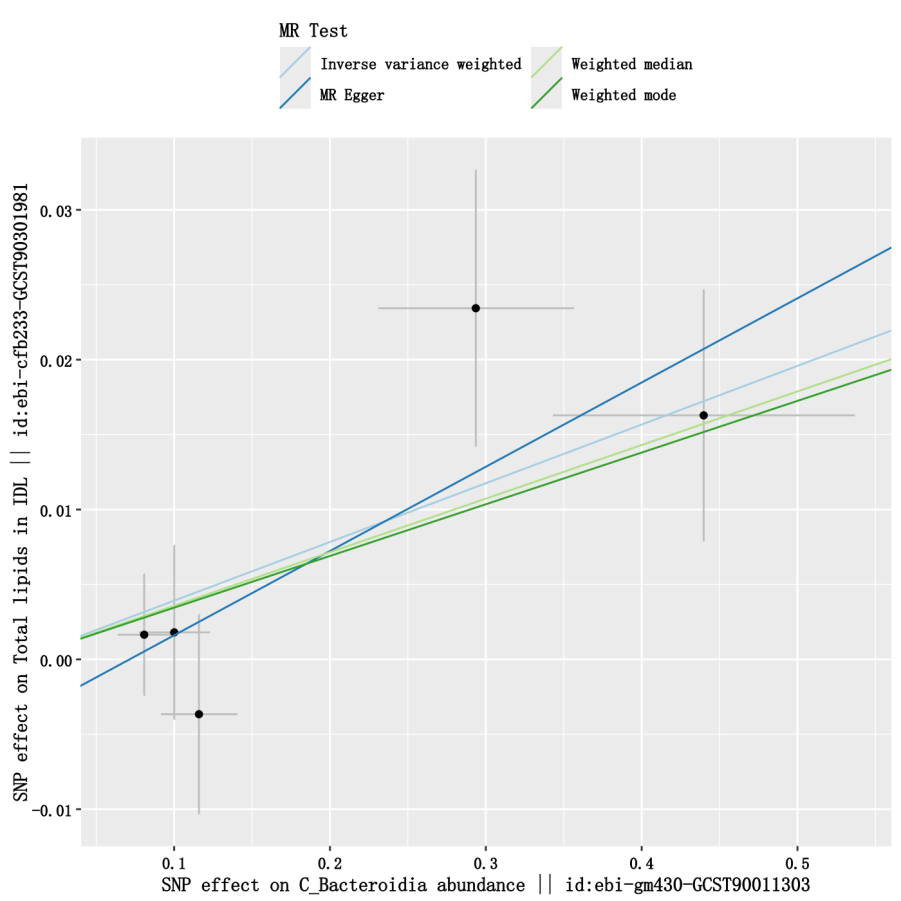

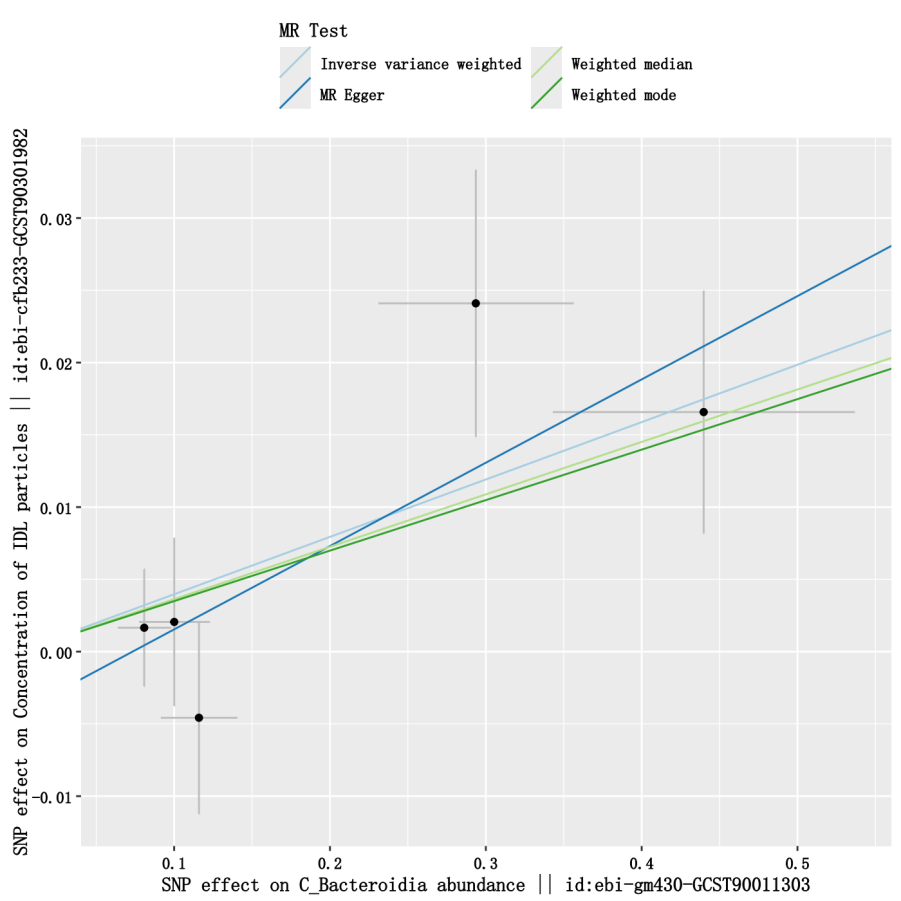

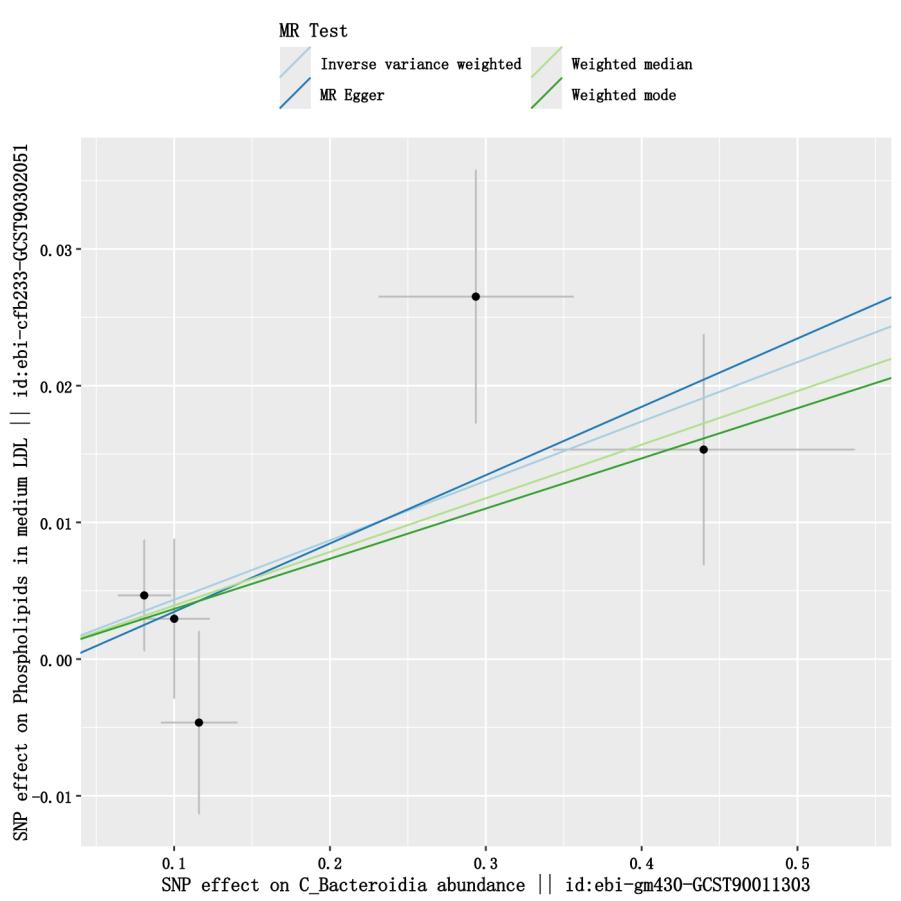

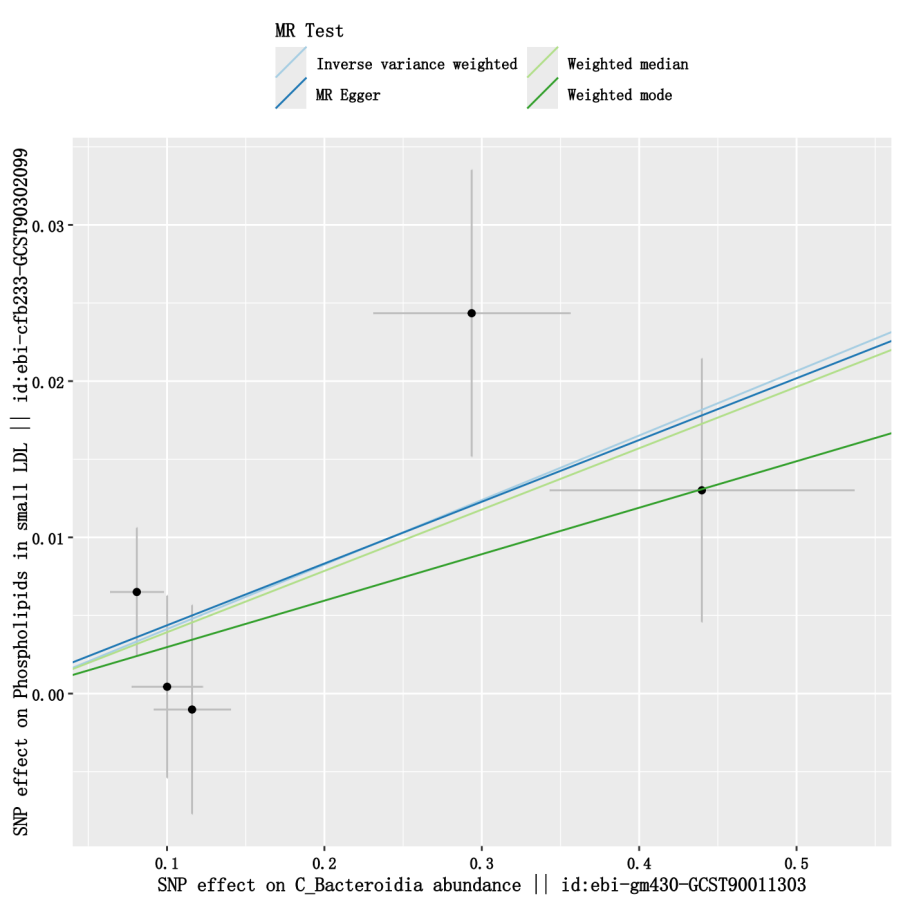


(C) (D)

(E) (F)


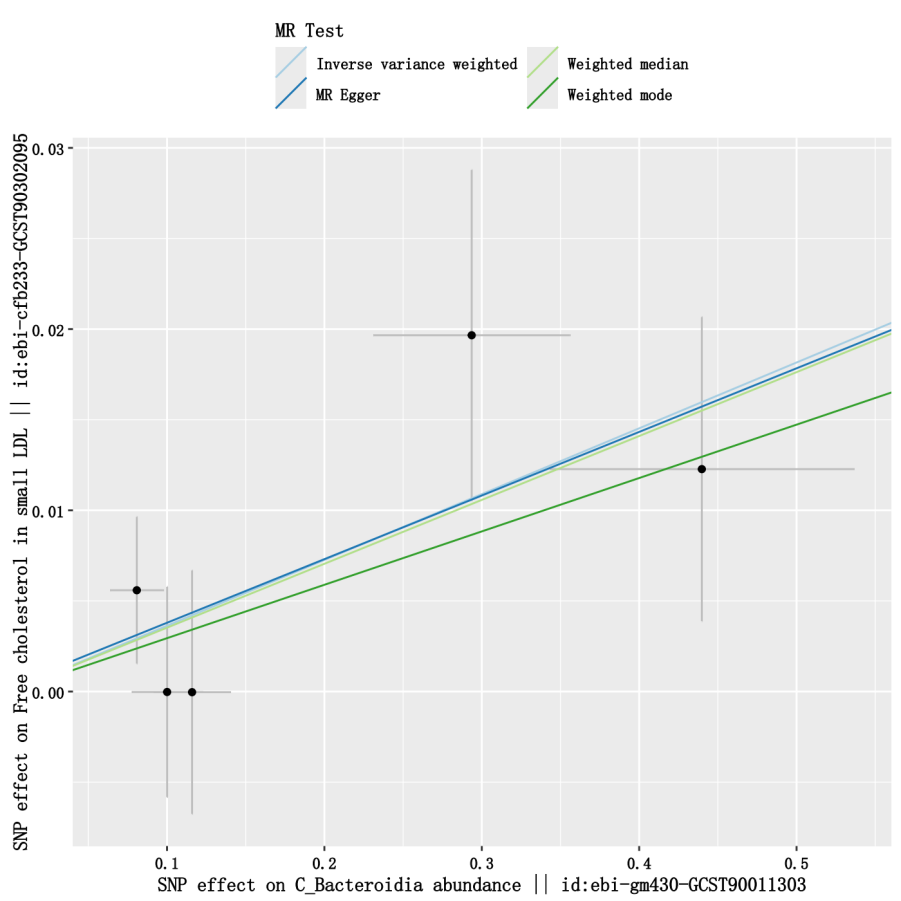

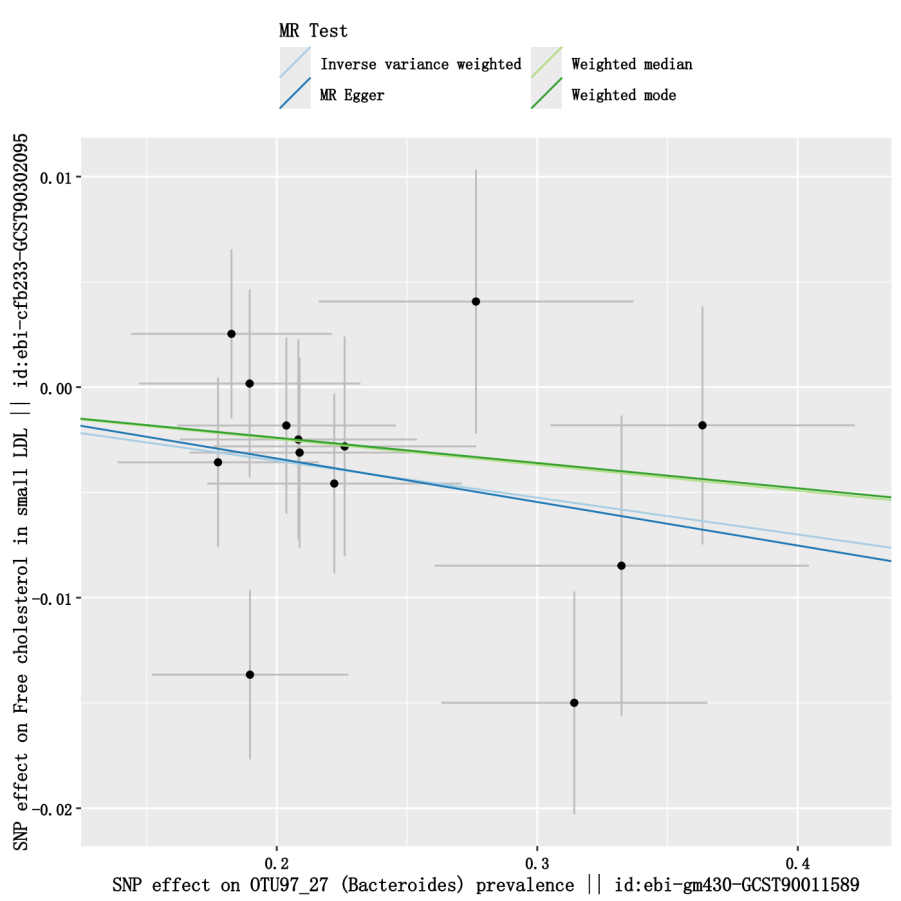


**Supplementary Figure 4: Scatter plots of SNP effects on gut microbiota versus blood metabolites.** (A) C_*Bacteroidia* abundance versus (A) Total lipids in IDL, (B) Concentration of IDL particles, (C) Phospholipids in medium LDL, (D) Phospholipids in small LDL, and (E) Free cholesterol in small LDL; G_OTU97_27 (*Bacteroides*) versus (F) Free cholesterol in small LDL.

(A) (B)


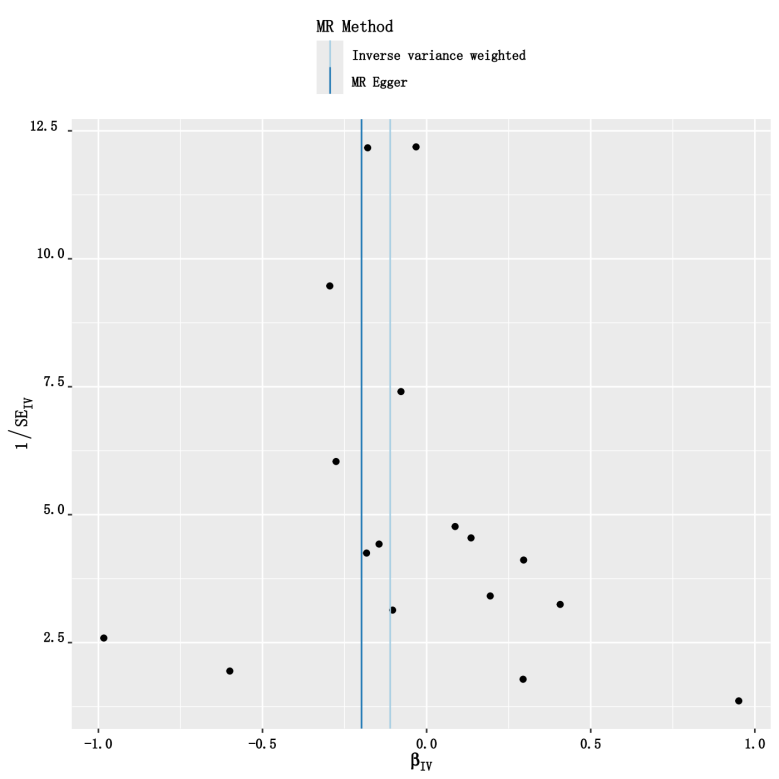

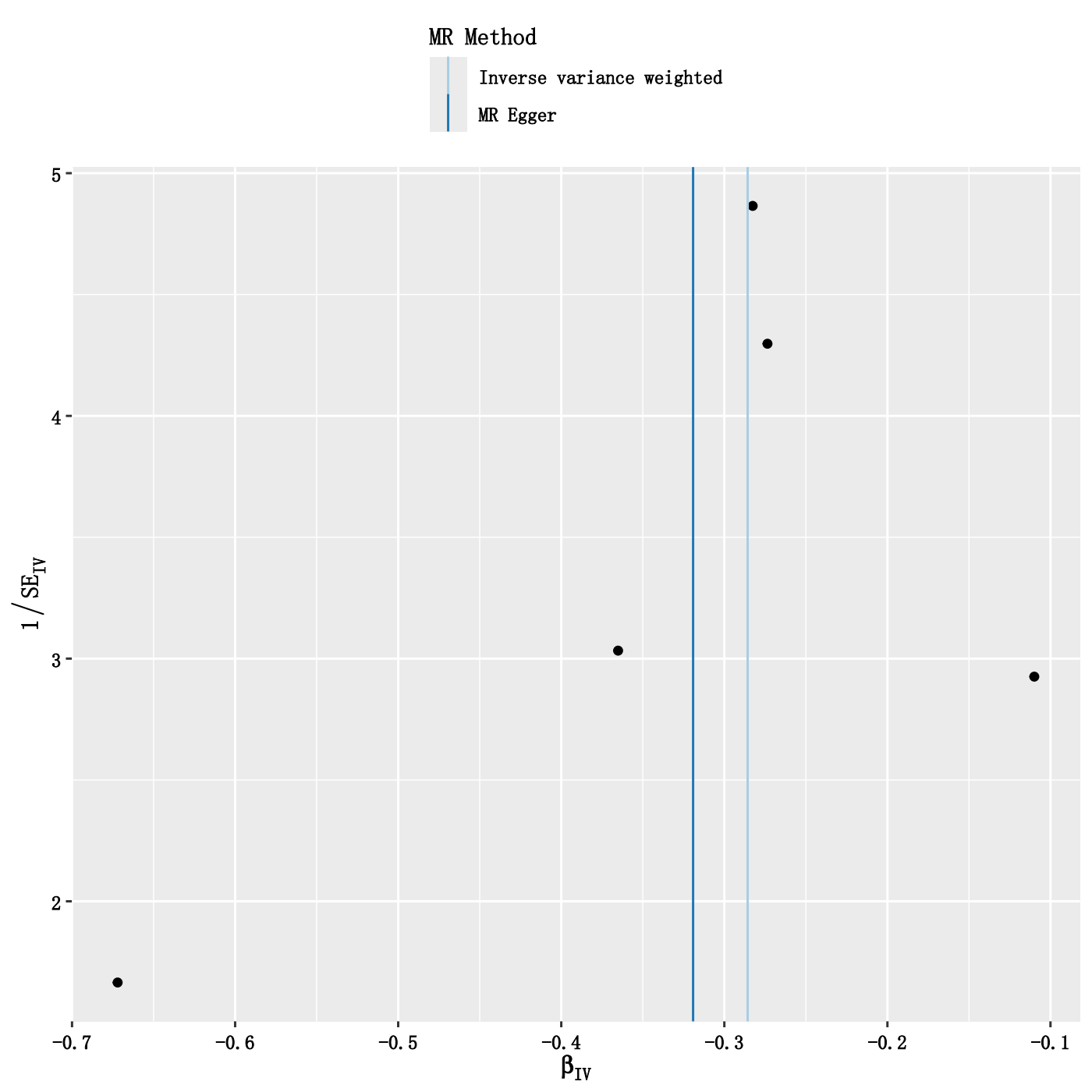

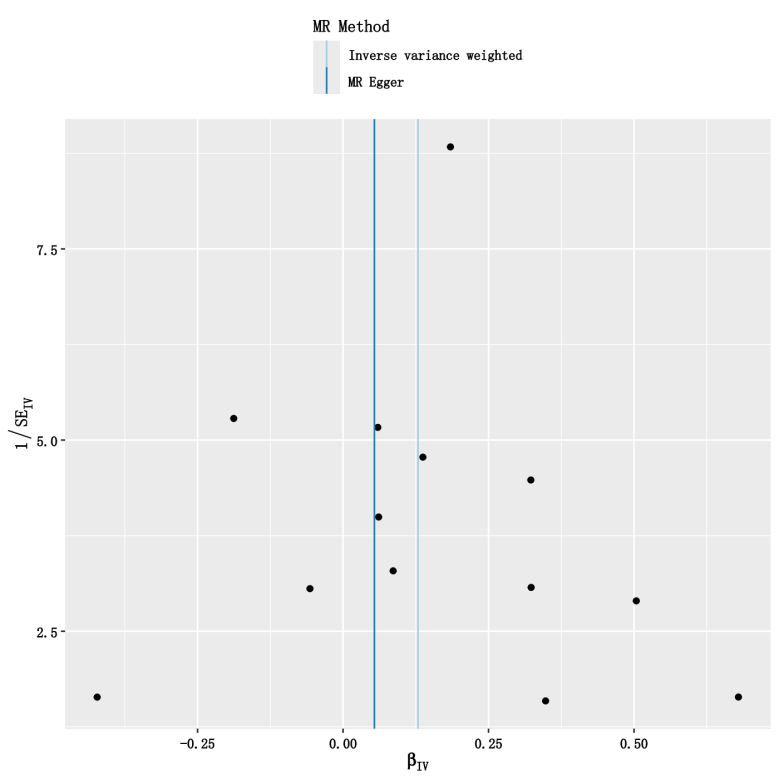

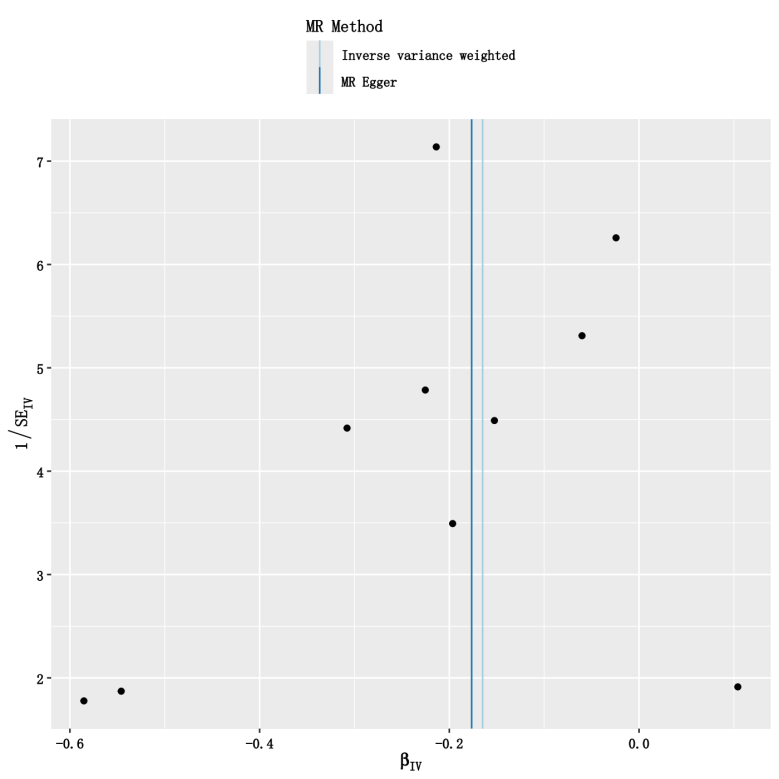

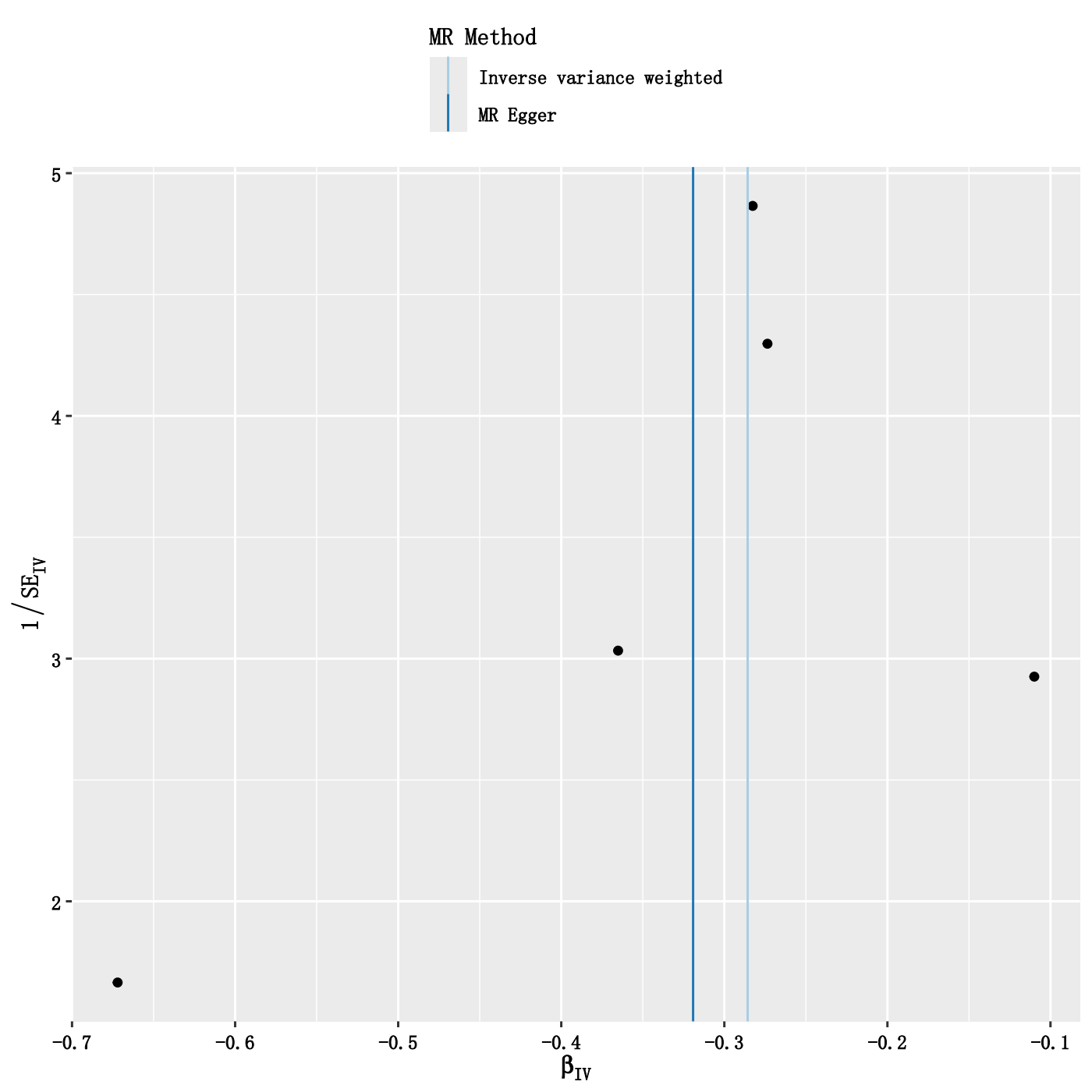

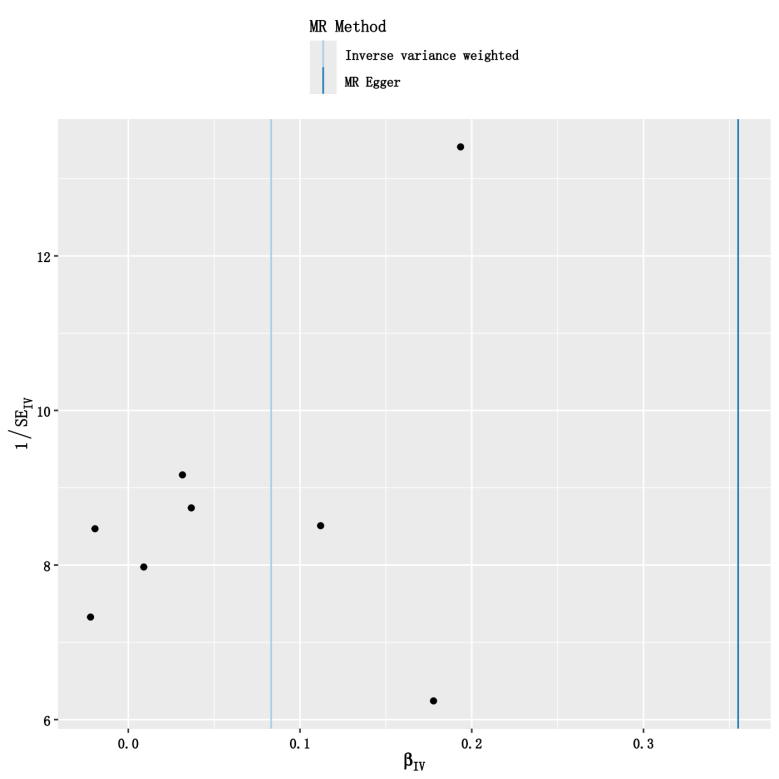


(C) (D)

(E) (F)

(G) (H)


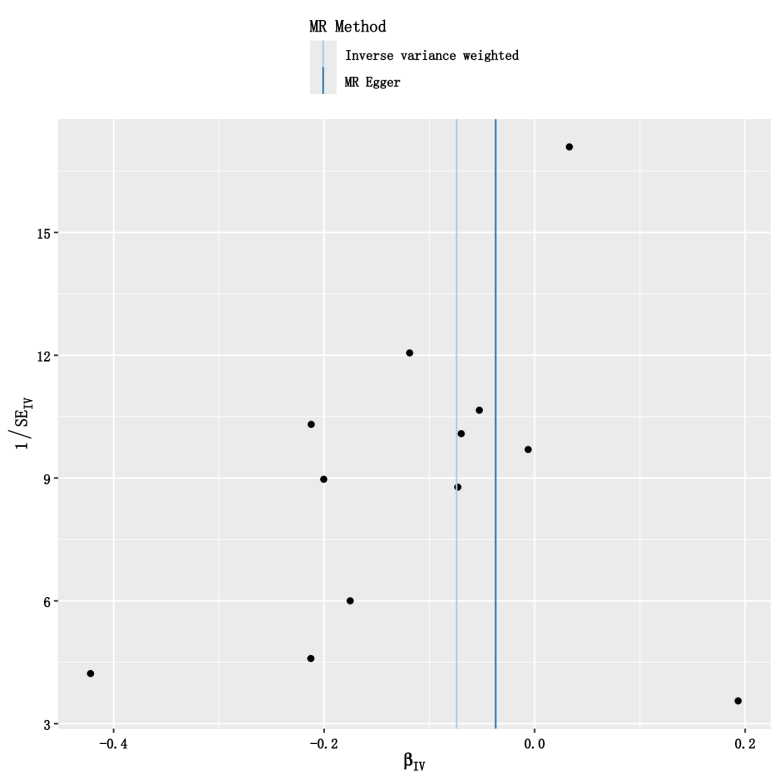

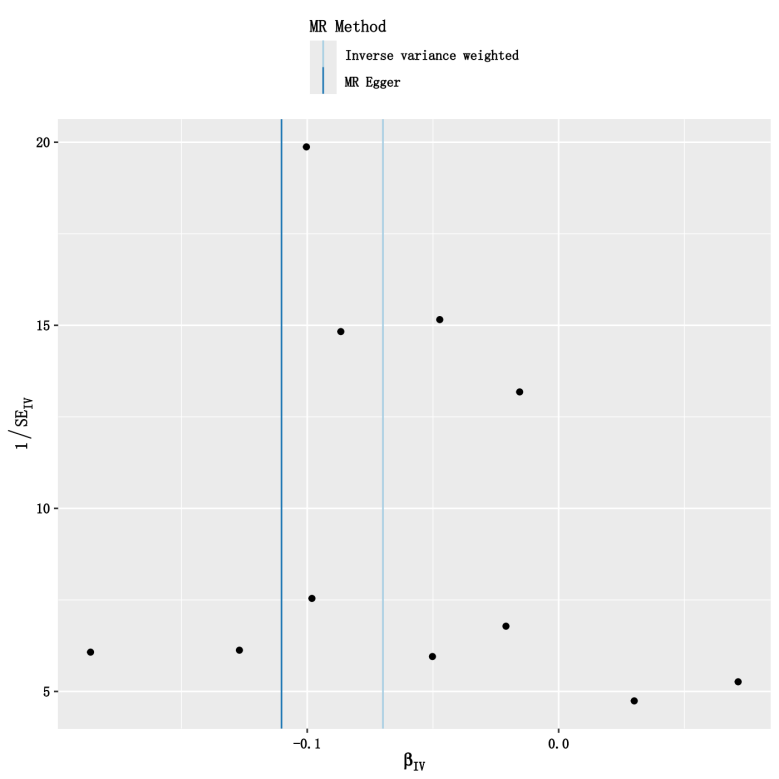

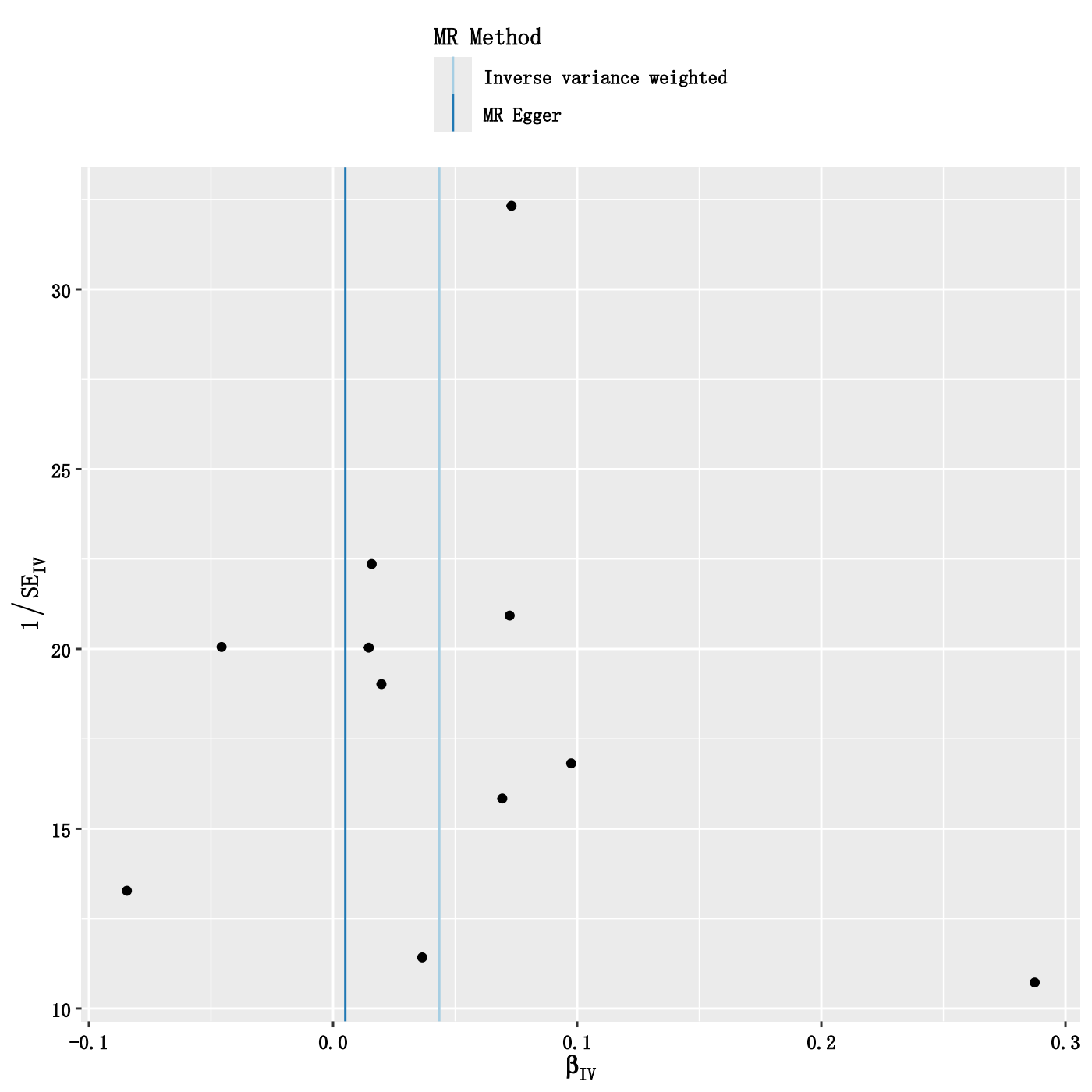

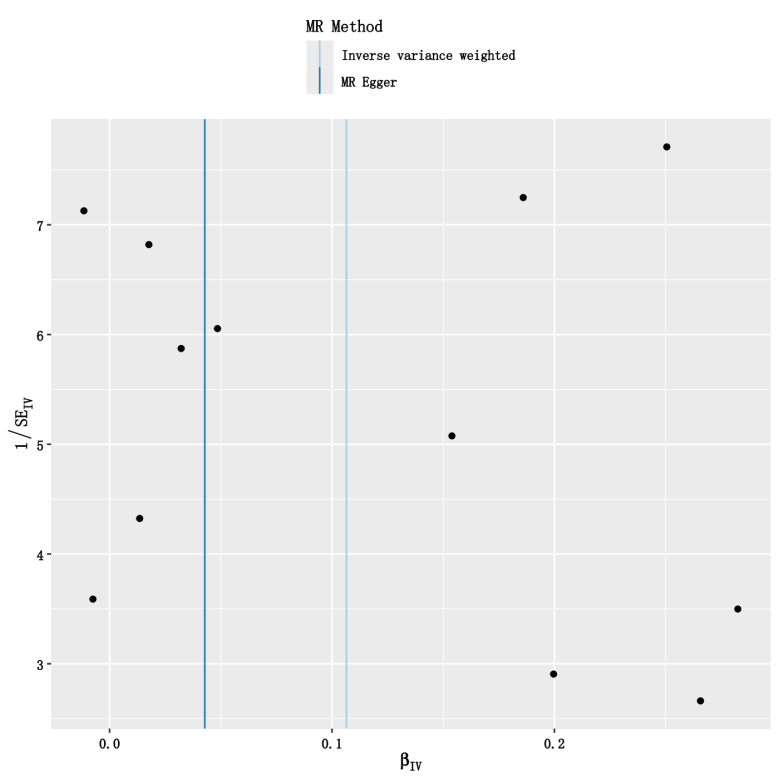

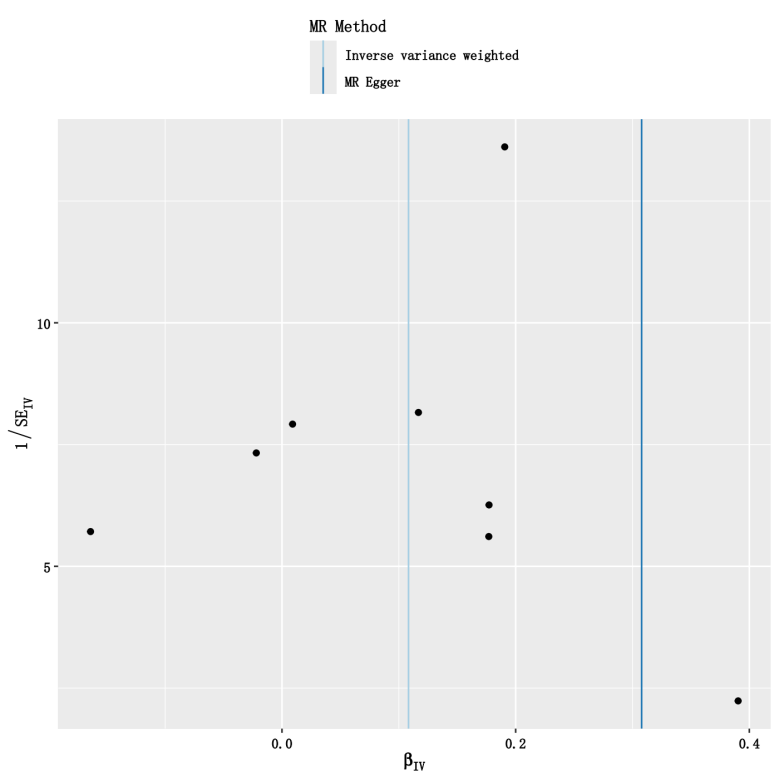

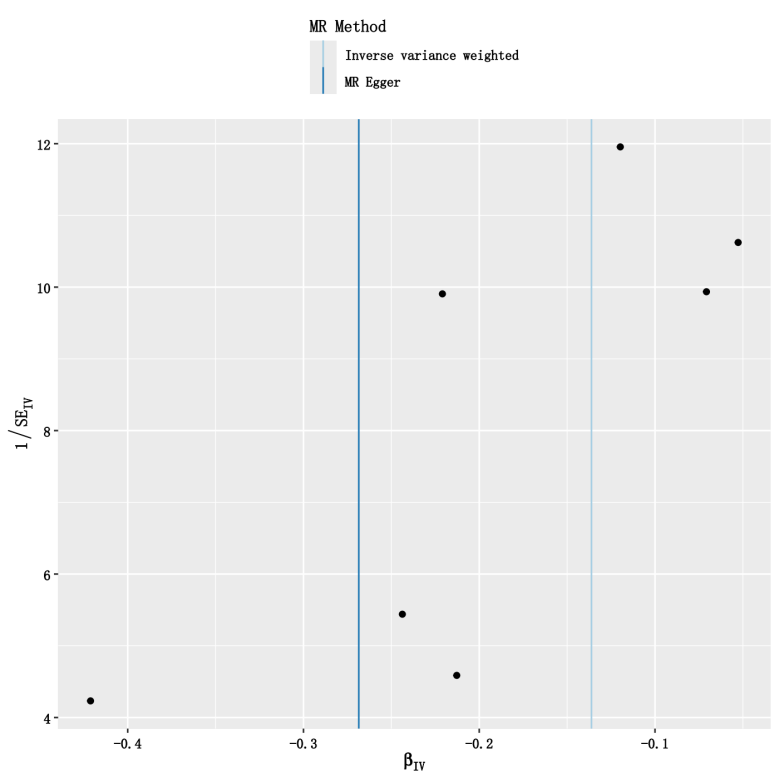


1. (J)

(K) (L)

(M) (N)


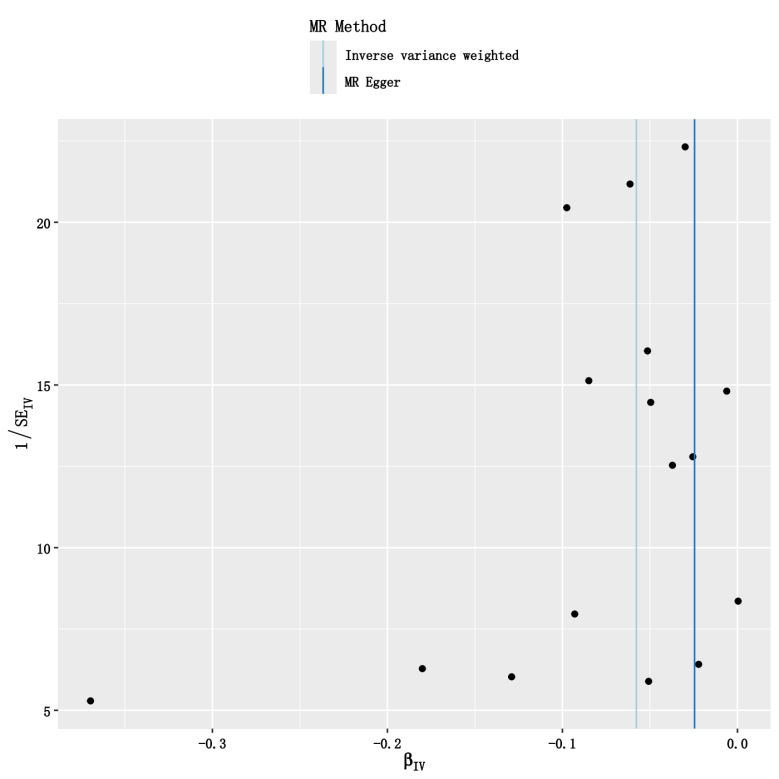

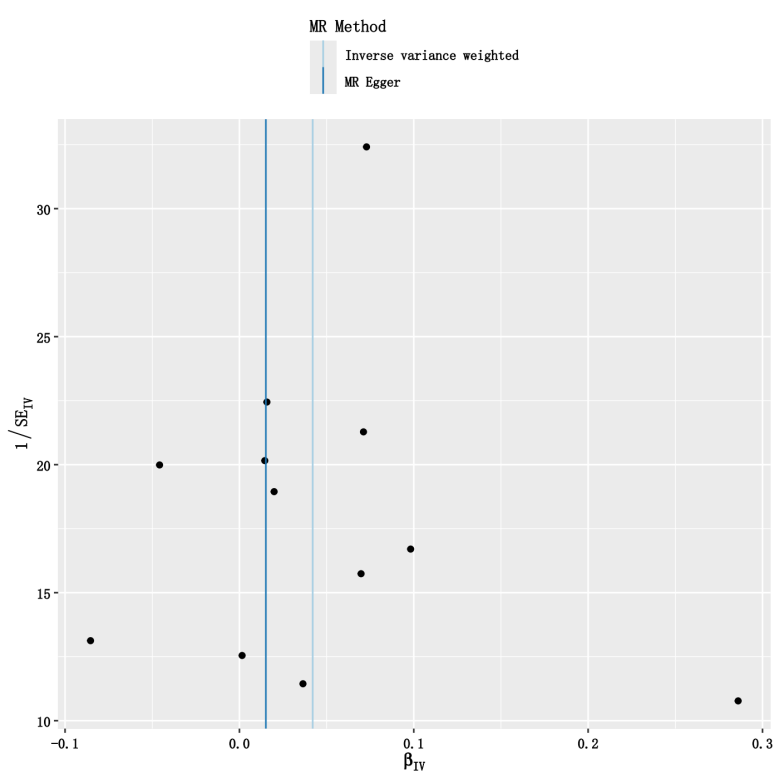

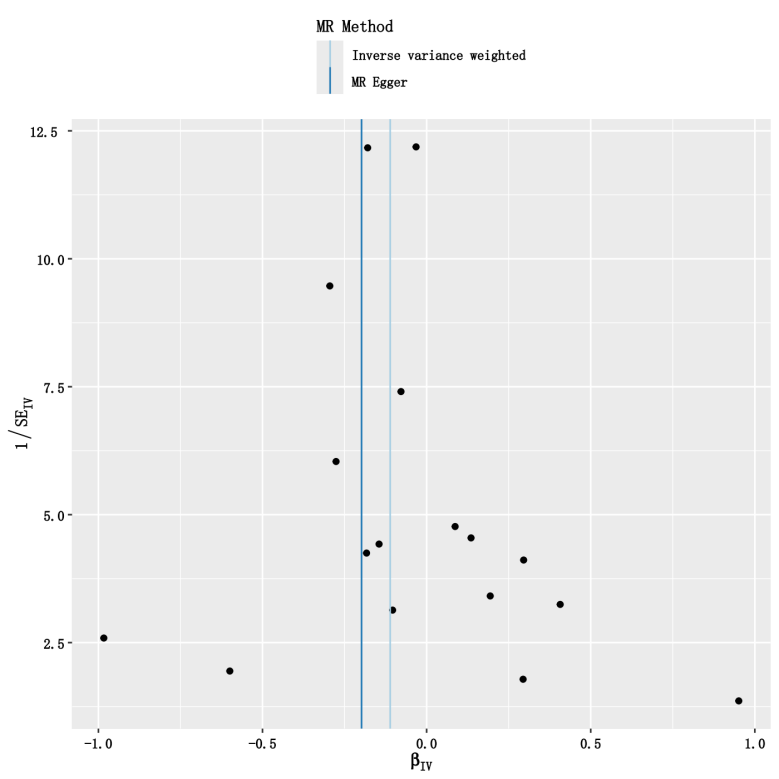

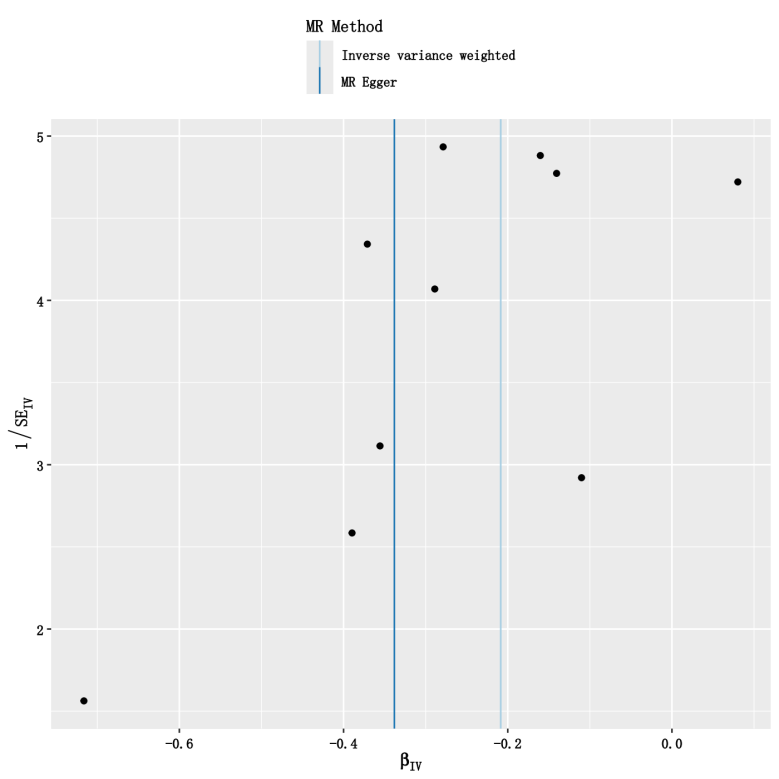

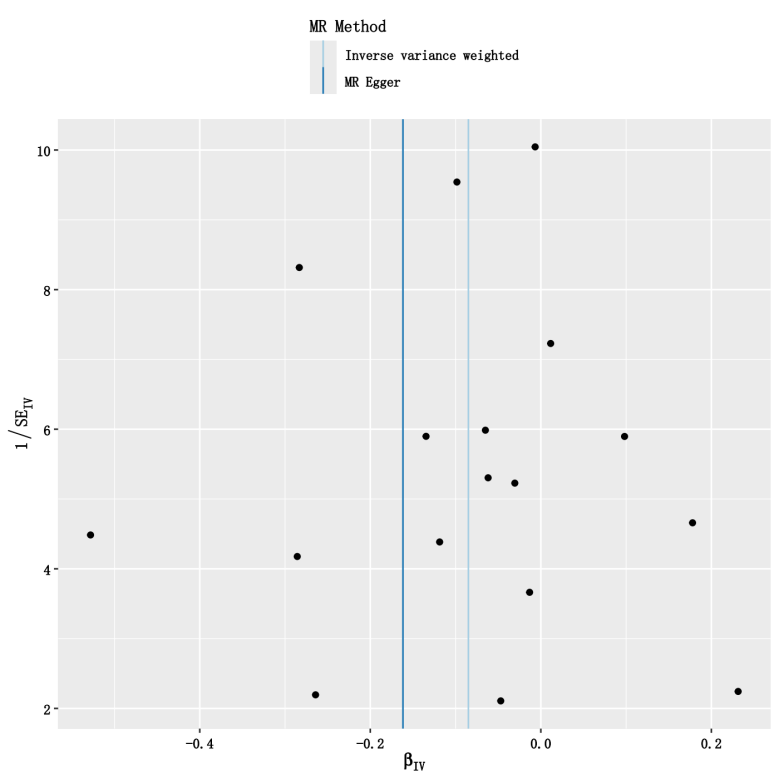

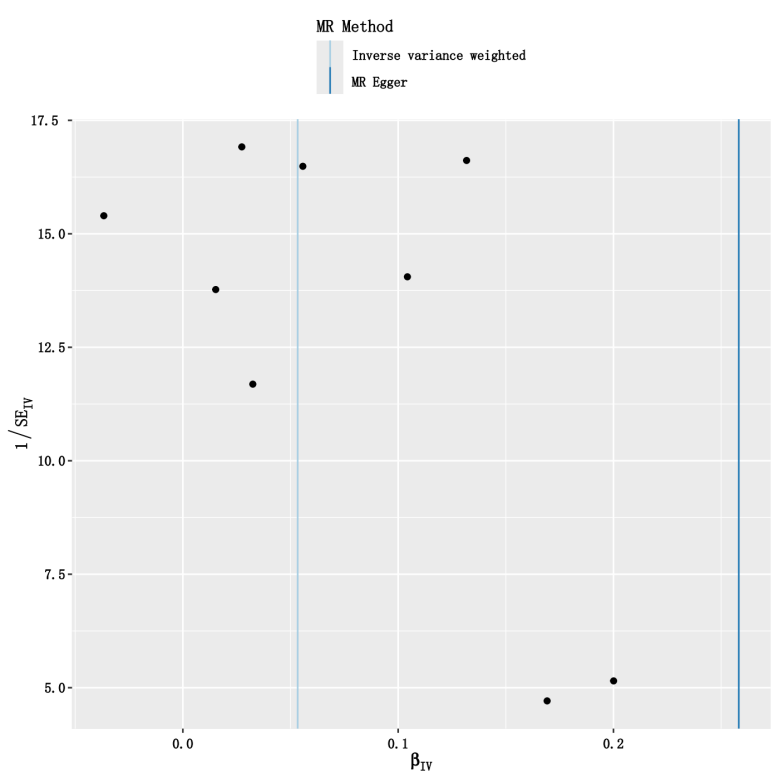


(O) (P)

(Q) (R)


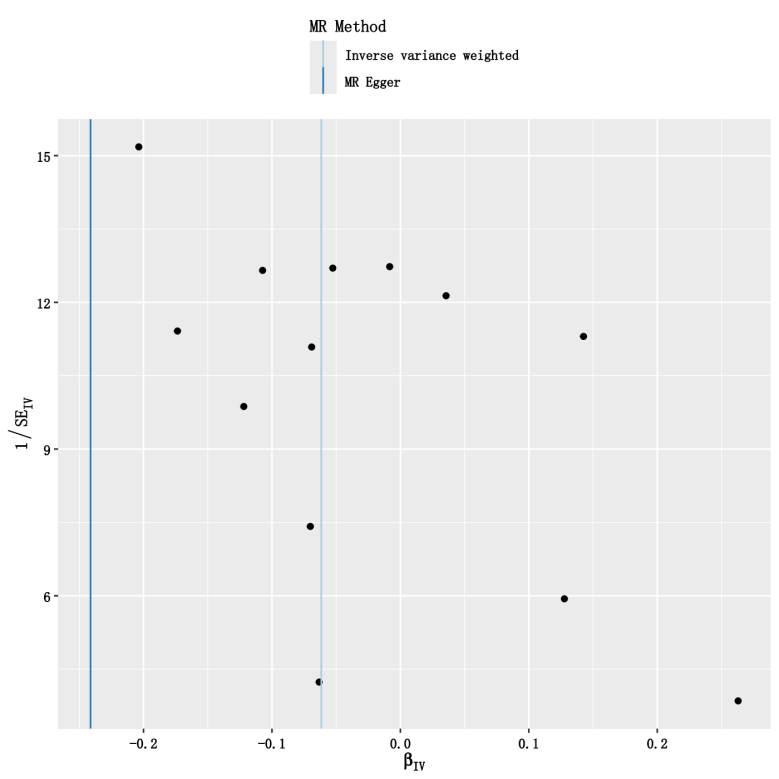
(S)

**Supplementary Figure 5: Funnel plot for gut microbiota and gastric cancer**. (A) C_*Actinobacteria*; (B) C_*Bacteroidia*; (C) F_*Porphyromonadaceae*; (D) G_*Clostridiales*; (E) O_*Bacteroidales*; (F) OTU97_106 (*Ruminococcaceae*); (G) OTU97_108 (*Phascolarctobacterium*); (H) OTU97_130 (*Butyrivibrio*); (I) OTU97_39 (*Proteobacteria*); (J) OTU97_56 (*Ruminococcaceae*); (K) OTU99_121 (*Ruminococcaceae*); (L) OTU99_123 (*Phascolarctobacterium*); (M) OTU99_155 (*Butyrivibrio*); (N) OTU99_40 (*Proteobacteria*); (O) P_*Actinobacteria*; (P) P_*Bacteroidetes*; (Q) TestASV_3 (*Bacteroides*); (R) OTU97_137 (*Catenibacterium*); (S) OTU97_27 (*Bacteroides*).

(A) (B)


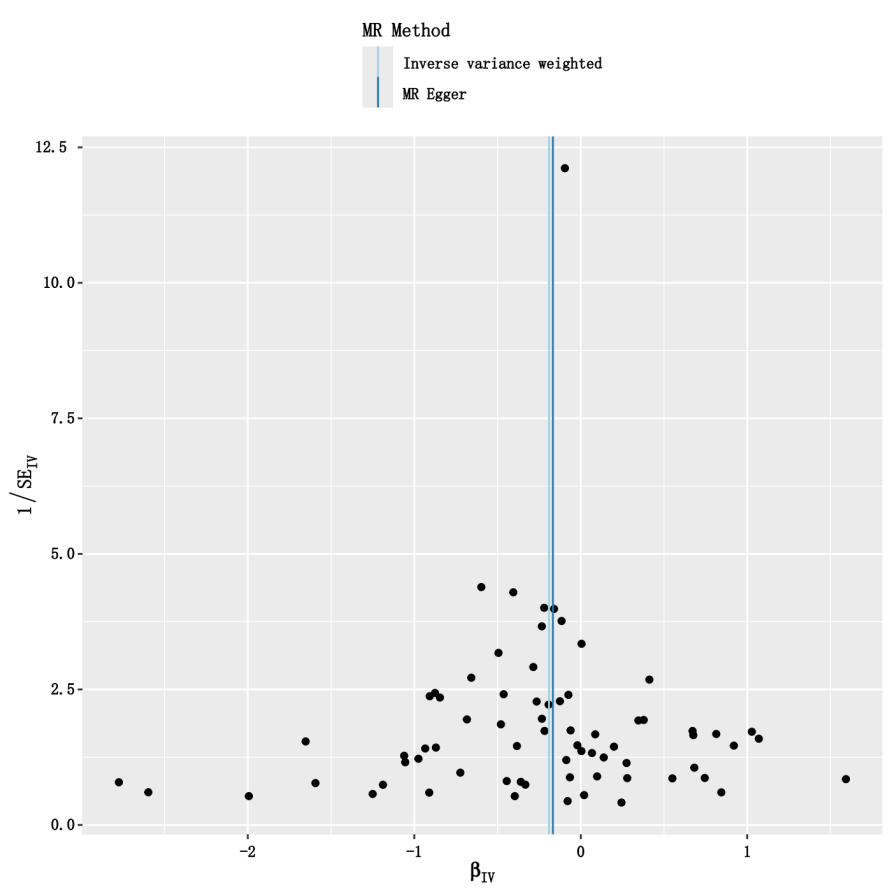

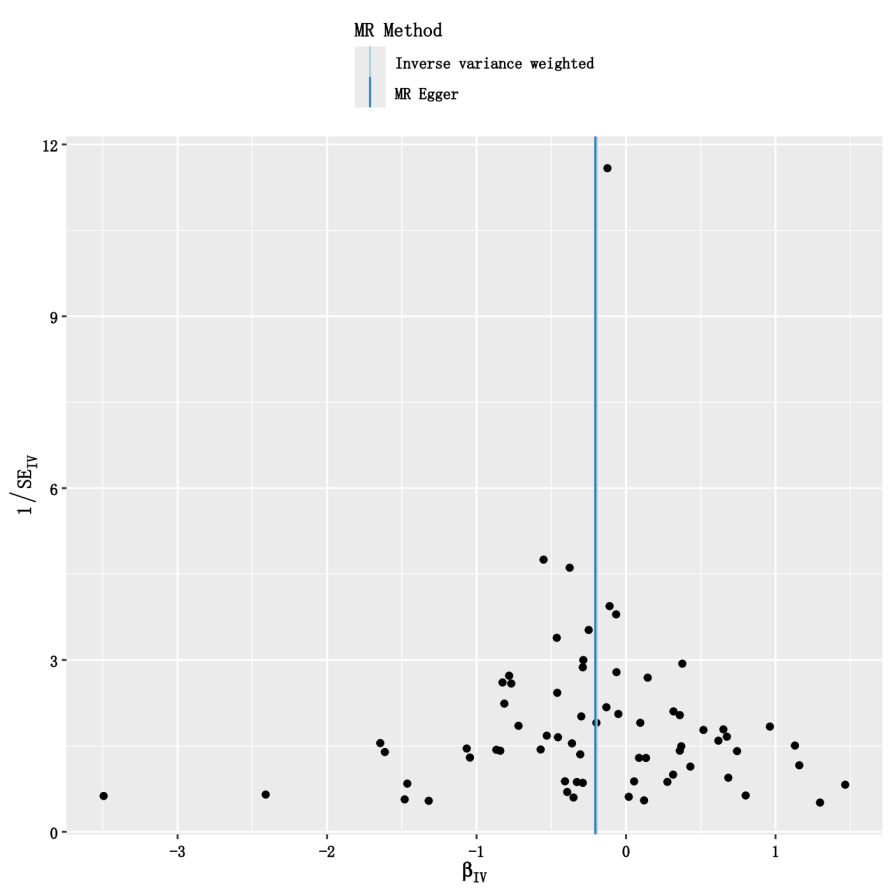

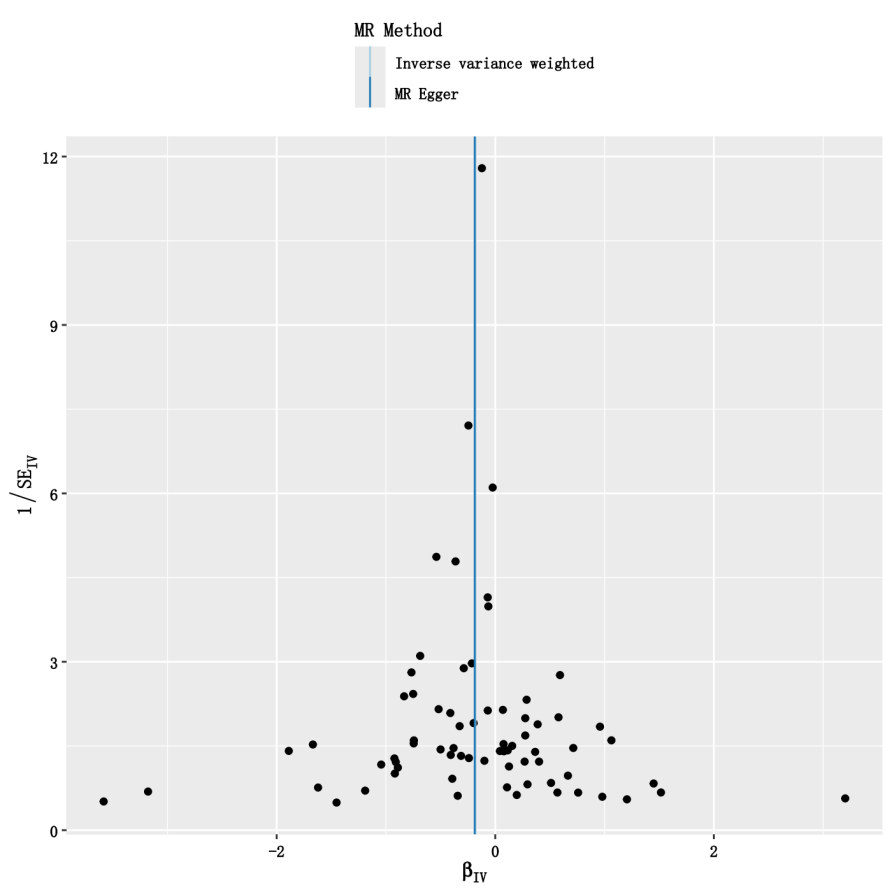

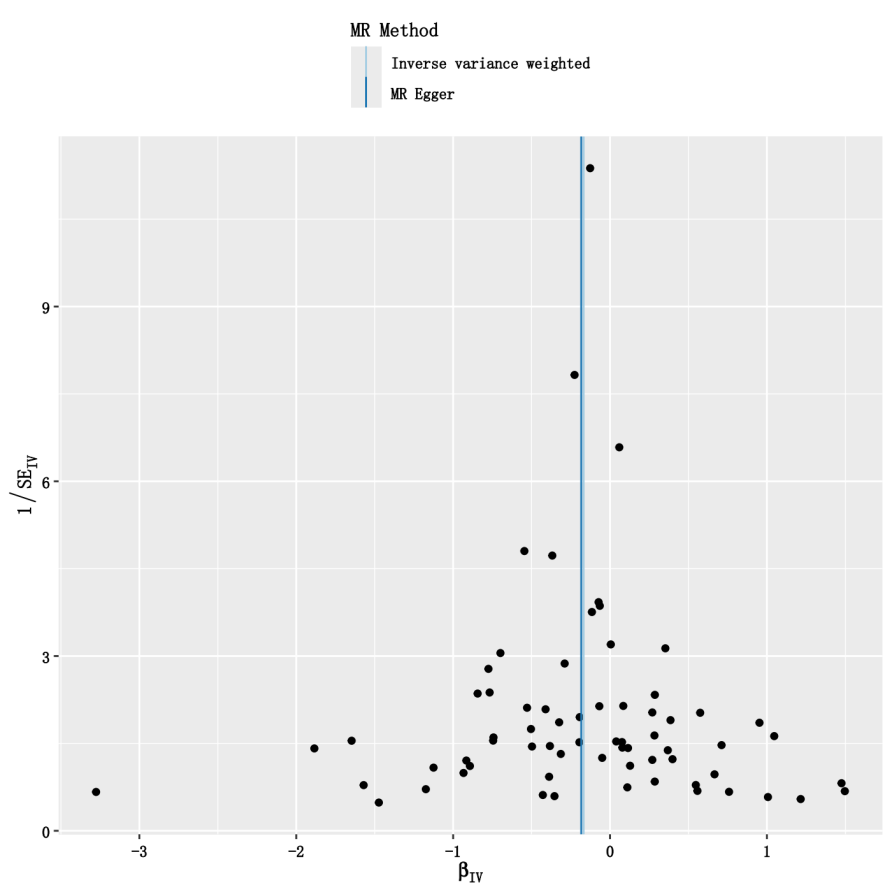


(C) (D)

(E) (F)


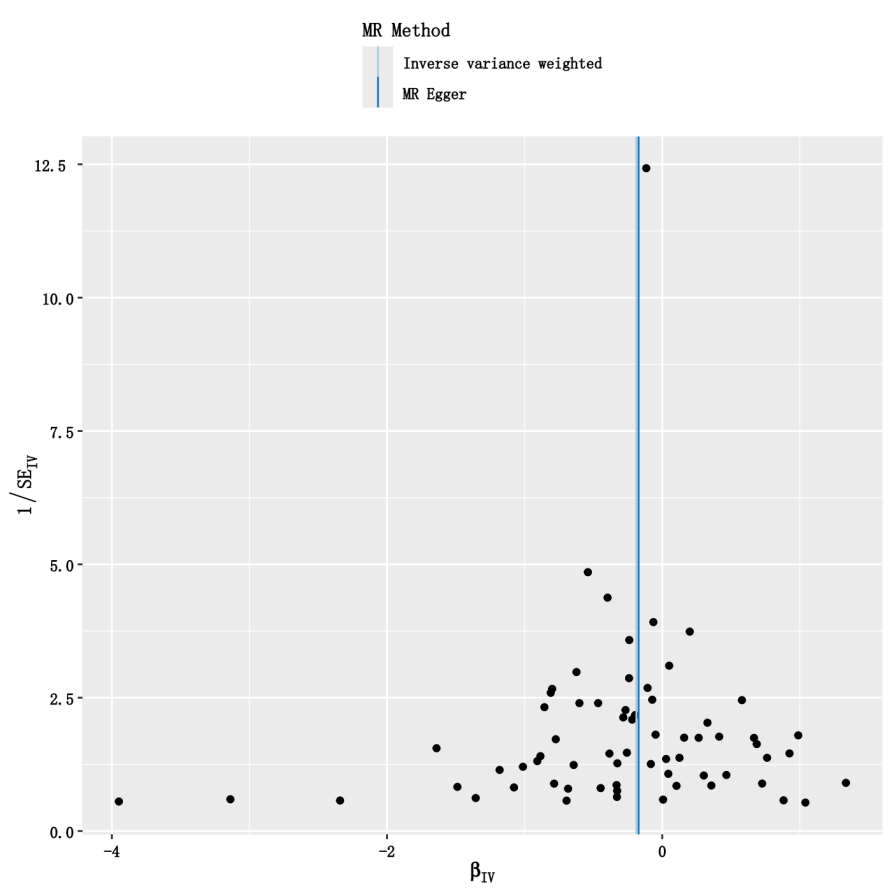

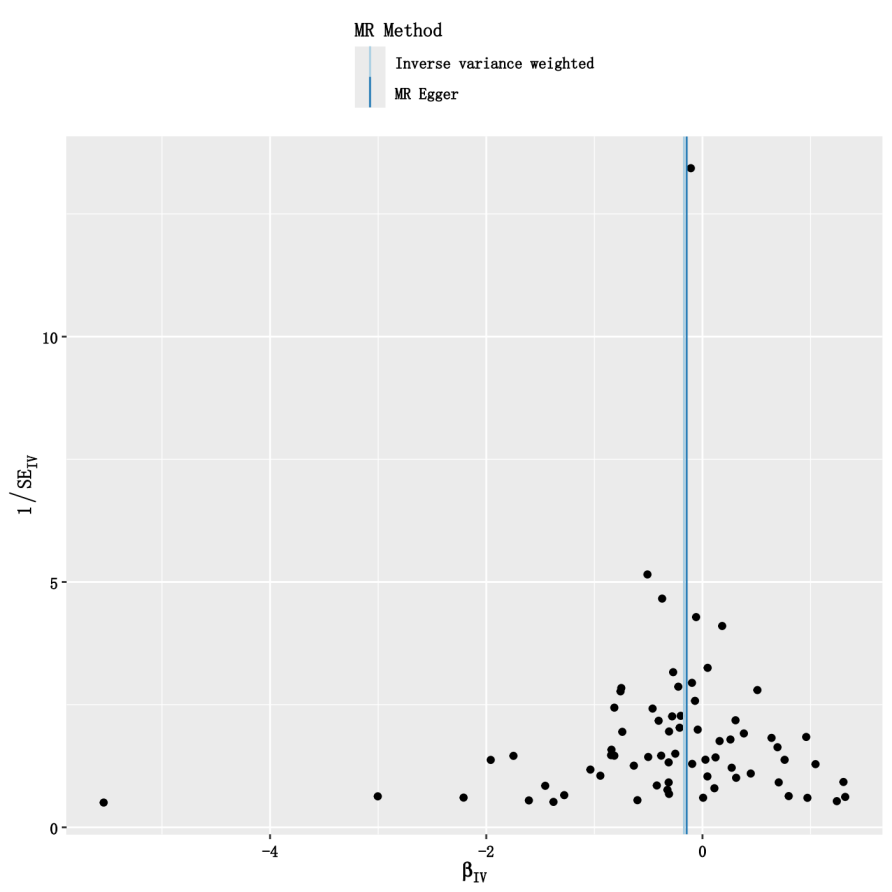

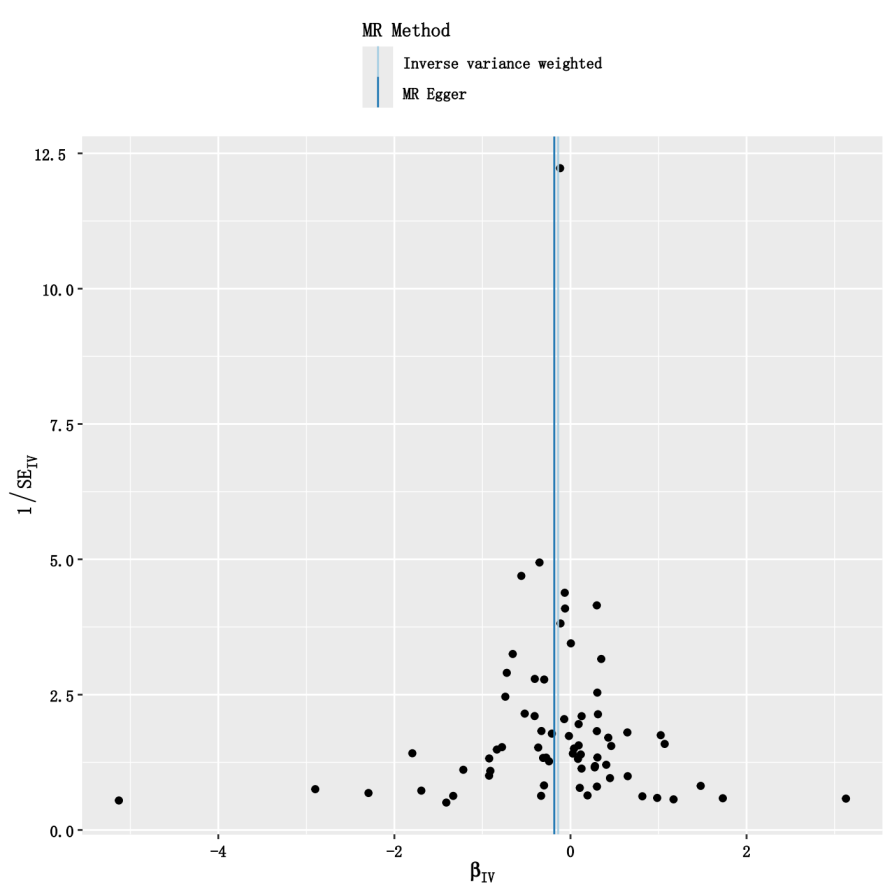


(G)

**Supplementary Figure 6: Funnel plot for blood metabolites and gastric cancer.** (A) Phospholipids in small LDL; (B) Phospholipids in medium LDL; (C) Total lipids in IDL; (D) Concentration of IDL particles; (E) Free cholesterol in small LDL; (F) Free cholesterol in medium LDL; (G) Cholesterol esters in IDL.

(A) (B)


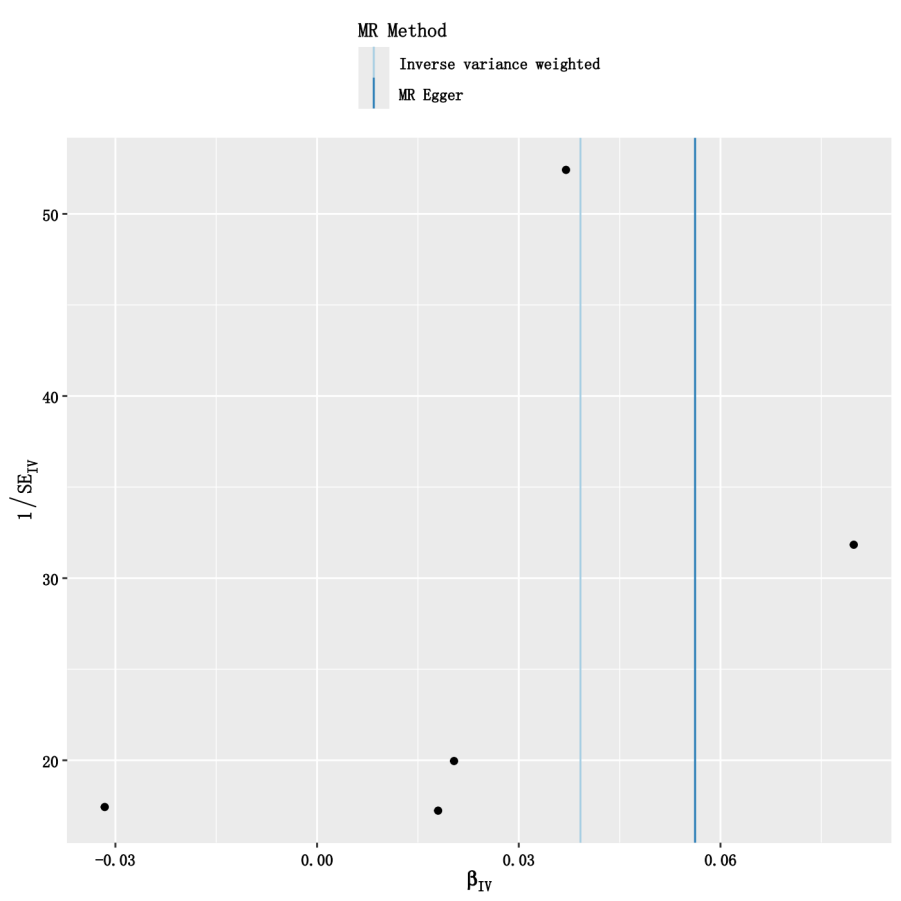

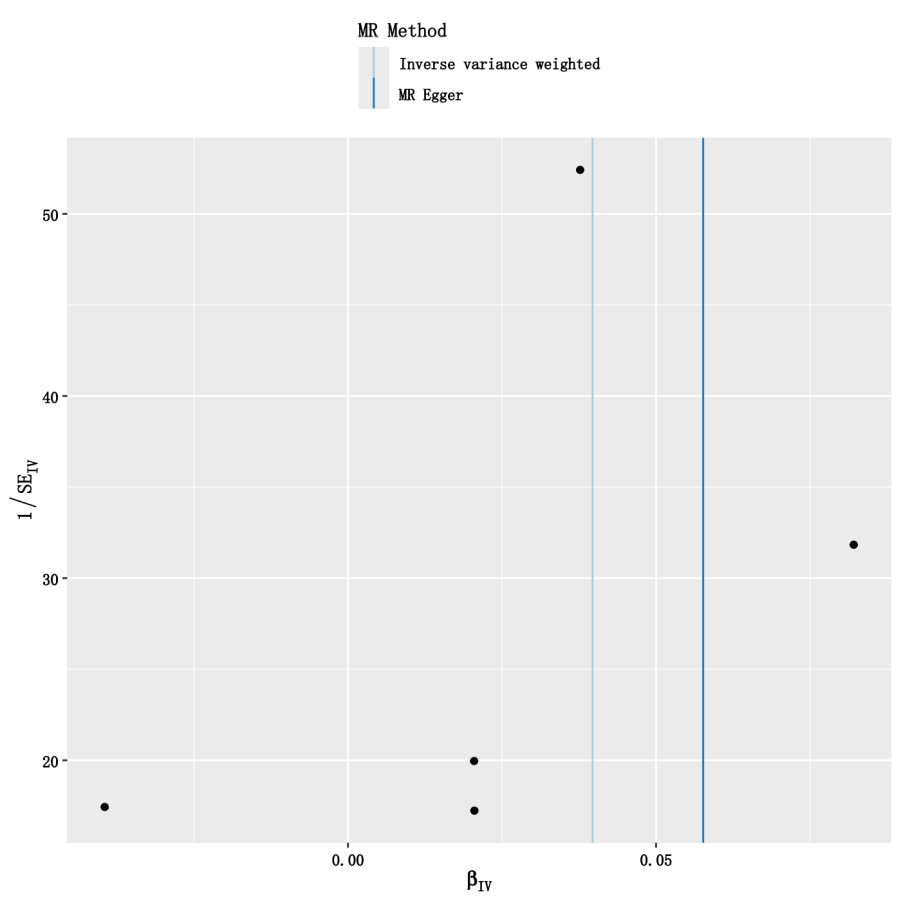

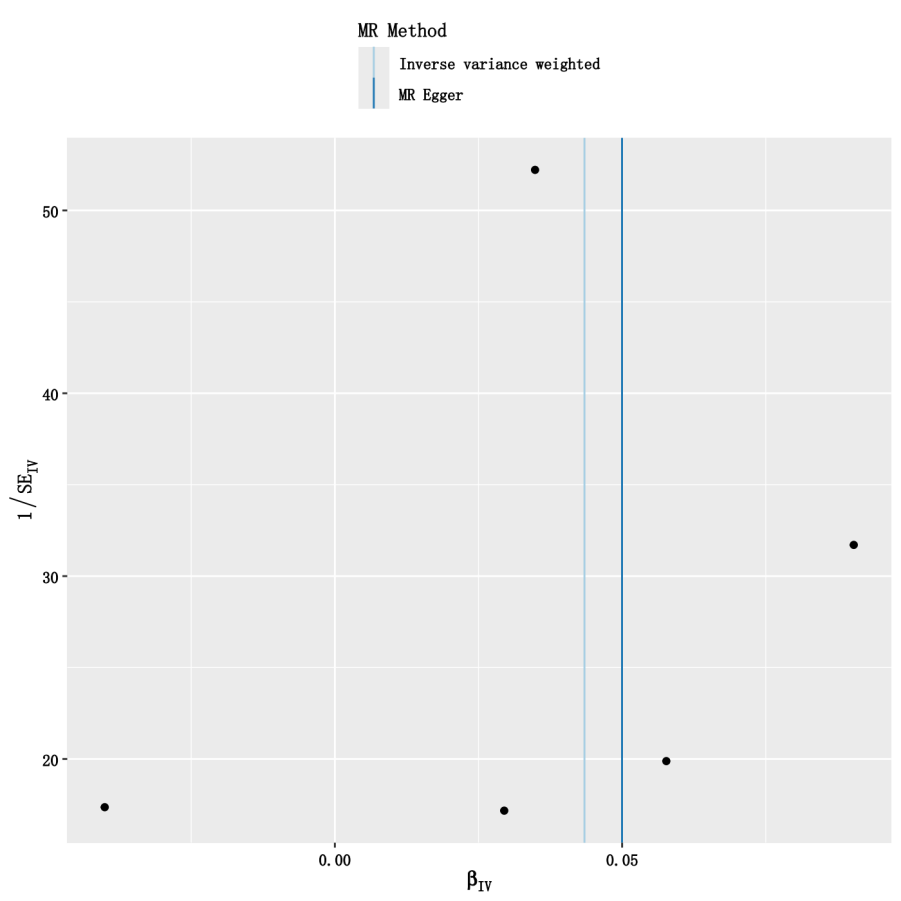

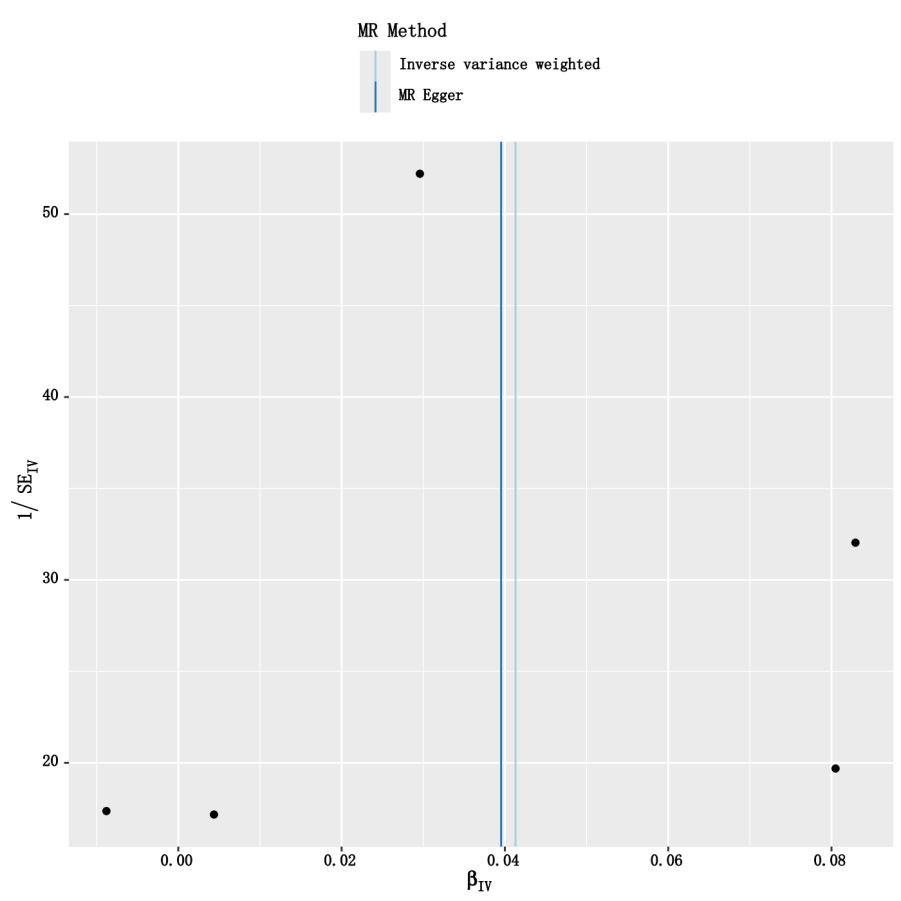


(C) (D)

(E) (F)


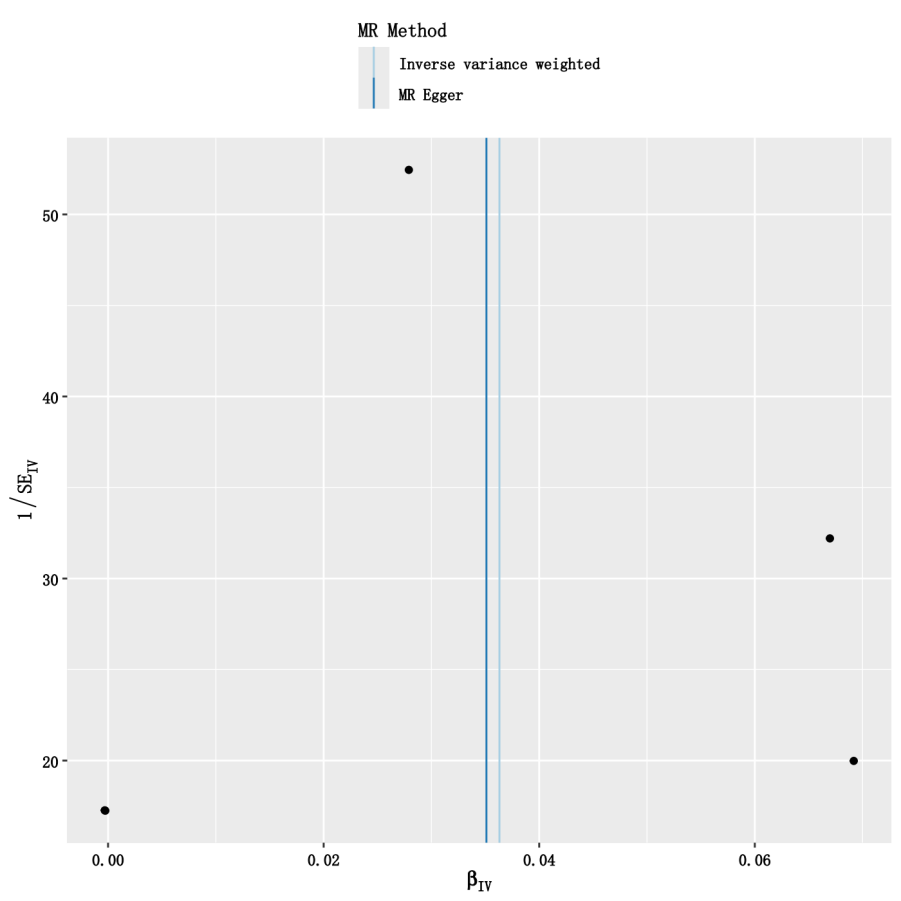

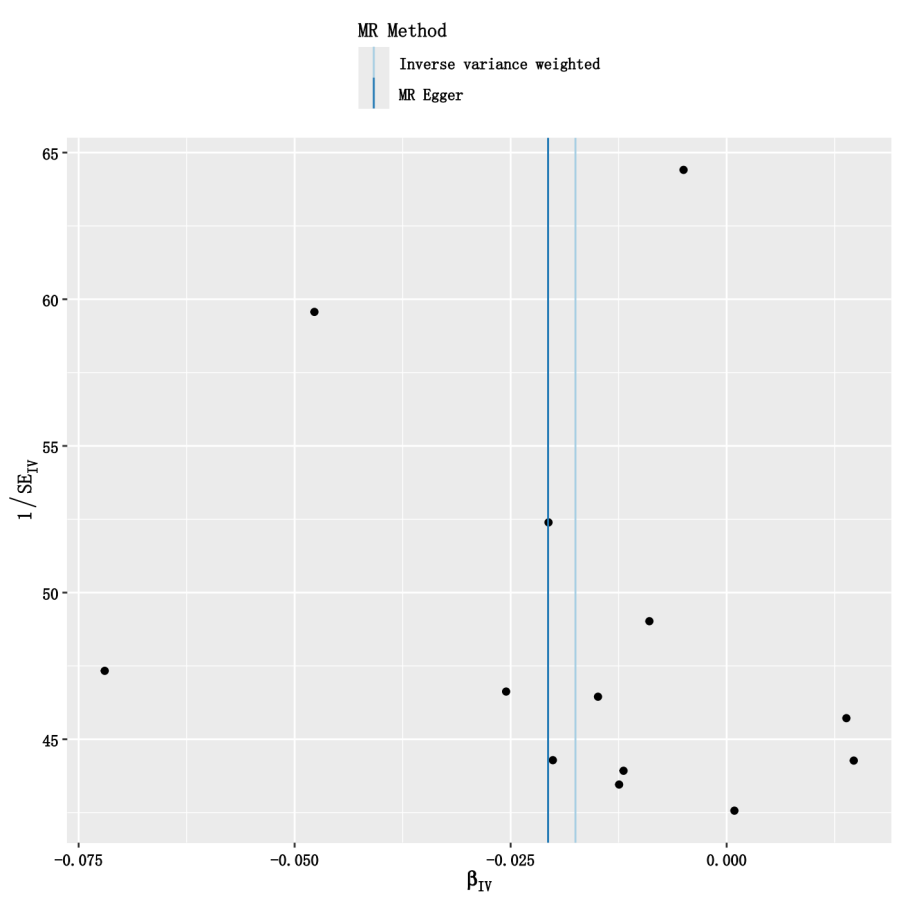


**Supplementary Figure 7: Funnel plot for gut microbiota and blood metabolites.** MR for C_*Bacteroidia* on (A) Total lipids in IDL, (B) Concentration of IDL particles, (C) Phospholipids in medium LDL, (D) Phospholipids in small LDL, and (E) Free cholesterol in small LDL; MR for OTU97_27 (*Bacteroides*) on (F) Free cholesterol in small LDL.

(A) (B)


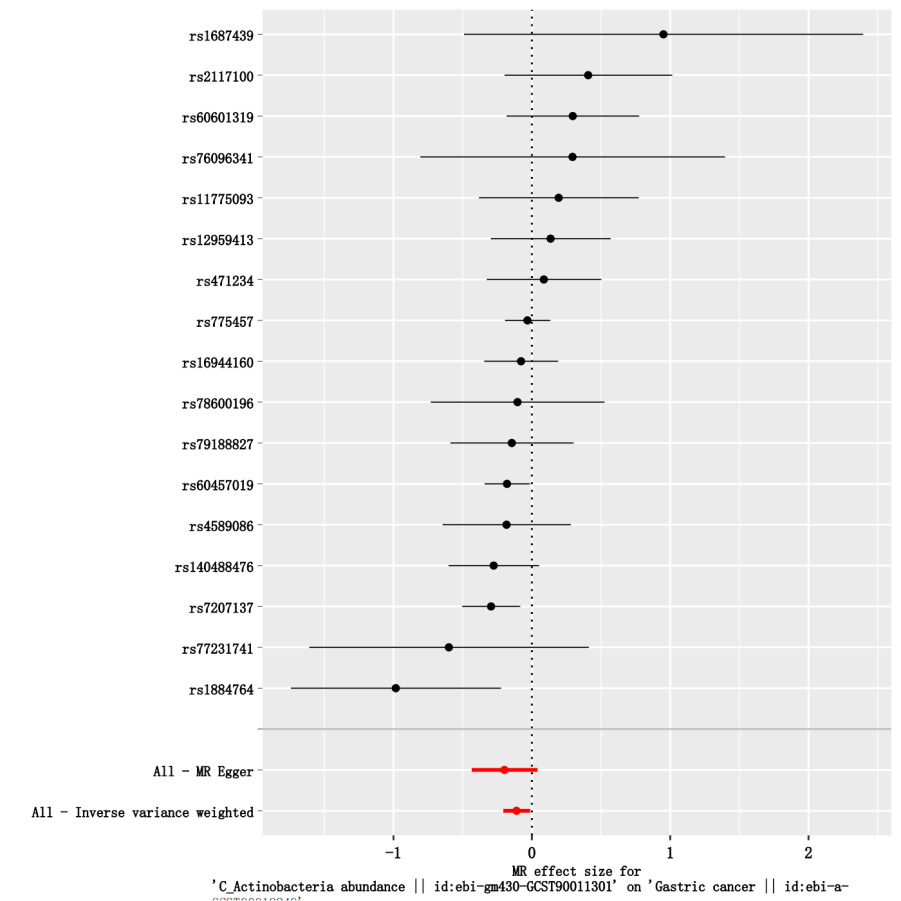

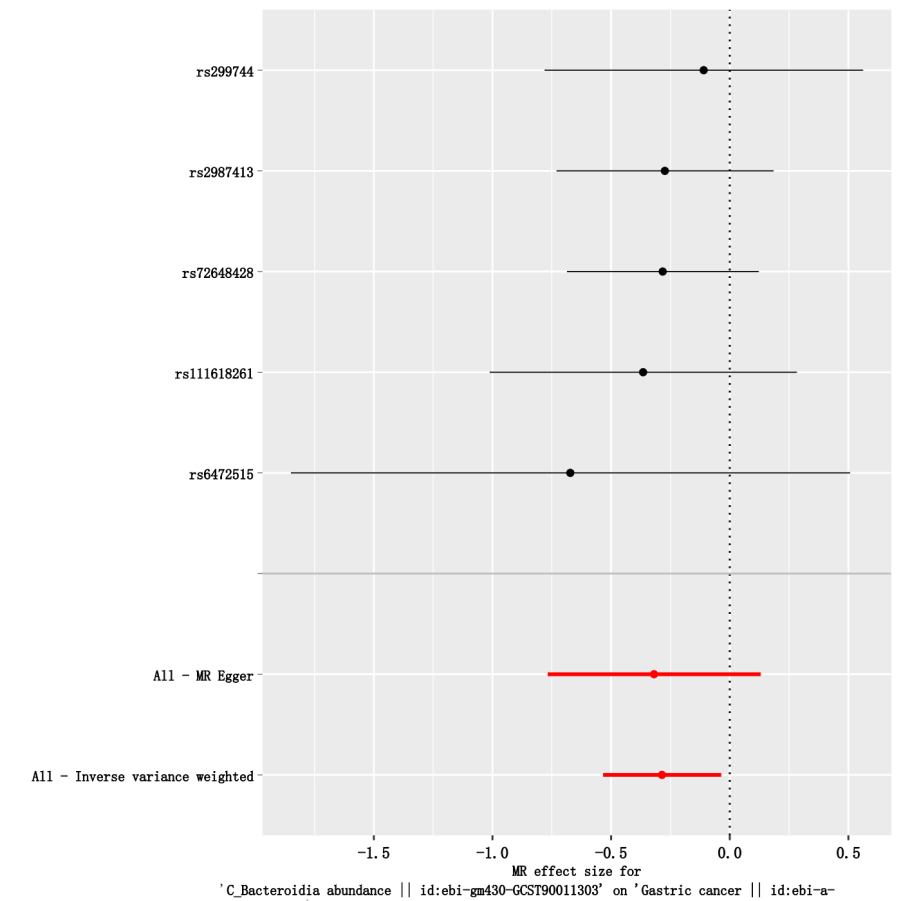

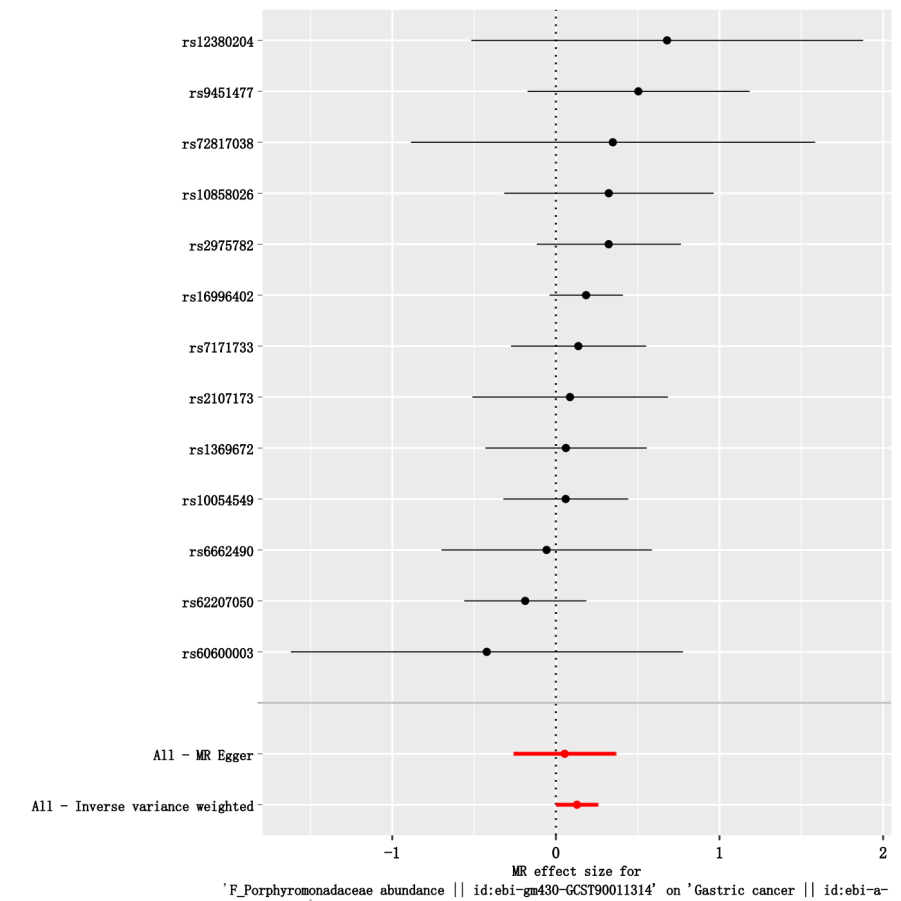

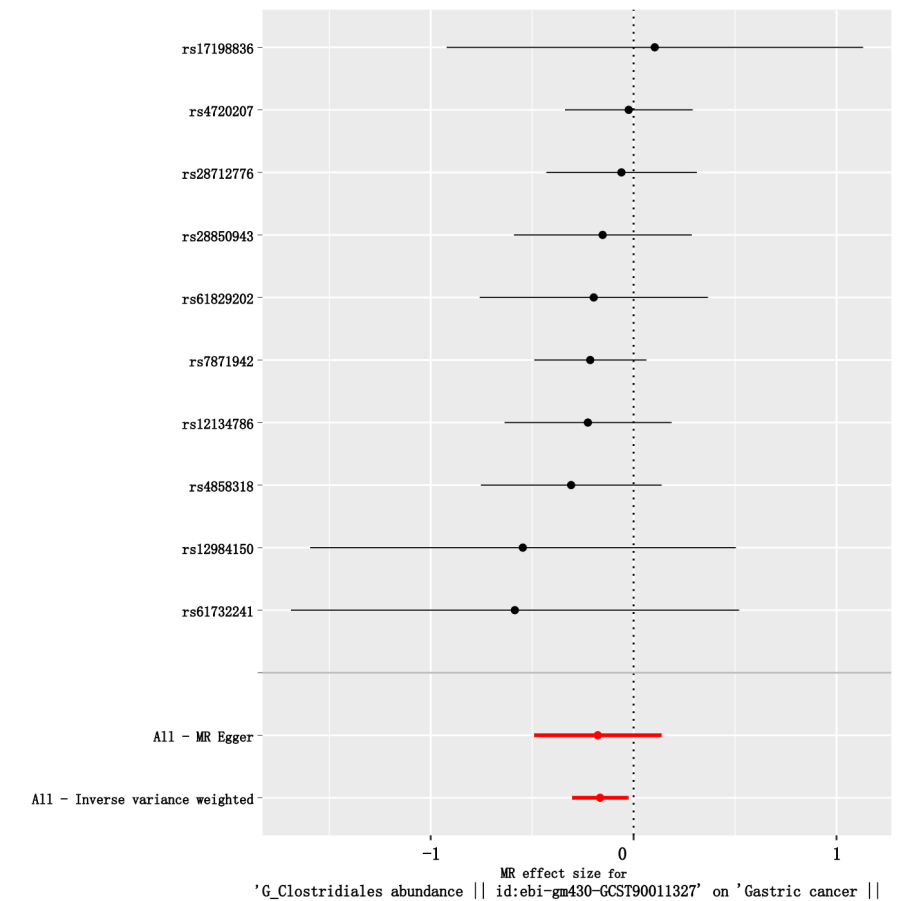


(C) (D)

(E) (F)


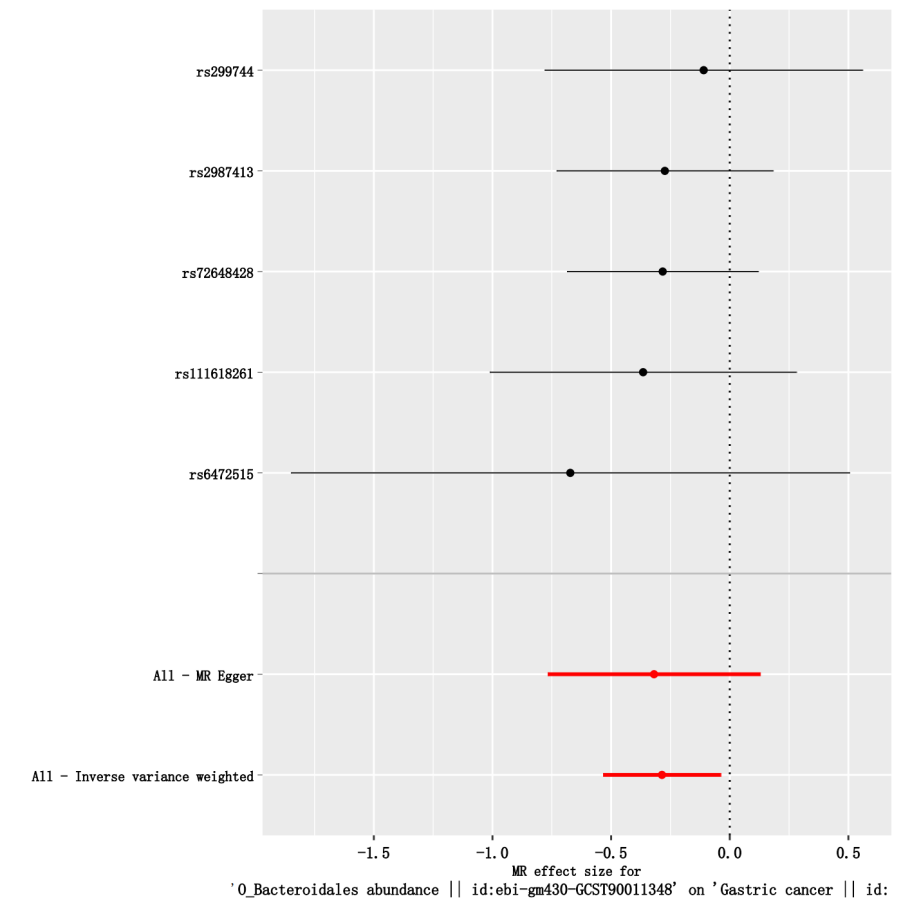

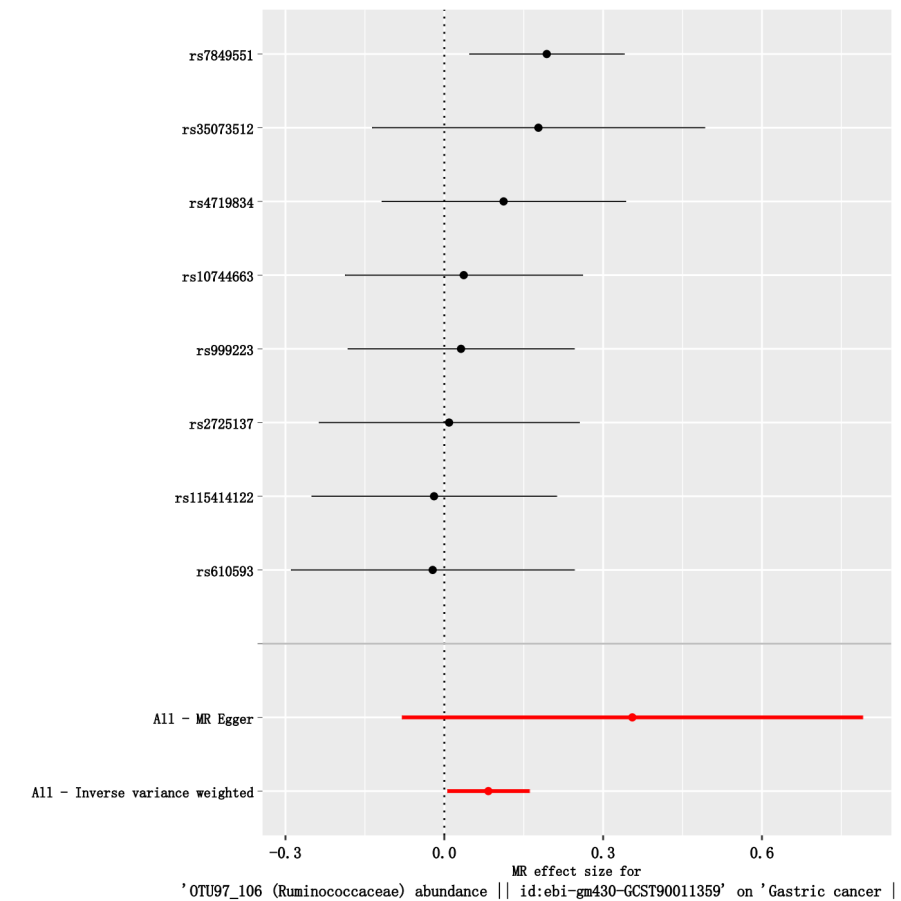

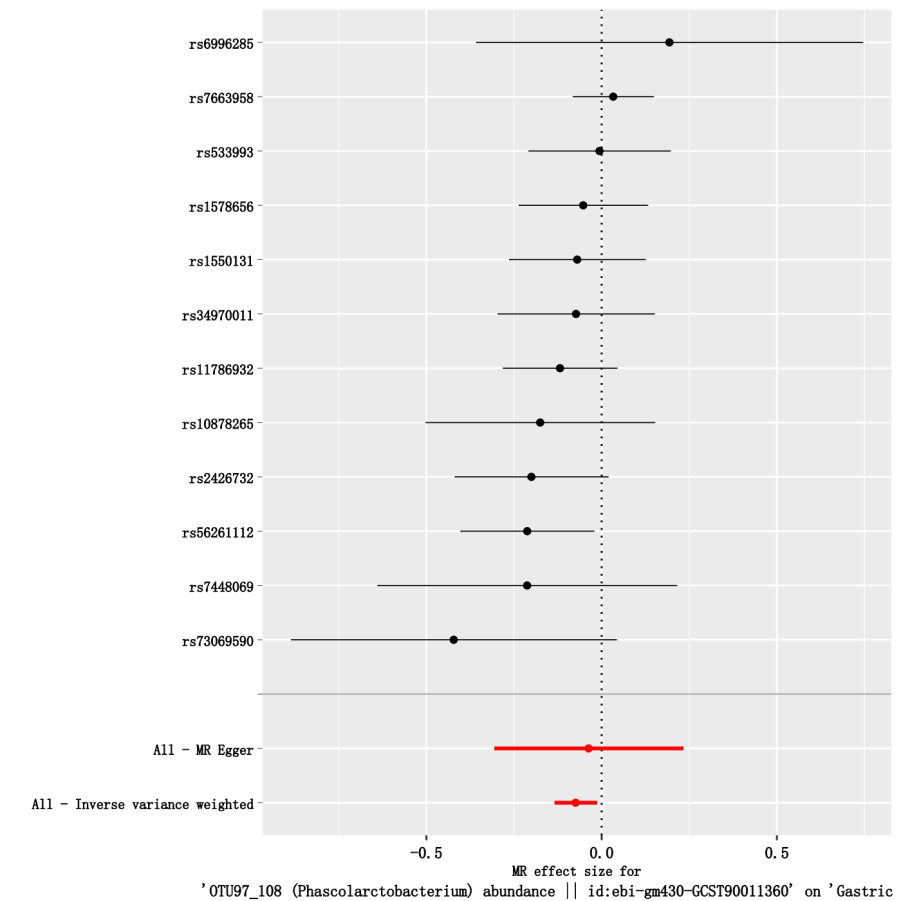

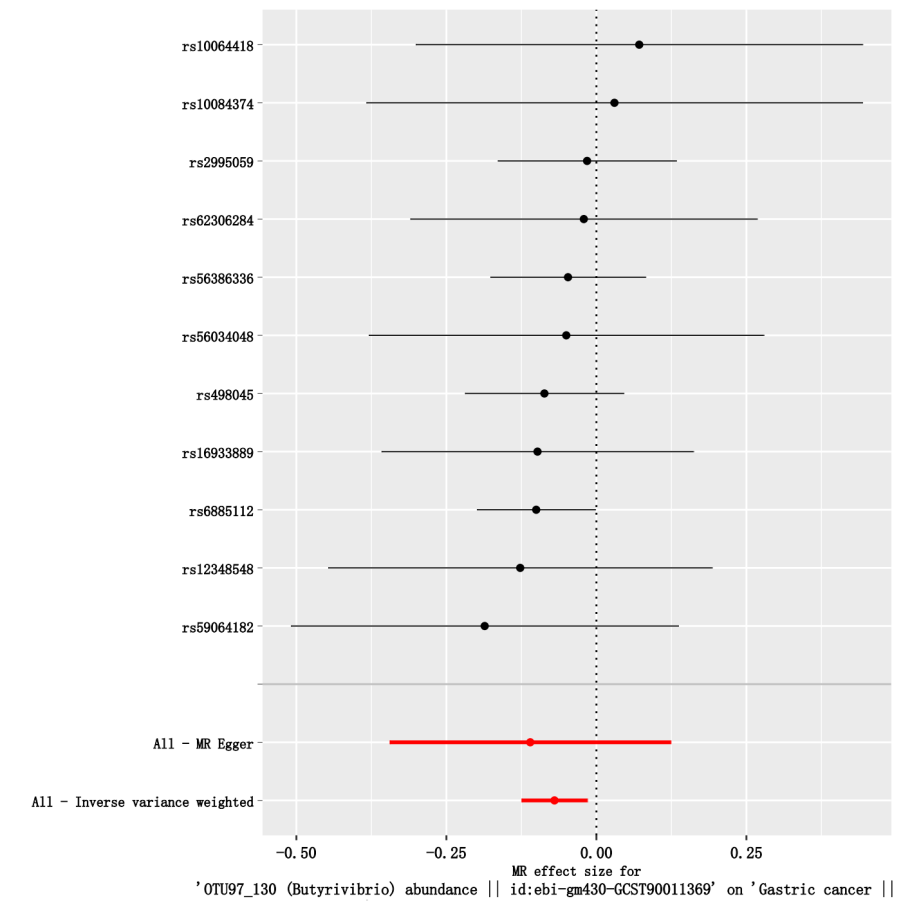


(G) (H)

1. (J)


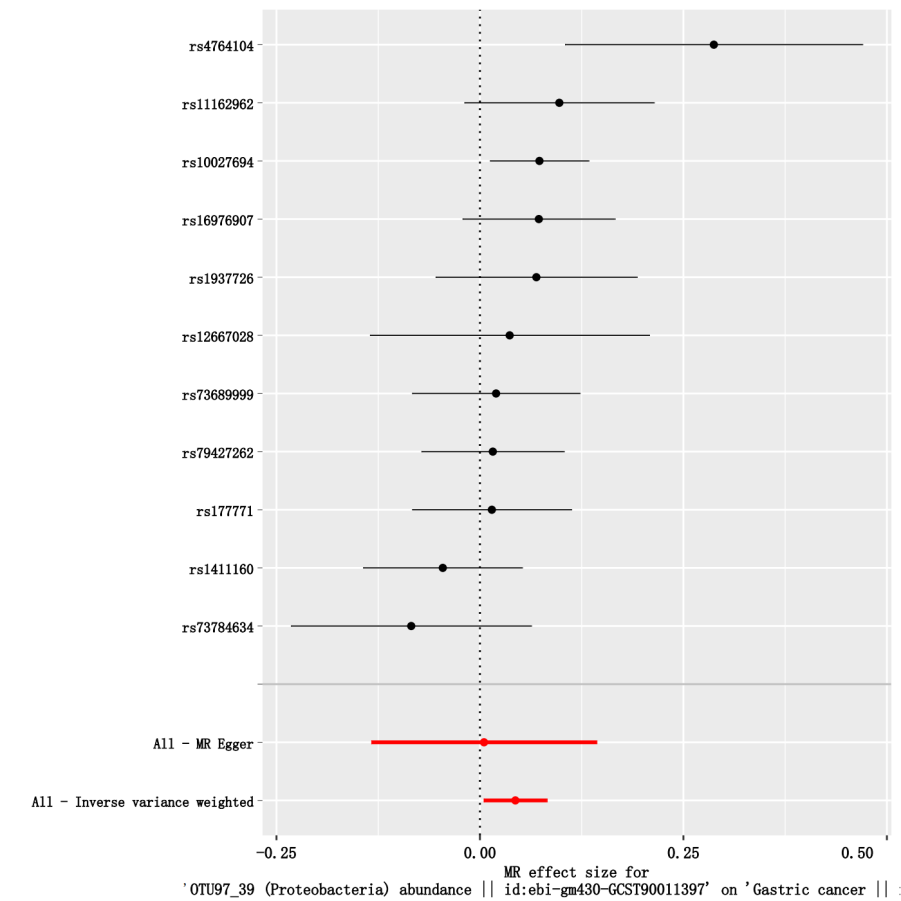

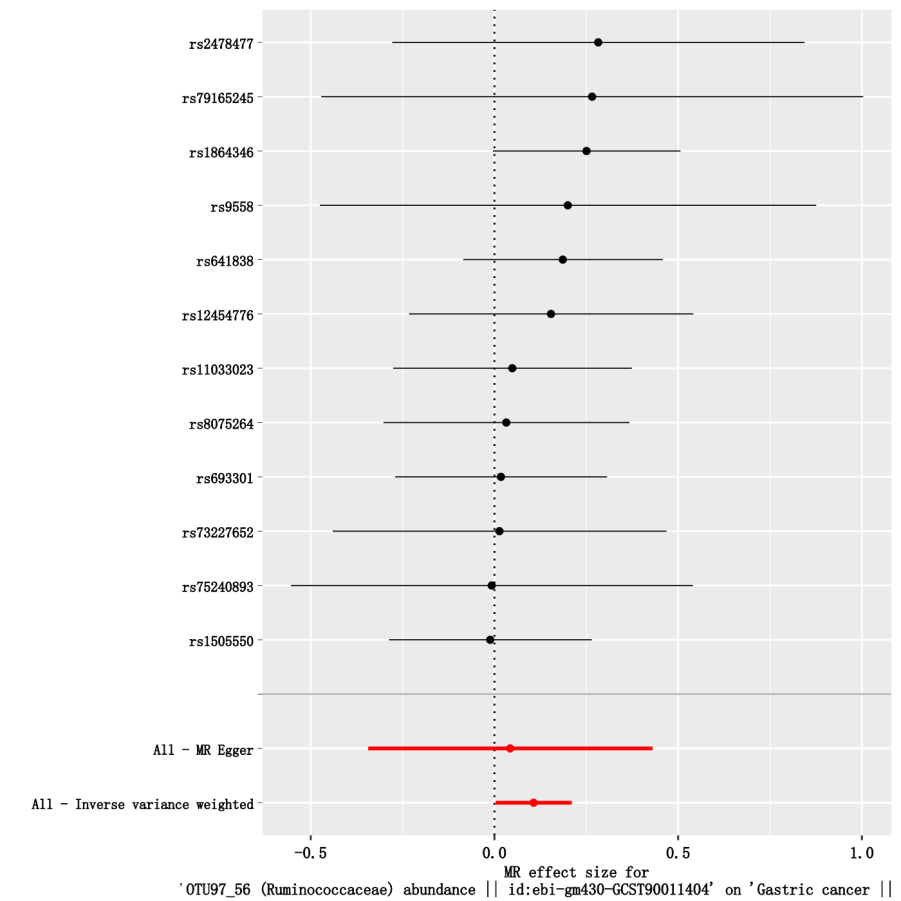

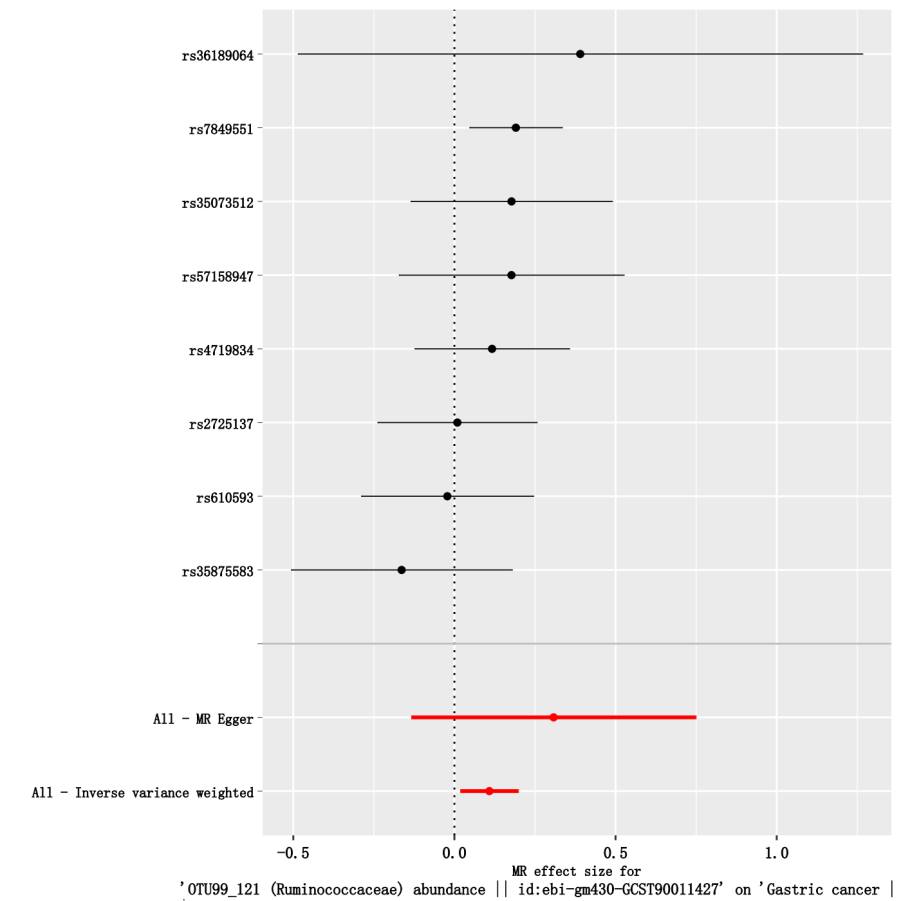

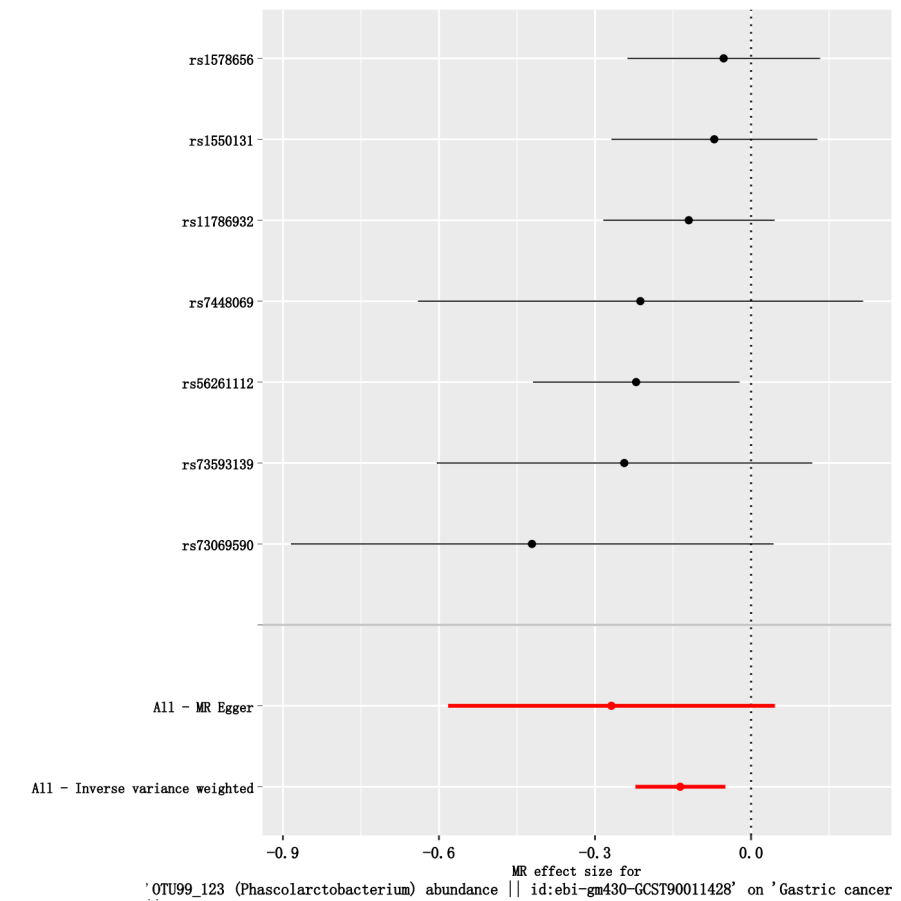


(K) (L)

(M) (N)


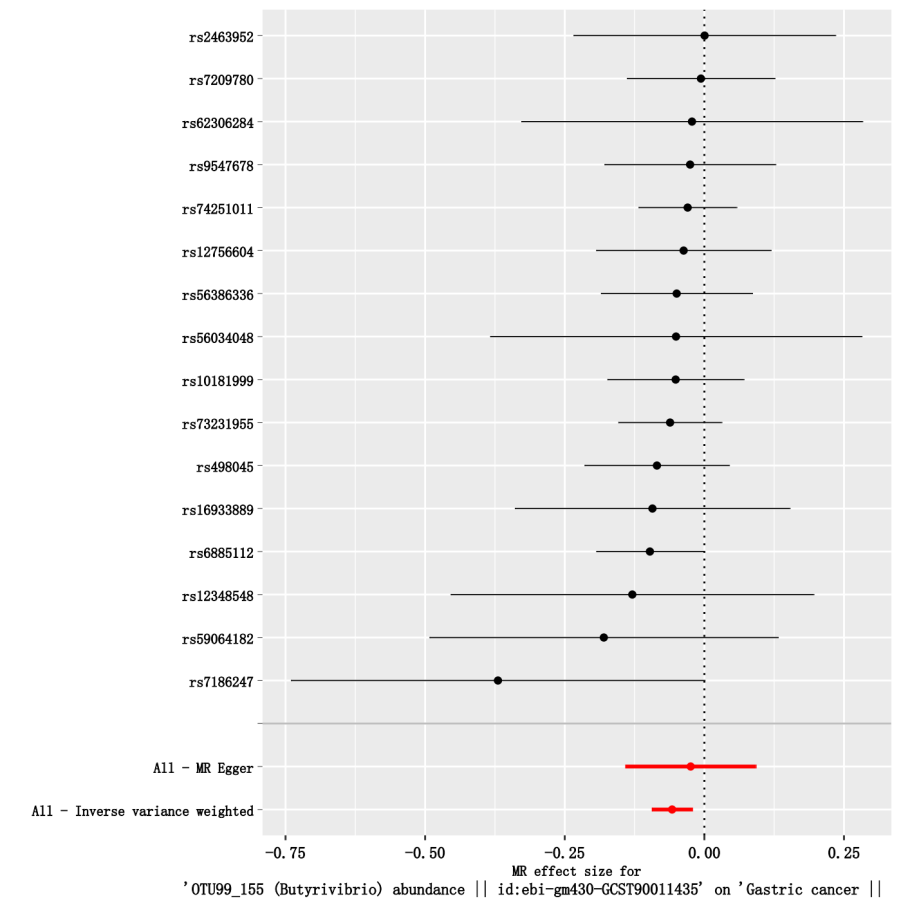

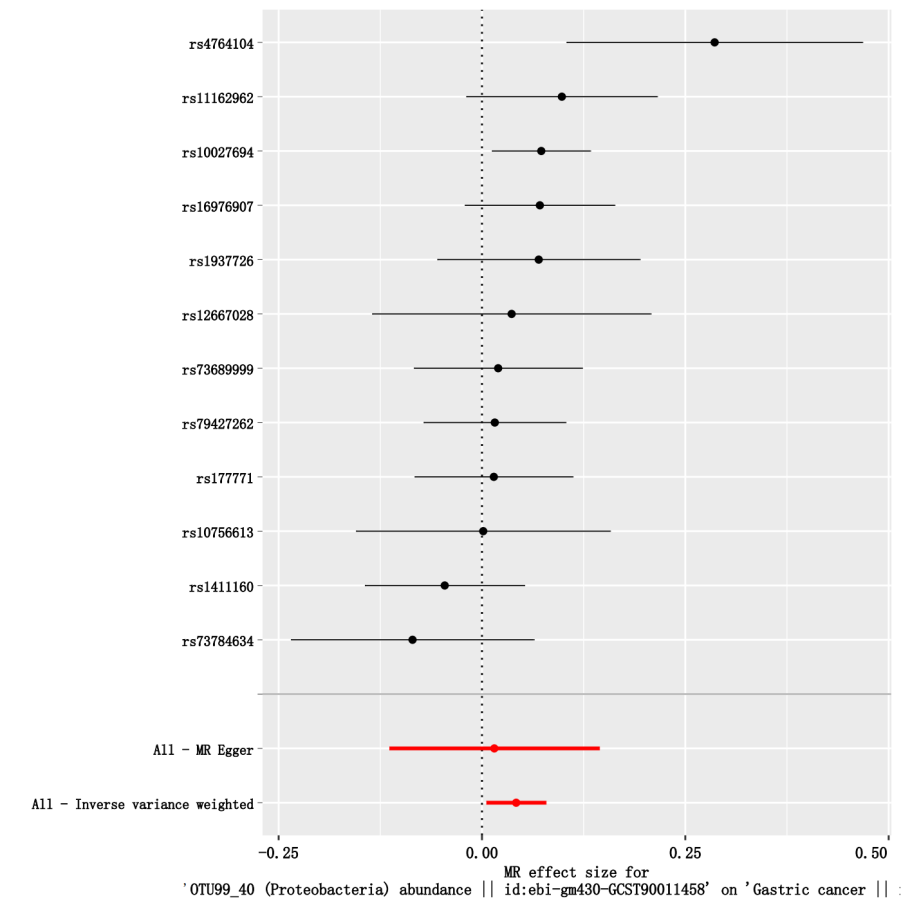

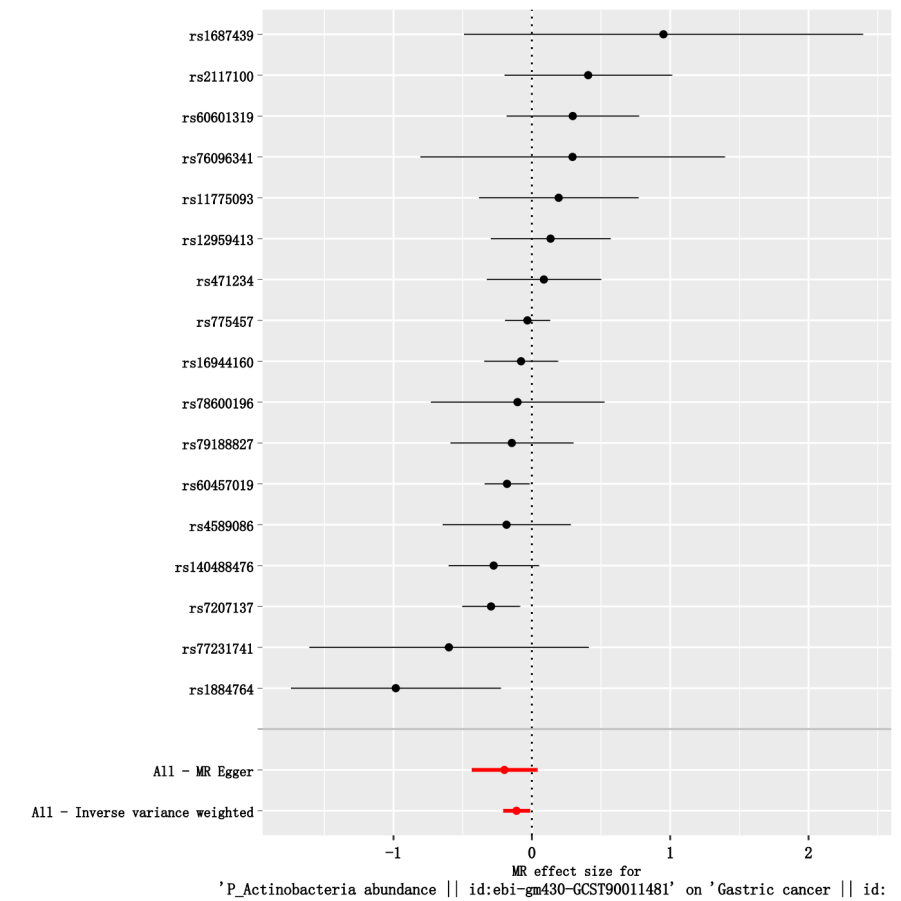

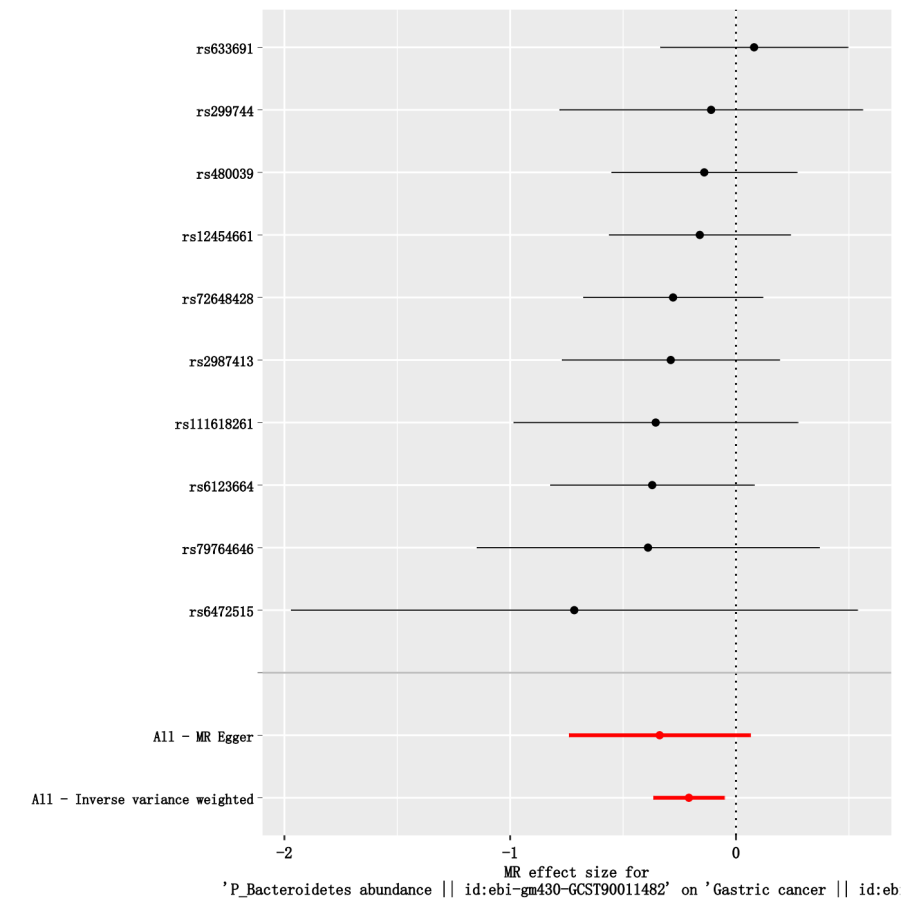


(O) (P)

1. (R)


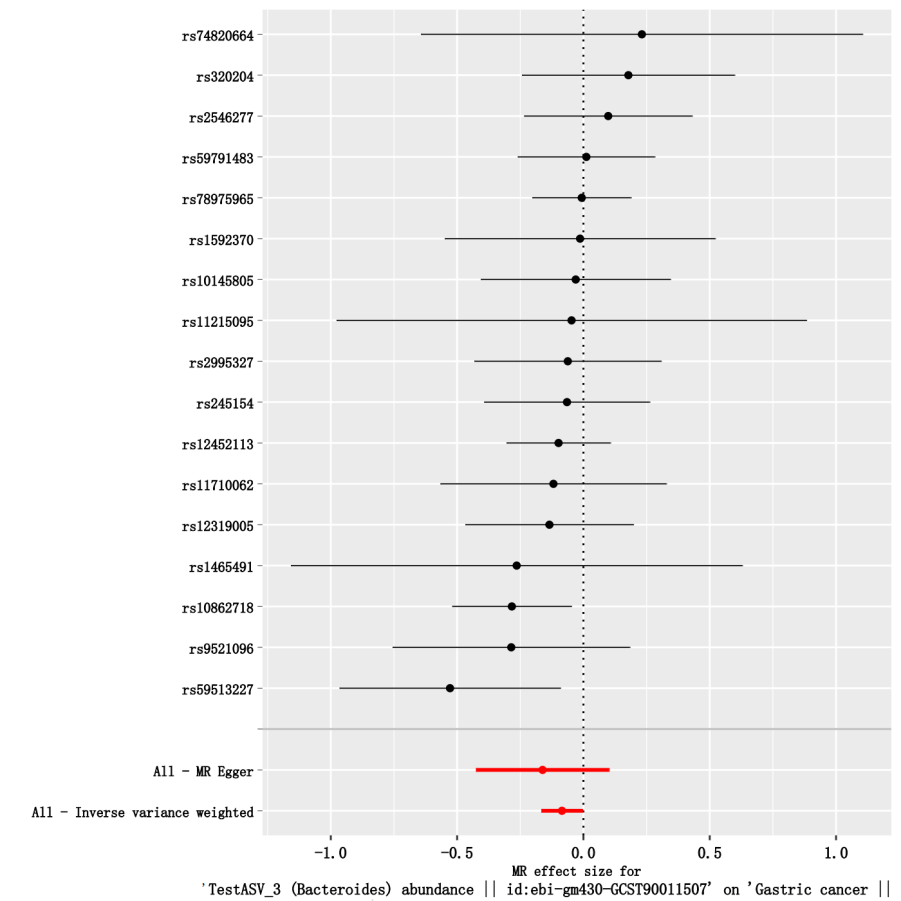

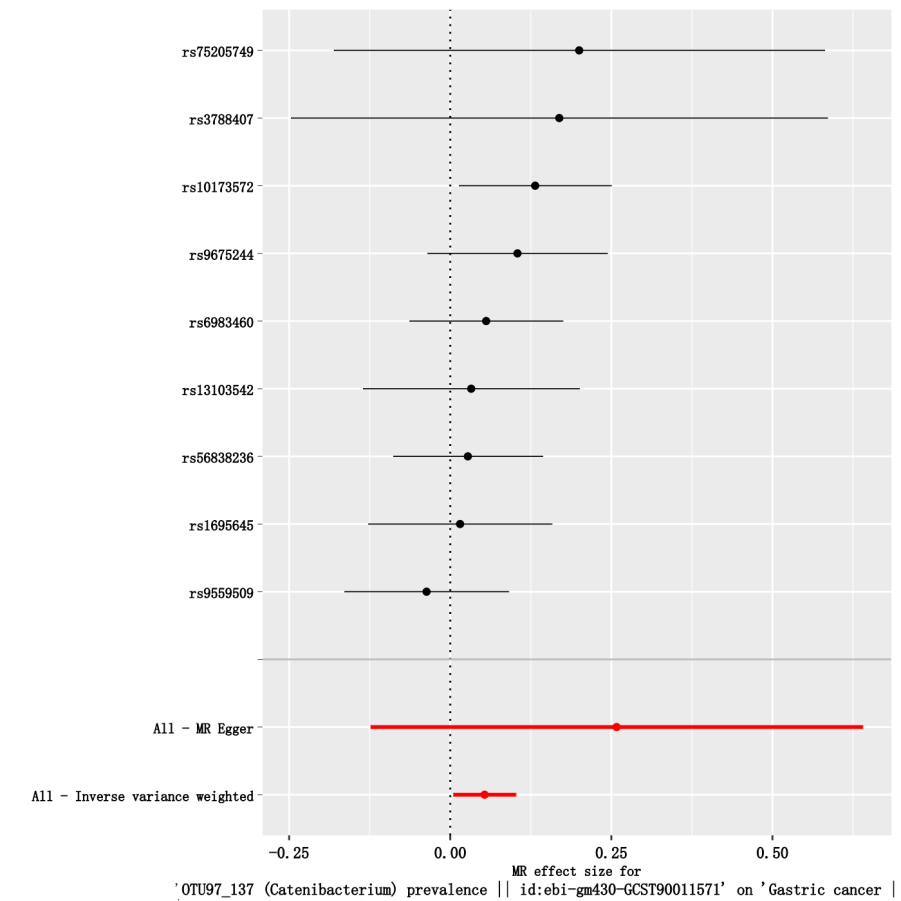

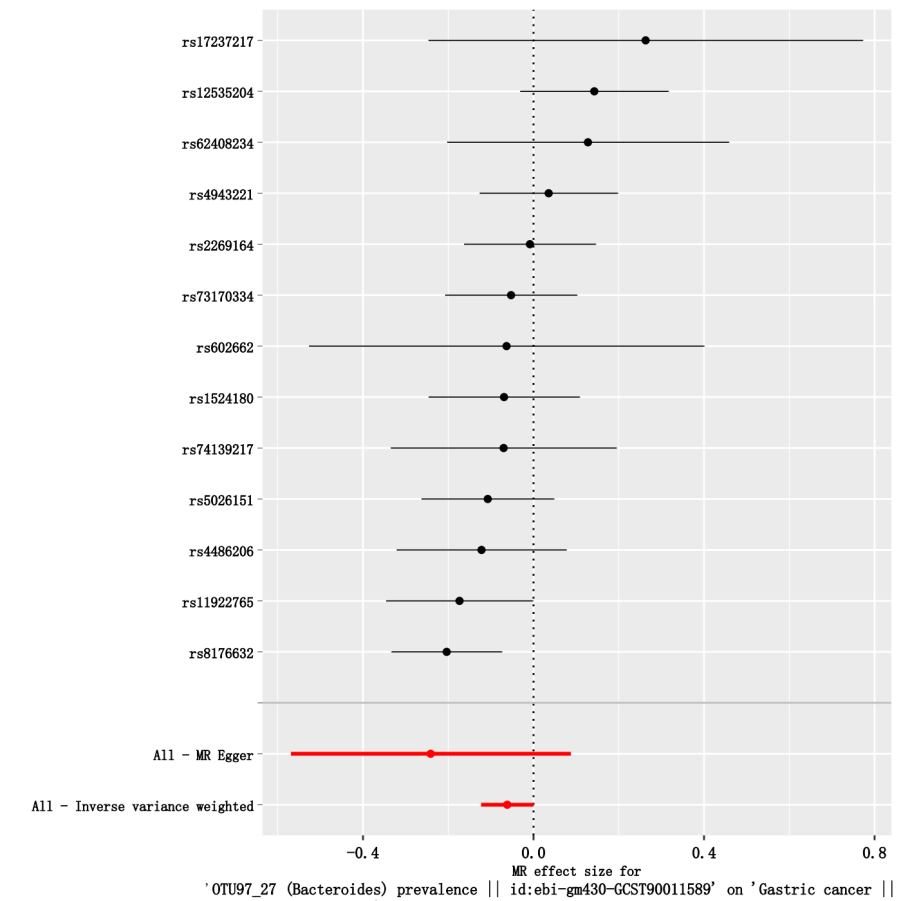


(S)

**Supplementary Figure 8: Forest plot of the causal effects of gut microbiota associated SNPs on gastric cancer.** (A) C_*Actinobacteria*; (B) C_*Bacteroidia*; (C) F_*Porphyromonadaceae*; (D) G_*Clostridiales*; (E) O_*Bacteroidales*; (F) OTU97_106 (*Ruminococcaceae*); (G) OTU97_108 (*Phascolarctobacterium*); (H) OTU97_130 (*Butyrivibrio*); (I) OTU97_39 (*Proteobacteria*); (J) OTU97_56 (*Ruminococcaceae*); (K) OTU99_121 (*Ruminococcaceae*); (L) OTU99_123 (*Phascolarctobacterium*); (M) OTU99_155 (*Butyrivibrio*); (N) OTU99_40 (*Proteobacteria*); (O) P_*Actinobacteria*; (P) P_*Bacteroidetes*; (Q) TestASV_3 (*Bacteroides*); (R) OTU97_137 (*Catenibacterium*); (S) OTU97_27 (*Bacteroides*).

(A) (B)


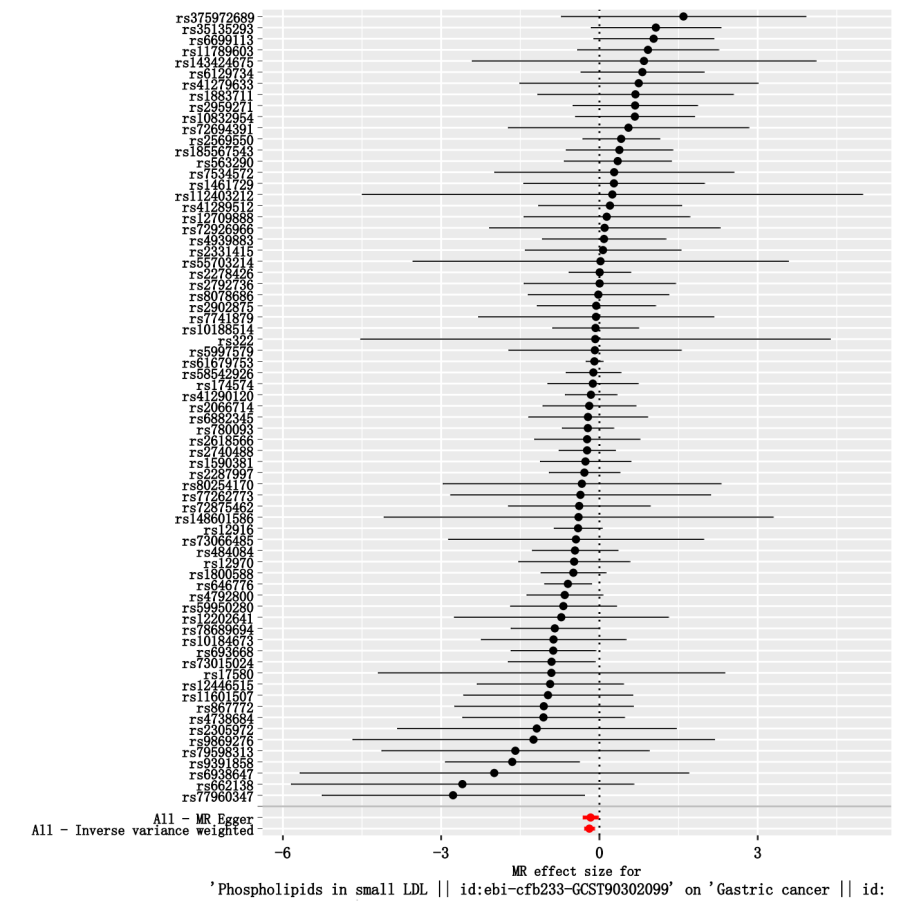

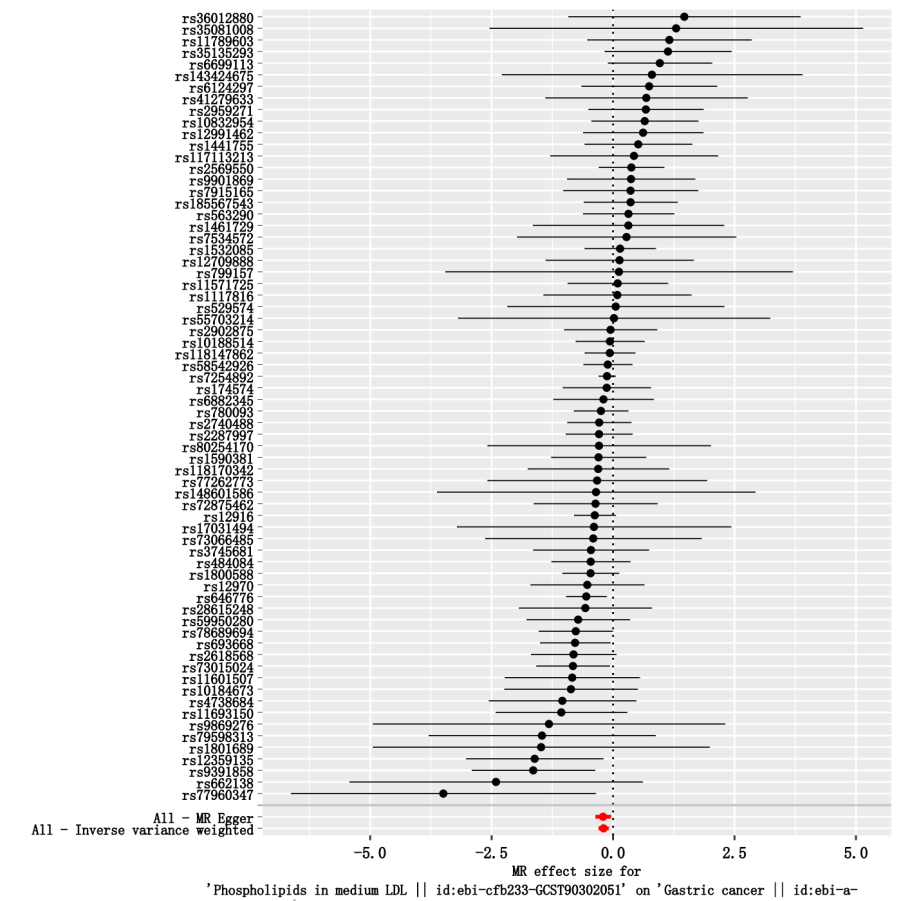

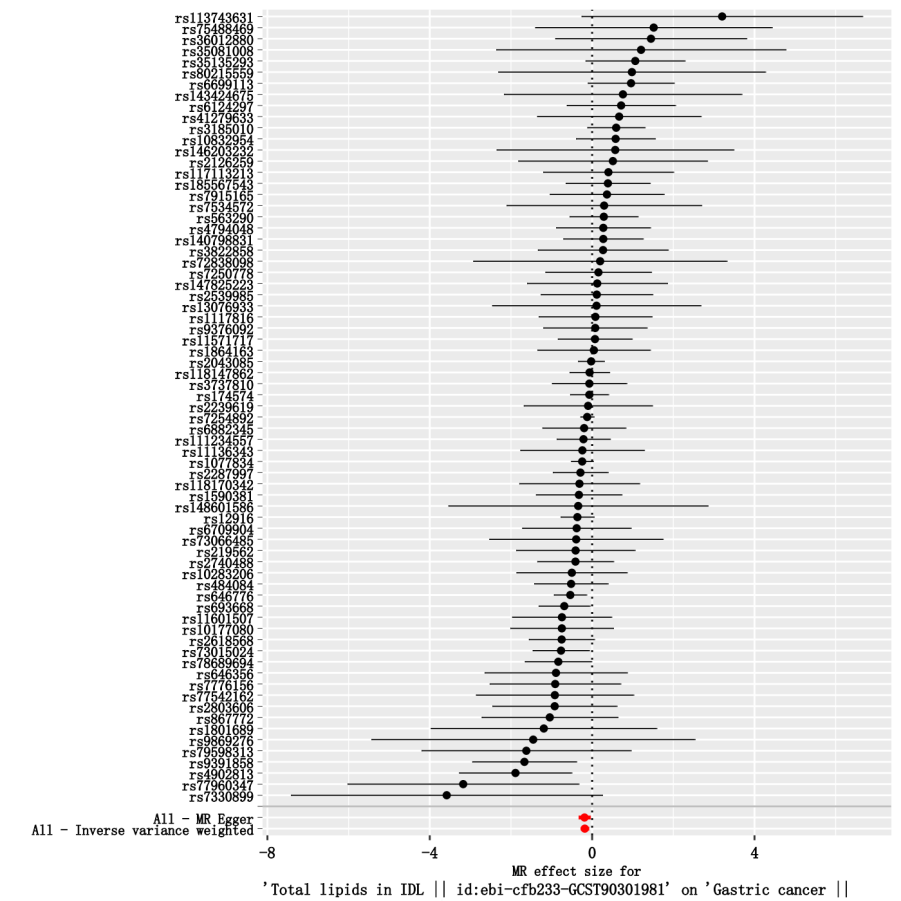

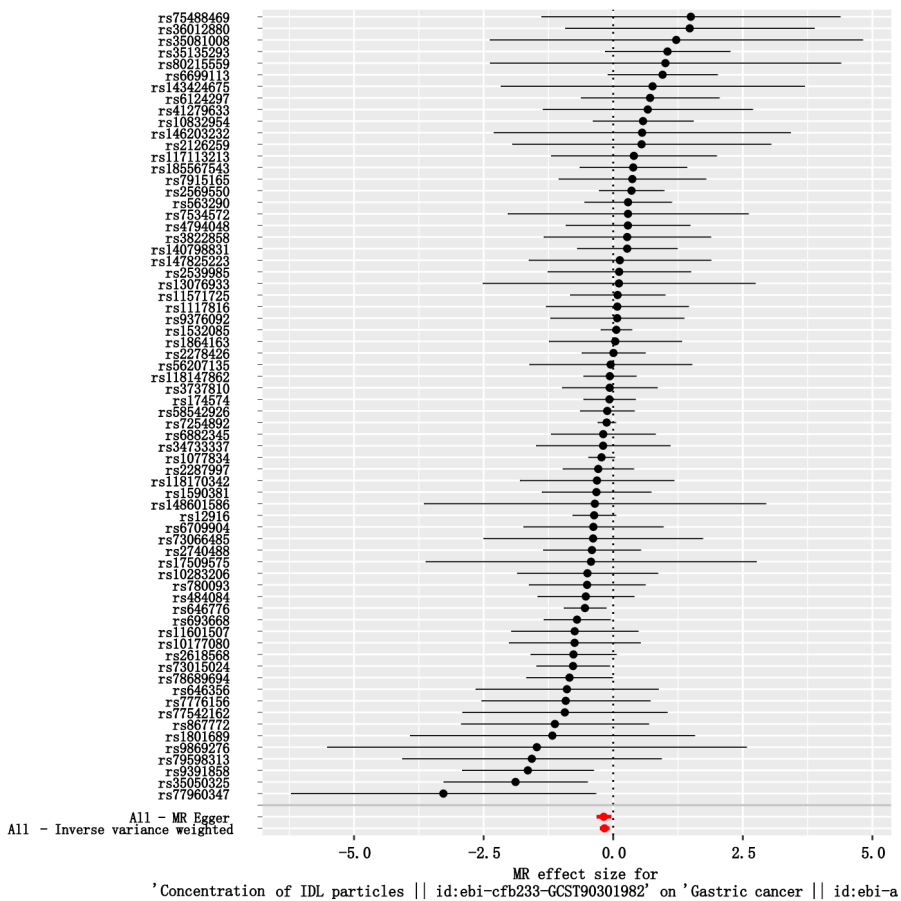


(C) (D)

(E) (F)


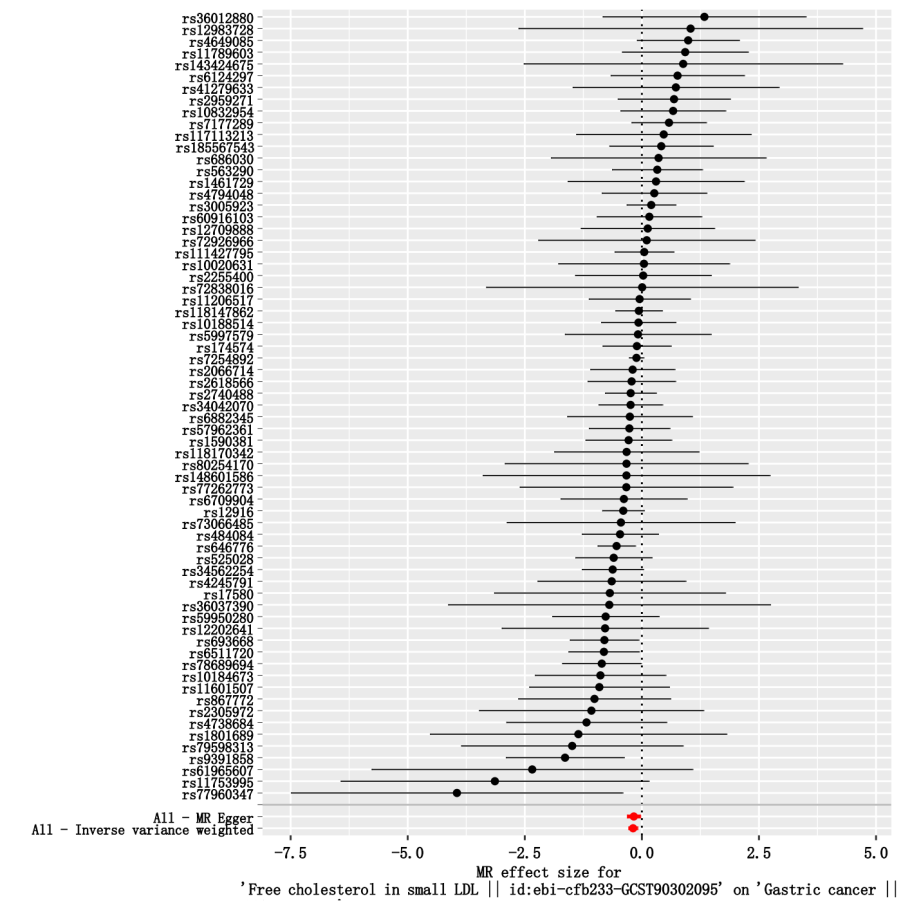

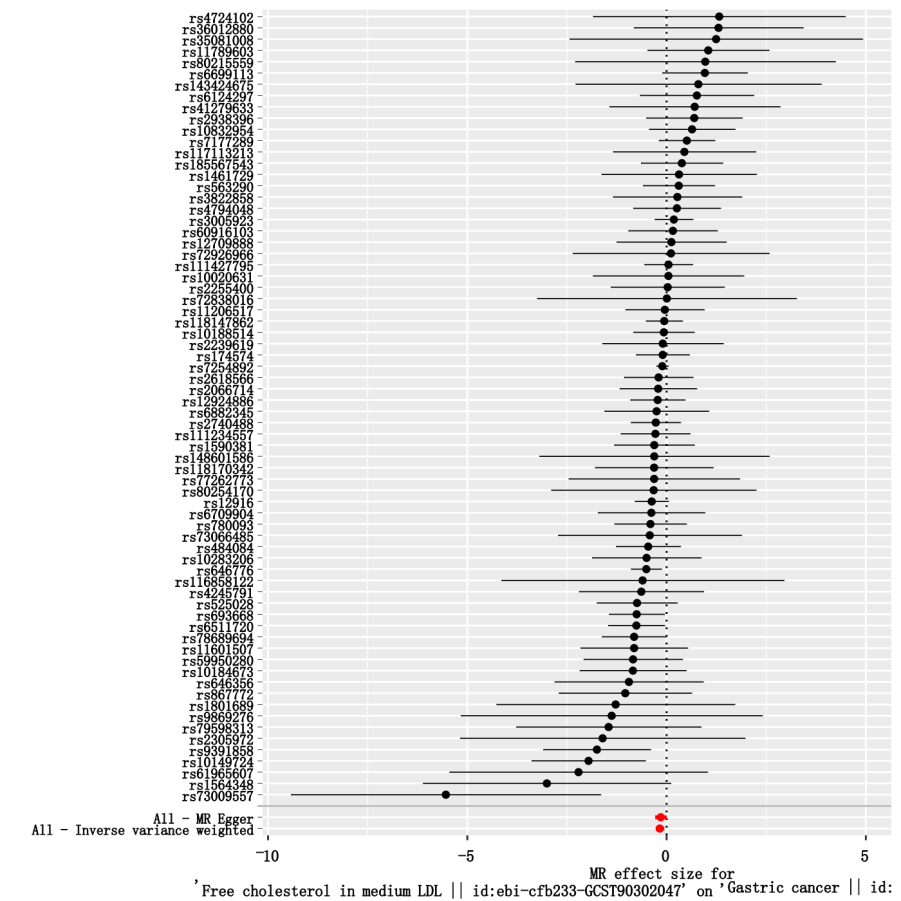

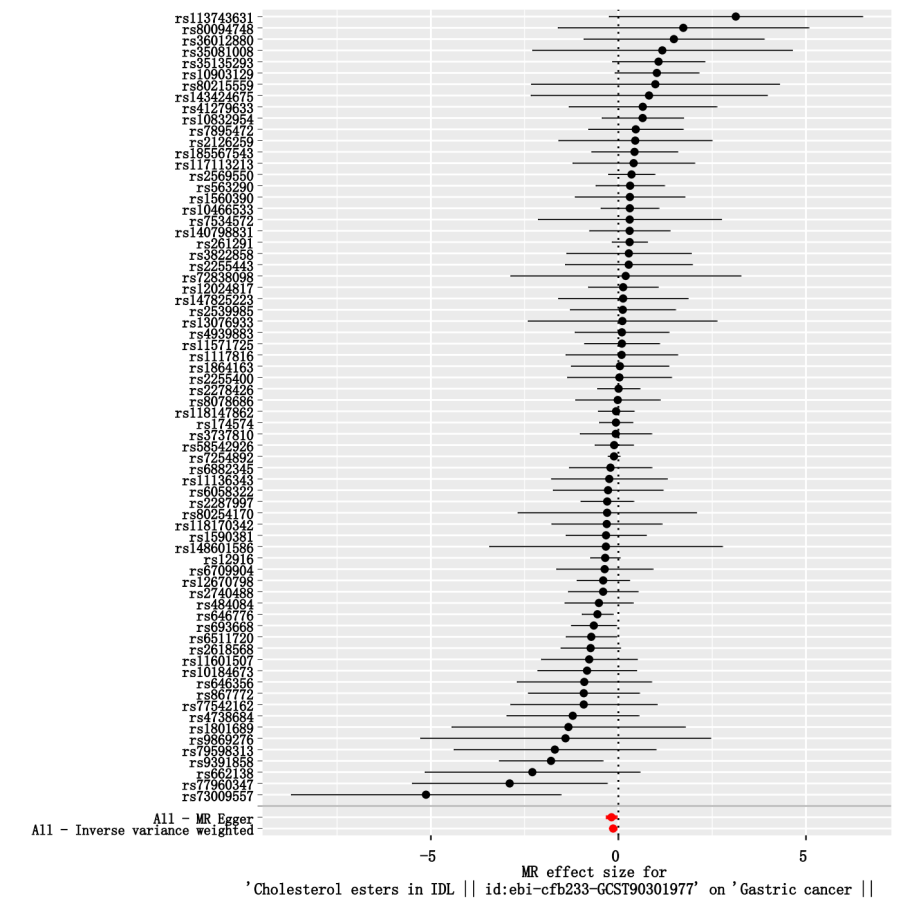


(G)

**Supplementary Figure 9: Forest plot of the causal effects of blood metabolites associated SNPs on gastric cancer.** (A) Phospholipids in small LDL; (B) Phospholipids in medium LDL; (C) Total lipids in IDL; (D) Concentration of IDL particles; (E) Free cholesterol in small LDL; (F) Free cholesterol in medium LDL; (G) Cholesterol esters in IDL.

(A) (B)


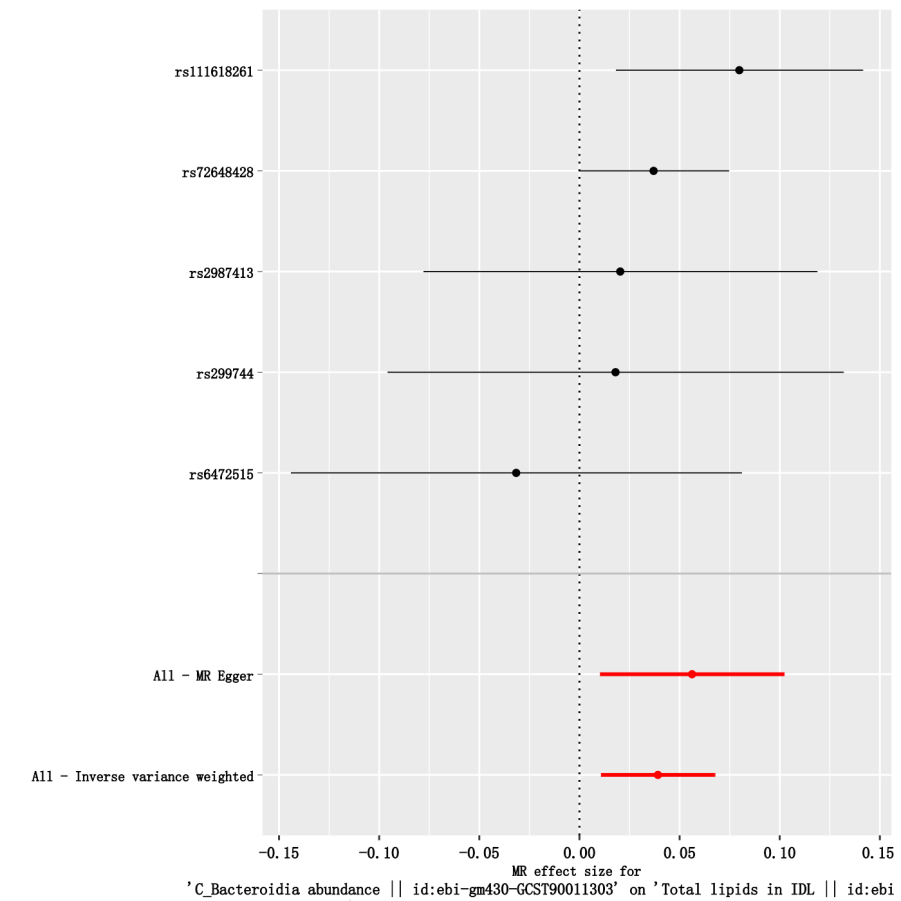

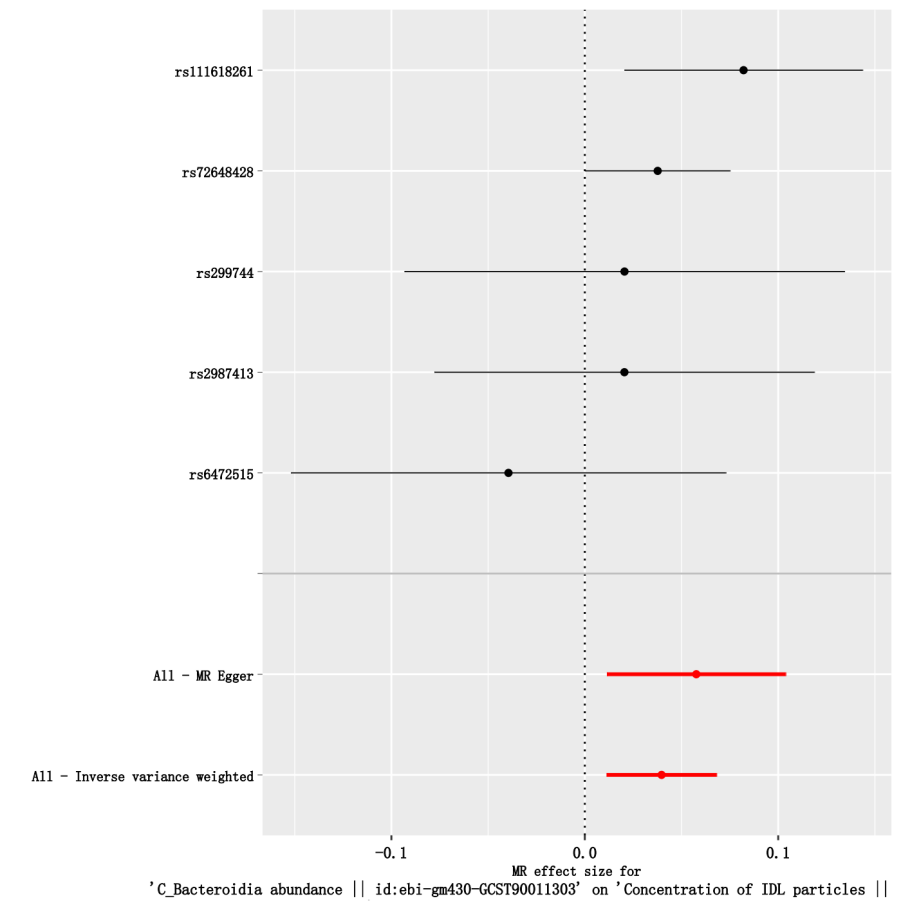

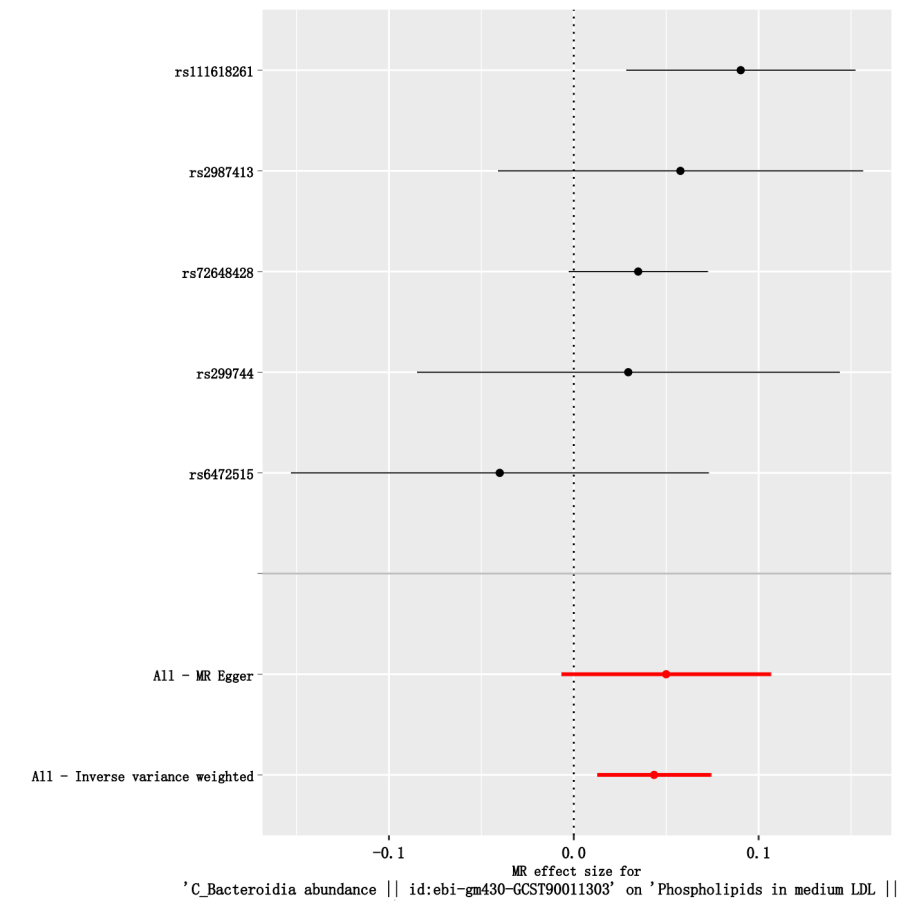

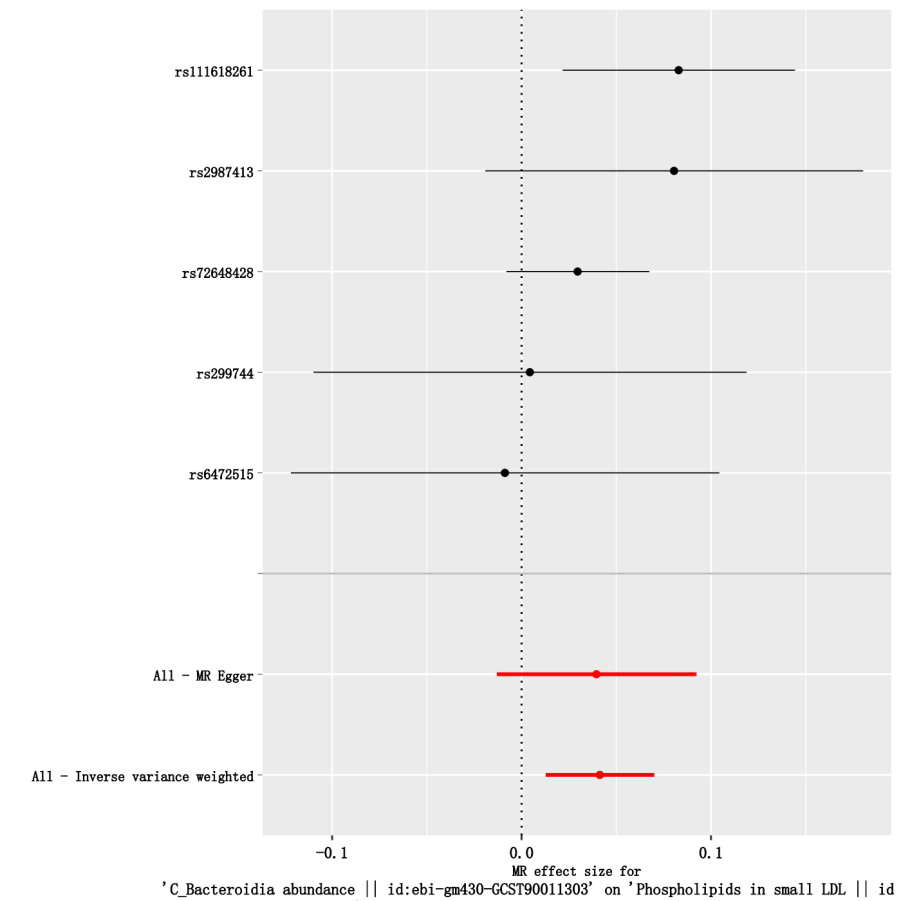


(C) (D)

1. (F)


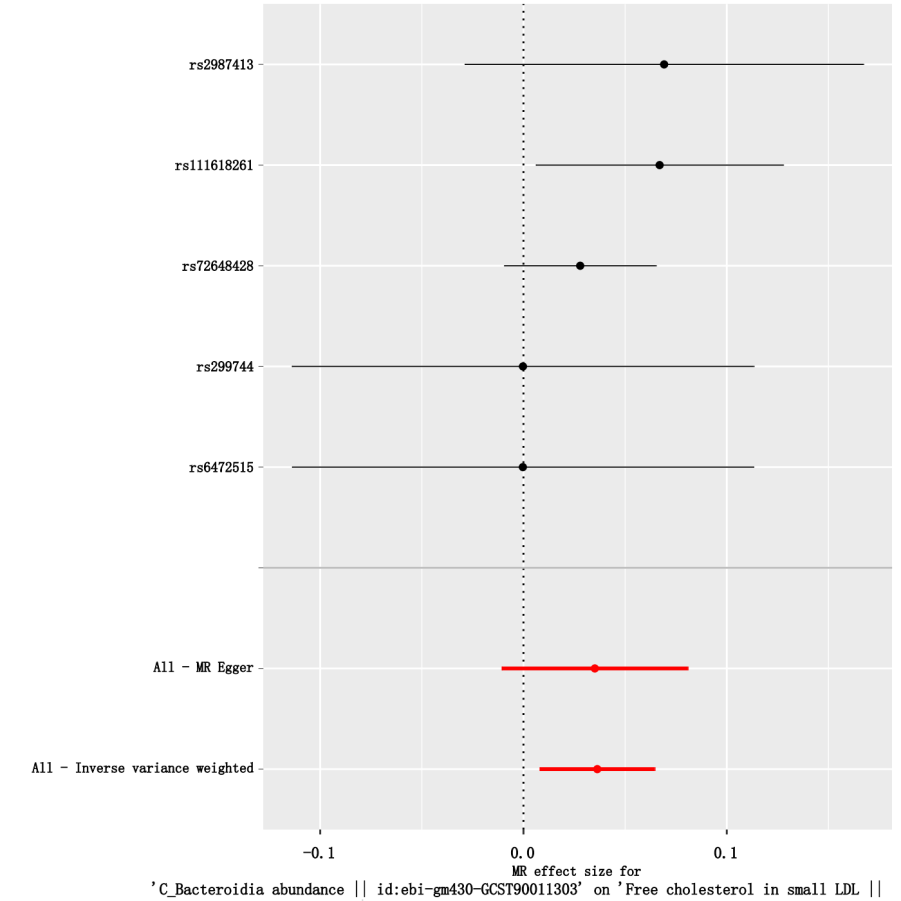

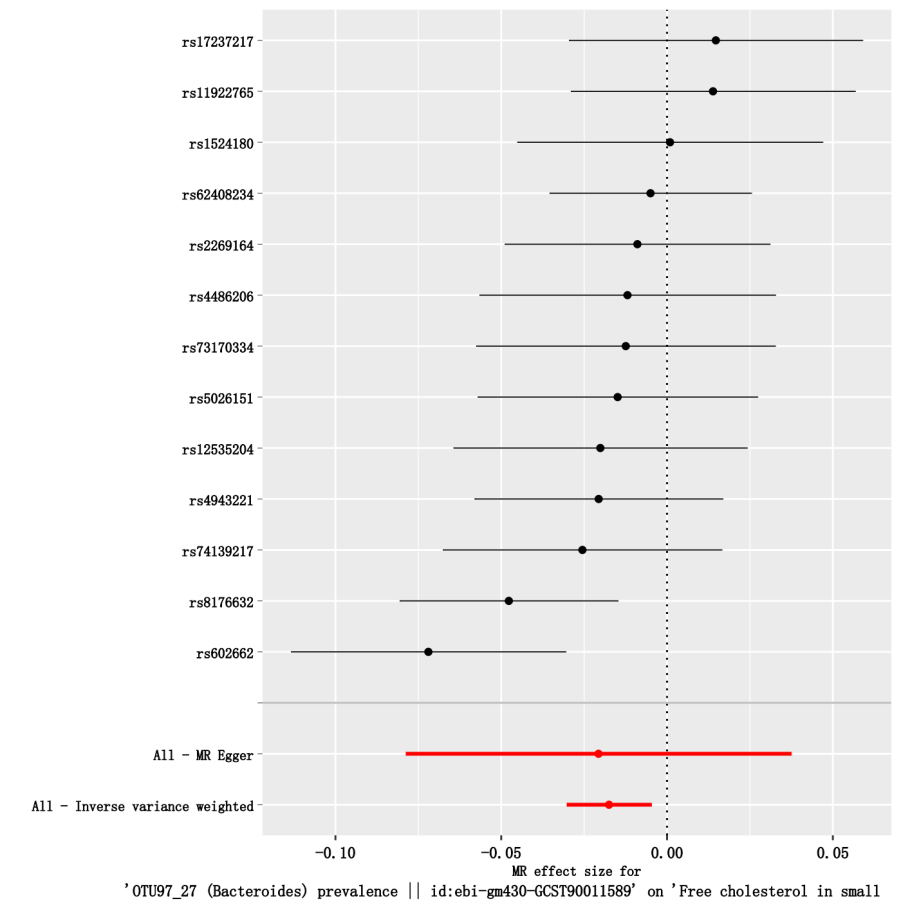


**Supplementary Figure 10: Forest plot of the causal effects of gut microbiota associated SNPs on blood metabolites.** MR for C_*Bacteroidia* on (A) Total lipids in IDL, (B) Concentration of IDL particles, (C) Phospholipids in medium LDL, (D) Phospholipids in small LDL, and (E) Free cholesterol in small LDL; MR for OTU97_27 (*Bacteroides*) on (F) Free cholesterol in small LDL.

(A) (B)


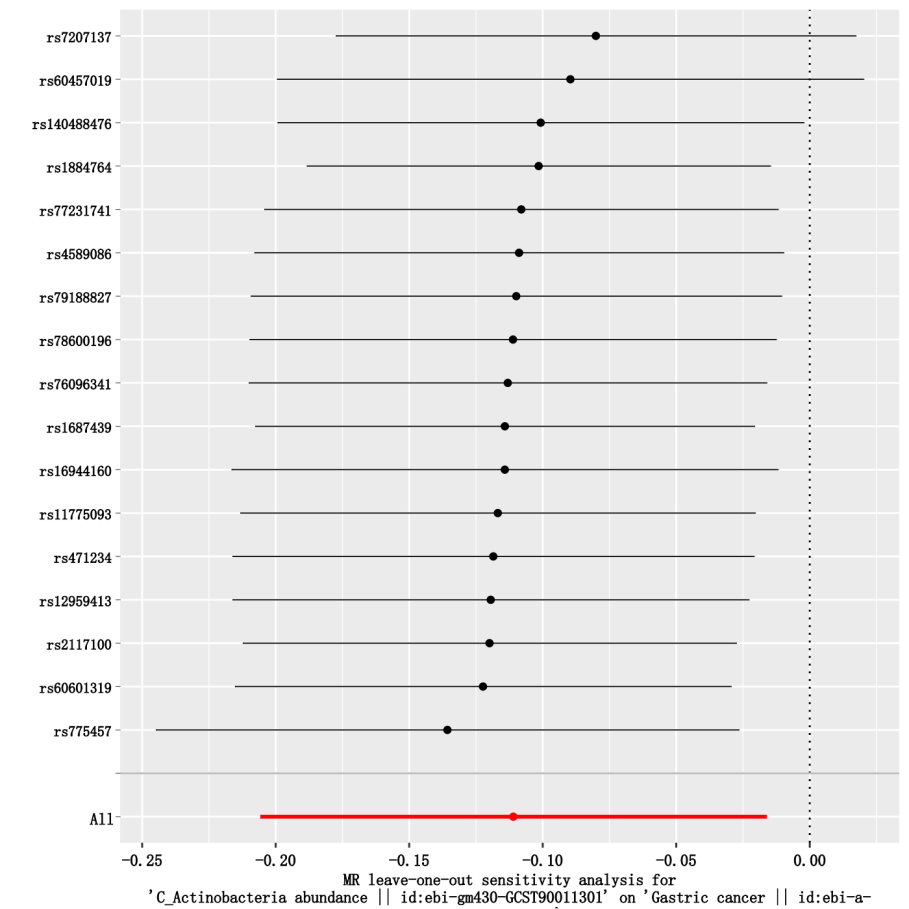

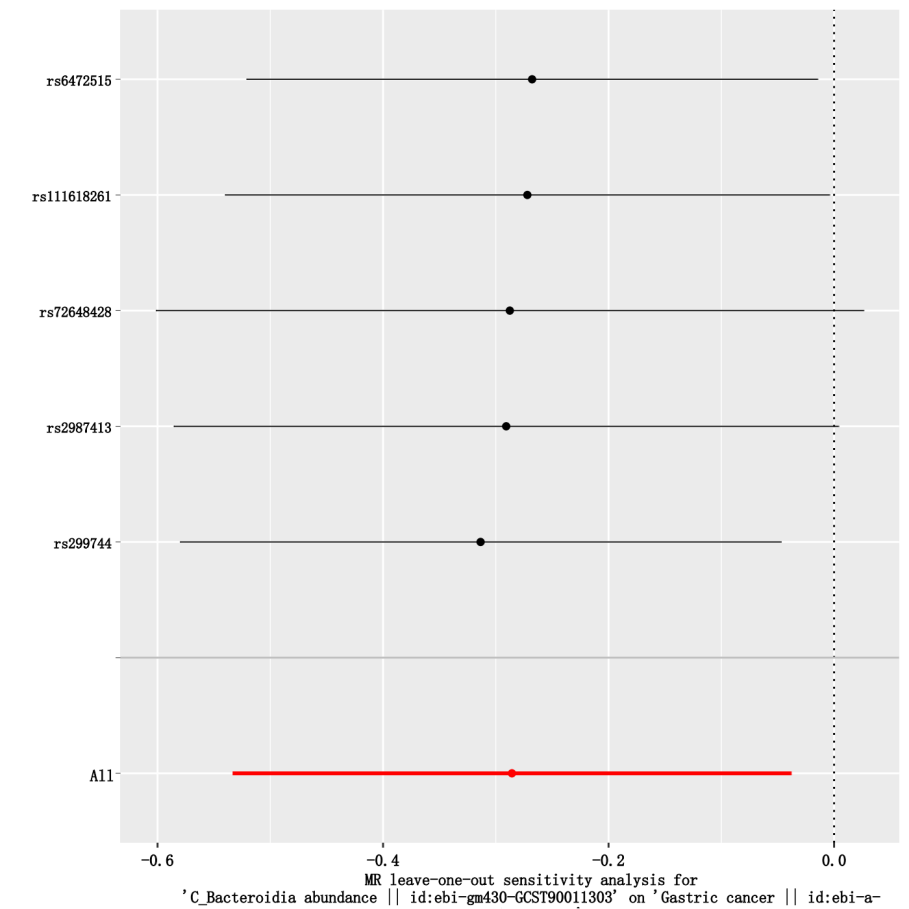

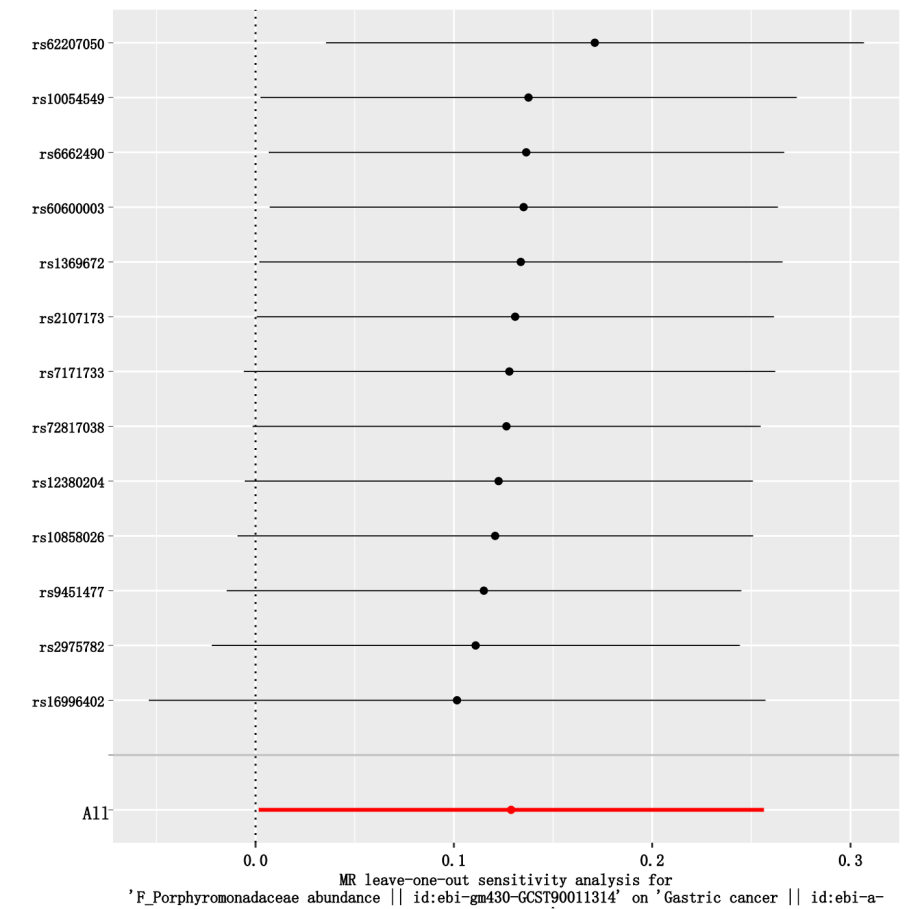


(C) (D)

(E) (F)

(G) (H)

1. (J)

(K) (L)

(M) (N)

(O) (P)

(Q) (R)

(S)

**Supplementary Figure 11: Leave-one-out sensitivity analysis of Mendelian randomization for gut microbiota and gastric cancer.** (A) C_*Actinobacteria*; (B) C_*Bacteroidia*; (C) F_*Porphyromonadaceae*; (D) G_*Clostridiales*; (E) O_*Bacteroidales*; (F) OTU97_106 (*Ruminococcaceae*); (G) OTU97_108 (*Phascolarctobacterium*); (H) OTU97_130 (*Butyrivibrio*); (I) OTU97_39 (*Proteobacteria*); (J) OTU97_56 (*Ruminococcaceae*); (K) OTU99_121 (*Ruminococcaceae*); (L) OTU99_123 (*Phascolarctobacterium*); (M) OTU99_155 (*Butyrivibrio*); (N) OTU99_40 (*Proteobacteria*); (O) P_*Actinobacteria*; (P) P_*Bacteroidetes*; (Q) TestASV_3 (*Bacteroides*); (R) OTU97_137 (*Catenibacterium*); (S) OTU97_27 (*Bacteroides*).

(A) (B)

(C) (D)

(E) (F)

(G)

**Supplementary Figure 12: Leave-one-out sensitivity analysis of Mendelian randomization for blood metabolites and gastric cancer**. (A) Cholesterol esters in IDL; (B) Total lipids in IDL; (C) Concentration of IDL particles; (D) Free cholesterol in medium LDL; (E) Phospholipids in medium LDL; (F) Free cholesterol in small LDL; (G) Phospholipids in small LDL.

(A) (B)

(C) (D)

1. (F)

**Supplementary Figure 13: Leave-one-out sensitivity analysis of Mendelian randomization (MR) for gut microbiota and blood metabolites.** MR for C_*Bacteroidia* on (A) Total lipids in IDL, (B) Concentration of IDL particles, (C) Phospholipids in medium LDL, (D) Free cholesterol in small LDL, and (E) Phospholipids in small LDL; MR for OTU97_27 (*Bacteroides*) on (F) Free cholesterol in small LDL.
